# Development of a Tool to Assess Inference-Making and Reasoning in Biology

**DOI:** 10.1128/jmbe.00159-21

**Published:** 2021-06-30

**Authors:** Jennifer G. Cromley, Ting Dai, Tia S. Fechter, Frank E. Nelson, Martin Van Boekel, Yang Du

**Affiliations:** a Department of Educational Psychology, University of Illinois at Urbana-Champaign, Champaign, Illinois, USA; b Department of Educational Psychology, University of Illinois at Chicago, Chicago, Illinois, USA; c Monterey, California, USA; d Department of Biology, Temple University, Philadelphia, Pennsylvania, USA; e Department of Educational Psychology, University of Minnesota, Minneapolis, Minnesota, USA

**Keywords:** inference, deductive, reasoning, biology, assessment, measure, predict, grade, retention, STEM

## Abstract

Making inferences and reasoning with new scientific information is critical for successful performance in biology coursework. Thus, identifying students who are weak in these skills could allow the early provision of additional support and course placement recommendations to help students develop their reasoning abilities, leading to better performance and less attrition within biology courses. Researchers across universities partnered to develop a measure to assess students’ inference-making abilities in biology. We describe the development of the inference-making and reasoning in biology assessment (IMRB). The IMRB is a 15-item multiple-choice assessment that uses short paragraphs of content—from the most-used textbook—taught at the end of a semester of survey biology courses designed for science majors. Based on our research, when the IMRB is conducted at the beginning of a semester, it measures deductive reasoning with new biology information, is fair across various student groups, and is reliable. The IMRB can be used with or without SAT or ACT scores to place students into regular undergraduate introductory biology courses, to predict grades in such courses, and/or to identify students who may need extra support or remediation in reasoning with new biology information. The IMRB is available free of charge to interested faculty and researchers.

## INTRODUCTION

Reasoning—whether it be inductive, deductive, or abductive—is essential to a career in the sciences. Indeed, empirical research on relational reasoning (1) supports the idea that reasoning is foundational and pervasive to all learning experiences and is teachable. Early identification of students who may have poor deductive reasoning skills could allow the provision of additional support and course placement recommendations that would allow these students to develop reasoning abilities, thus removing a barrier to better performance for students and aiding retention within biology courses.

More specifically, the ability to make deductive inferences—a component of the larger construct, reasoning—while reading newly presented information has been shown to be beneficial to learning, as it is associated with better reading and oral comprehension (2–4). Kintsch’s (3) construction-integration (CI) model presents three levels of comprehension, the highest of which is a situation model. Within the situation model, the reader is required to invoke during-reading strategies (e.g., summarizing, self-questioning, and making a drawing) and processes (e.g., bridging inferences and constructing elaborative inferences) that are akin to skills required for successful reasoning in STEM (science, technology, engineering, and mathematics) undergraduate coursework. While many different skills are required to be successful in introductory biology, drawing accurate conclusions from presented material is one of the most important skills, as neither instructors nor textbook authors make all relations explicit (5, 6). This means that students must draw their own conclusions (i.e., engage in inference-making) to fully understand the course material. Such inferences are critical for a deep understanding of course material and play a vital role in the transfer of learning to new contexts (7). Failure to draw such inferences is associated with poorer course grades (5, 8, 9) and lower persistence in STEM majors among students taking these biology courses. Thus, poor inference-making skills can have detrimental direct and indirect effects on course grades and persistence (10).

### Rationale for the development of the IMRB

While reasoning is foundational for all learning (1) and certainly critical to learning biology (5, 7), there are few published measures of biology reasoning. The most often used measure in biology was developed by Lawson and colleagues (11) and uses biology scenarios to test aspects of the scientific method, such as experimental design, graphing, and causality. A minority of the items test application of biological principles to specific situations, and these rely on background knowledge and multistep reasoning from such knowledge. Although content on the scientific method is part of gateway biology courses, the emphasis of such courses is reasoning with biological principles such as evolution, succession, cell signaling, alternation of generations, etc. To give one example of reasoning with biological principles that may be found in a typical textbook, students learn how mitosis is controlled by internal and external cell signals (e.g., by growth hormone) and how cancer cells do not respond to these controls. The student must draw the inference that cancerous tumors are able to grow because the cancer cells do not respond to the signaling molecules. This type of reasoning clearly differs from the type of reasoning invoked when one develops an experiment to test a hypothesis.

Researchers have also relied on measures of logical thinking to assess undergraduate biology students as a proxy for students’ scientific reasoning skills (for example, see references 12 and 13). These measures do not test application, nor are they biology specific, which reduces the validity of the measures for making inferences about biology-related reasoning skills. Therefore, developing a measure of scientific reasoning that does not rely on students’ prior knowledge is essential to help academic advisors work with students to make appropriate course selections and seek additional skill development when needed.

We present validity evidence for the inference-making and reasoning in biology assessment (IMRB), a scientific reasoning tool specifically rooted in reasoning about and with biological principles. Unlike with the widely used hypothesis‐testing skills assessment (11), the meaning of specific biological terms does not need to be known. Even if a student makes a guess at what a biological term means (e.g., lymphocyte), he or she can still reason through the answer to the question. This situation—having a tenuous grasp on new material but having to reason with it—may characterize much of learning in gateway STEM courses.

### Study AIMS

Our aim was to design an assessment to provide valid inferences for undergraduate students in introductory biology (e.g., biology, biochemistry, and other life science majors). Two specific uses include (i) prediction of gateway biology achievement and of retention in STEM for students taking such a course, usable by academic researchers and institutional research staff, and (ii) identification of students at risk of gateway biology course failure. In this paper, we describe the process used to develop the IMRB.

After designing the IMRB, we hypothesized that it would (i) predict introductory biology course grades within 4-year institutions, (ii) add incremental validity to SAT/ACT score use, (iii) have a positive correlation with retention in STEM after 2 years, and (iv) be free of items that show bias against historically underrepresented groups in STEM (i.e., females, first-generation college students, Blacks, and Hispanics). We used the IMRB to analyze several semesters of assessment data to test these hypotheses. Finally, we describe additional tools developed to enhance the usefulness of IMRB test scores, including a user manual and a course grade calculator.

## METHODS

The use of human subjects in this study complied with all relevant federal guidelines and institutional policies regarding informed consent and confidentiality. All procedures in studies involving human participants were performed in accordance with the ethical standards of the institutional and/or national research committee and with the 1964 Helsinki Declaration and its later amendments or comparable ethical standards. The studies were reviewed and approved by each institution’s institutional review board (IRB): IRB number 16896 and IRB number 23848. Informed consent was obtained from all individual participants included in the studies.

### Construct and context for IMRB development and field tests

**FIG 1:**
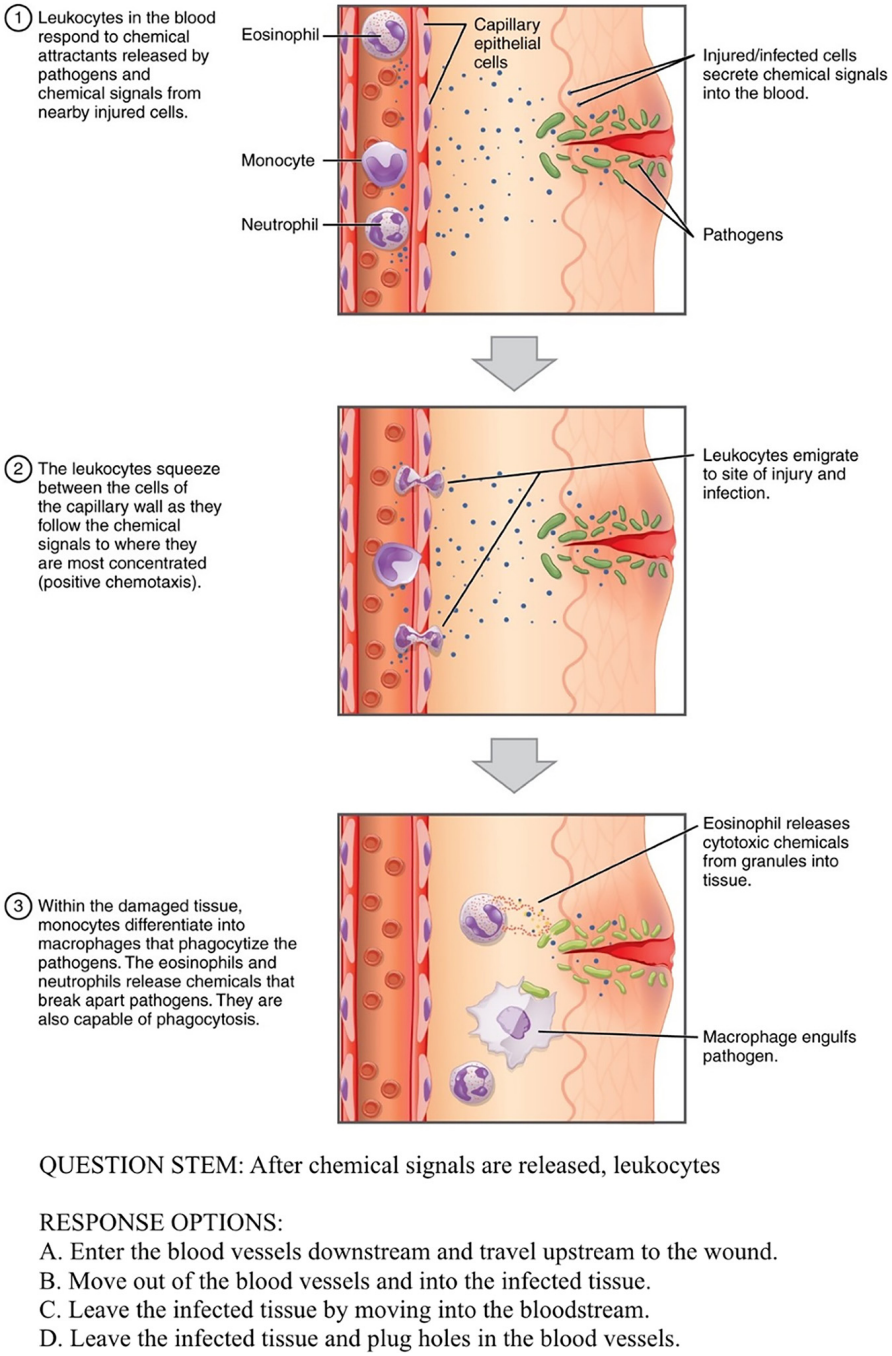
Sample IMRB test question. We added text below the original image (14) to provide an example of a test question that might accompany a graphic-based stimulus. (Image reprinted from reference 14, used under public license CC BY 4.0.)

The construct measured by the IMRB, inference-making and reasoning, is defined as applied reasoning with recently presented information. More specifically, the IMRB measures a person’s ability to use bridging inferences (3), which are the use of evidence statements and artifacts to arrive at sensible and accurate conclusions (see Fig. 1 for an example). We focused on bridging inferences because prior research with biology students (discussed later) showed that many students struggle with this type of inference. The context for this construct is undergraduate-level introductory-biology coursework for students who intend to majoring in STEM.

Several student samples were involved in the initial development, field tests, refinement, and validation studies for the IMRB (Table 1). All study participants were taking an introductory general biology course at either a large urban research university or a large flagship state university at the time of participation. Students who consented to take the IMRB received extra credit in their introductory biology courses in exchange for their participation.

**TABLE 1:** IMRB development and field tests

Semester	*n*	Development phase
2008 fall	91	Initial passage and item development
2008 fall	152	Initial field test
2009 spring	355	Initial field test
2009 fall	474	Initial field test
2010 spring	301	Initial validation administration
2010 fall	208	Initial validation administration
2011 spring	251	Initial validation administration
2015 spring	307	Initial validation administration, pretest
2015 spring	226	Initial validation administration, posttest
2016 fall	86	New passage and item development
2016 fall	267	New passage and item development
2017 fall	1,511	New field test
2017 fall	37	New validation administration
2018 spring	192	New validation administration, pretest
2018 spring	230	New validation administration, posttest

In total, 4,688 students participated in some aspect of the IMRB development. Of those, demographics are available for 1,784 students. Participants represented various races and ethnicities. Groups historically underrepresented in STEM (e.g., Black and Hispanic) made up 23% of the samples. Fifty-nine percent were females. Forty percent were first-generation college students. Unfortunately, data for a currently recognized underrepresented group (LGBTQIA+) were not collected. Data collection began in 2008, and at that time it was the convention in research to collect biological sex as a demographic variable and not to collect data on sexual orientation or gender identity.

### Development of the IMRB

The development of the IMRB was organic, with data collection beginning in fall 2008 and concluding in spring 2018. Six phases of development included passage and item development, field tests, validation administrations, new item development, new field tests, and new validation administrations. These phases resulted in two parallel (i.e., alternate) forms of the IMRB that can be used interchangeably. A brief overview of the design of the IMRB is provided, followed by the methods used within each phase for IMRB development.

### (i) IMRB design

Each item on the IMRB contains a small amount of text and sometimes an accompanying graphic (e.g., diagram). The content of each item is either taught at the end of a semester of survey biology courses designed for science majors or never taught in such courses. The stimuli are provided to examinees to read and interpret. Then, examinees are asked to respond to a multiple-choice question. These questions are designed to elicit inference-making, as the distractors contain common misconceptions students make based on the stimuli. (A description of how these misconceptions were identified is presented in “Phase I” below.) Figure 1 (14) provides an example of a graphic-based stimulus and test question.

There are currently two parallel 15-item IMRB forms. The context for both forms focuses on the immune system. Our intention is to describe the development process for the IMRB so it can be repeated to design test forms focusing on other areas of biology.

### (ii) Phase I: initial passage and item development

The first version of the IMRB was developed from student statements while reading from their biology textbook (5). Students (*n *= 91) were asked to say everything they were thinking while learning from passages about the immune system. These 40-min think-aloud sessions were recorded with participant consent. Each verbalization was coded based on the mental process or strategy each student exhibited through that verbalization—there were a total of 41 possible codes (5). More information on the coding scheme and methods (5) can be found in Appendix 2 of the supplemental material (p. 11 to 13 and 20). Students’ inferences were categorized as correct or incorrect. The resulting passages and statements were then used to create brief deductive reasoning items. The correct inferences from the think-aloud study were used as correct answers to four-option multiple-choice reasoning items, and incorrect inferences (e.g., overgeneralizations, undergeneralizations, or restatements of a premise) were used as distractors. Twenty-five items were initially developed.

### (iii) Phase II: initial field tests

All 25 items were field tested across three semesters (Table 1) with a total of 729 undergraduate introductory-biology students. Students took either a paper-based IMRB delivered within a 50-min classroom block, where no students were unable to complete the IMRB in the time allotted, or an online untimed administration, where only a few students exceeded 60 min. Items that did not perform well based on reliability analyses were discarded, resulting in the first 15 items of the IMRB. More detailed psychometric analyses can be found in Appendix 1 of the supplemental material (p. 14 to 23).

### (iv) Phase III: initial validation administrations

Both interviews and statistical analyses were conducted to support validity arguments for the use of IMRB scores.

*(a) Cognitive interviews.* Eighty-six participants were recruited from those who had completed an introductory biology course within the past 2 years. In an individual 1-h session, participants were asked to think aloud as they answered the initial 15 multiple-choice IMRB questions. Responses were audio-recorded, transcribed, and coded for item-response-strategy use based on a modified and previously published coding scheme (5), resulting in a total of 9,705 coded utterances. For each code, a within-subject analysis was used to compare the proportion of utterances verbalized when questions were answered incorrectly relative to those when questions were answered correctly in order to determine which codes were associated with correct answers. Recent research (15, 16) shows that many students take approaches to answering questions that are not focused on the use of biological principles and still arrive at the correct response despite wrong thinking. However, our findings support the idea that the IMRB questions require inference-making behavior (not just test-taking strategies or correct prior knowledge) in participating students (e.g., 201 correct inferences were verbalized when the item answer was correct versus 52 when the answer was incorrect). Verbalizing correct inferences is therefore associated with a higher probability of responding correctly to items. Again with cognitive interviews, we empirically determined that when students used methods such as relying on prior knowledge or test-taking strategies, those students were less likely to arrive at a correct response than those engaged in inference-making (5). Beyond the data, we are confident in making the claim that the IMRB targets inferencing (15), because when designing the IMRB, we used participants’ correct vocalizations of inferences and incorrect use of other approaches to develop the correct answers and distractors for each item. Therefore, we not only studied the variety of approaches participants use when presented with a given text and figure, we also used that knowledge to build quality items. That is, only the accurate use of inferencing will result in the correct response, because any other approach would result in an answer that reflects commonly held misconceptions. Thus, the IMRB test score is a good proxy for making bridging inferences (17, 18, 19). A more in-depth discussion can be found in Appendix 2 of the supplemental material (p. 7 to 8, 11 to 13, and 20).

*(b) Statistical analyses.* The IMRB user manual (20) contains technical details on statistical quality of IMRB test questions and forms. Analyses conducted include the calculation and review of item difficulty, point biserial correlations, IRT parameter estimates, differential item functioning (DIF; used to establish whether items are fair across race, sex, and socioeconomic groups), IRT model fit, and dimensionality analyses. These analyses were conducted on the 15 initial items across the various samples indicated in Table 1. Appendix 1 of the supplemental material (p.14 to 23) provides more details about the psychometric form and item analyses.

### (v) Phase IV: new passage and item development

To allow administration of the same assessment (but different items) as a pre- and posttest to the same students to determine impacts of targeted interventions, an additional and parallel form of the IMRB was developed. The same principled approach used in phase I was applied to develop new IMRB items. Additionally, the 15 old items were reviewed to uncover content-based perspectives for why those items performed well. Next, 86 think-alouds (21) were conducted on the newly selected passages. Based on the information collected, item specifications were reverse engineered for the development of a new set of 21 items to be field tested and added to the IMRB pool. Item development proceeded as described in phase I.

### (vi) Phase V: new field tests

All 21 items were field tested in fall 2017 (Table 1) with a total of 1,511 undergraduate introductory-biology students. The field test items were matrixed across several 18-item forms, where the original 15 items were administered along with three field test items. Items that did not perform well based on either reliability analyses or due to the inability to calibrate the item to the existing scale of measurement were removed from the item pool. Of the remaining items, the 15 best-performing new IMRB items were preserved (a total of 18 items were available for consideration), resulting in a total of 30 IMRB test items and two 15-item IMRB test forms.

### (vii) Phase VI: new validation administrations

Again, both cognitive interviews and statistical analyses were conducted to support validity arguments for the use of IMRB scores. Cognitive think-alouds for 18 of the new and promising IMRB items were performed with 37 students, resulting in similar support for the idea that inference-based strategies to arrive at a correct response are needed for successful completion of the IMRB. Likewise, the IMRB user manual (20) contains technical details on the statistical quality of IMRB test questions and forms.

### (viii) Finalized IMRB

The IMRB consists of two different 15-item, 1-h forms and can be used in any order. They may be administered by research or course staff and can be given on paper or on computer, to individuals or groups, once or twice per semester. Examinees (i.e., 4-year-college students planning to take or taking introductory biology) do not need any specific preparation or knowledge before taking the IMRB, as they are given all the biology information they need to reason with and are asked to draw valid conclusions from it.

The validity argument (22) for the IMRB contains details that support the validity of this test development process. Additionally, two papers (21, 23) provide supporting details regarding the cognitive interviewing approach, importance, and findings.

### Hypothesis evaluation

Four main hypotheses were evaluated.

1. The IMRB predicts introductory biology course grades within 4-year institutions.
2. The IMRB adds incremental validity to SAT/ACT score use.
3. The IMRB has a positive correlation with retention in STEM after 2 years.
4. The IMRB is free of items that show bias against historically underrepresented groups in STEM.

The first three hypotheses rely upon correlational and ordinary least-squares regression methods to inform the veracity of the statements. The fourth uses IRT-based DIF analysis to identify items that may have item bias, where an item favors one subgroup (e.g., males) over another (e.g., females). The technical steps include calibrating item parameters using a 3-parameter IRT model by constraining the model to have the same item parameter values between subgroups for items known to be free of bias and then separately calibrating the remaining items between subgroups. Finally, the improved Wald test (24) is used to test for differences for the separately calibrated items between subgroups. Three separate DIF analyses were conducted for sex, race, and college generation.

## RESULTS AND DISCUSSION

### Can IMRB scores predict introductory biology course grades?

Course grade in gateway biology courses is the outcome measure used to determine course achievement. Therefore, IMRB student scores should correlate with course grade to be considered a good predictor of achievement. Previous research shows significant correlation between the IMRB score at the beginning of the semester and the end-of-semester grade (25). For instance, greater correlation was observed between course grade and IMRB score toward the end of the semester—0.27 at week 2 and 0.55 at week 12 (17). Using the most current administrations (*n *= 196), the correlation between course grade and IMRB scores is 0.20, which is quite adequate, since more factors than the student’s ability to reason with new information inform a final course grade in introductory biology courses.

### Do IMRB scores add incremental validity to SAT/ACT score use?

The traditional role of college entrance exam (e.g., SAT/ACT) scores is to predict performance in first-year college courses; the literature especially supports SAT verbal scores as predictors of biology course grades (26). At the time our data were collected, virtually all colleges were requiring either SAT or ACT scores to be used as student selection criteria. The landscape of post-secondary education student selection has been changing over the course of our research, in that many institutions no longer require entrance exam scores. Given these changes, it is important to highlight two points with respect to the development of the IMRB. (i) In the absence of SAT and ACT scores, use of the IMRB may be seen as even more critical for addressing the needs of students selecting biology courses. That is, the IMRB is an objective and fair assessment that is directly related to a student’s ability to make inferences within a biological context and can prove quite useful for guiding students toward courses that will optimize their performance and provide support where needed. (ii) If SAT or ACT scores are available, they can complement the IMRB test scores by contributing additional information that helps predict biology course performance. Toward this end, a series of secondary regression analyses with other outcome measures shows that the use of an IMRB score results in increased ability to predict course grade. For example, including the SAT critical reading score, previous chemistry grade, and prior grade point average (GPA) as predictors of course performance, the IMRB score was still a significant independent predictor, contributing an additional 13.5% of the variance that explained course grade. Several additional regression analyses that included only the IMRB score, verbal SAT/ACT scores, and math SAT/ACT scores show the IMRB score explains additional variance (from 0.6% to 14.4%, depending on sample) associated with undergraduate introductory biology course grades. Across nine samples, the average added value of the IMRB score is the ability to explain 6.5% additional variation in course grade.

### Do IMRB scores have a positive correlation with retention in STEM after 2 years?

One use of the IMRB is to identify and support students who may be at risk for dropping out of STEM-related majors. If the IMRB score has predictive validity for retention in STEM majors, then the IMRB is useful for identifying students who might be at risk for attrition. Findings show that students with higher IMRB scores, regardless of whether they took the IMRB at the beginning or end of the semester, tended to self-report that they would likely remain in STEM, while those with lower IMRB scores at either the beginning or end of the semester tended to end the semester by self-reporting that they were unlikely to remain in STEM, explained by an indirect effect of IMRB scores on fall course grade (17). Similarly, another study showed that students who began biology with higher IMRB scores were less likely to drop out of STEM majors 2 years later (25).

### Are IMRB scores fair and just across student groups?

The IMRB is intended to support academic advising recommendations for course enrollment. However, it is not meant to exclude or prevent students from pursuing their major or field of interest. Ultimately, it is a tool to be used to help students make informed academic decisions. It is important that we evaluated whether the test items on the IMRB function differentially (differential item functioning [DIF]) among subgroups that have historically been impacted to prevent adverse academic advising decisions made based on IMRB scores. DIF analyses for the original 15 IMRB items resulted in no significant DIF between sex and ethnic or family education level groups. Likewise, additional DIF analyses were conducted for the new IMRB items in spring 2018. In this analysis, the original 15 IMRB items served as the purified anchor item set (essentially free from bias), where the item parameters remain fixed for all subgroups to stabilize the calibration within smaller subgroup samples for items being tested for DIF. This anchor set is used for testing DIF among the new items using an item response theory (IRT)-based approach (24) in which item parameters are separately calibrated for the respective subgroups and parameter estimates are tested for statistical differences (*P > *0.05). This analysis also resulted in an absence of DIF detection. However, it should be noted that one item was impossible to calibrate within the female group due to a low point biserial correlation between the item responses and the total test scores within that group. Therefore, for this one item, it was not feasible to compare male and female performance. Overall, the IMRB items chosen for operational administration are free from significant DIF for the identified subgroups of interest.

### Implications for student academic advisement

The IMRB could be instituted at the department or classroom level (see Appendix 1 in the supplemental material for more information on test administration [p. 7 and 8] and score interpretation [p. 8 to 12]). At the department level, we recommend the IMRB be administered (with supervision) before the start of the semester. Alternatively, the IMRB could be administered the first day or week of class. In either case, a student’s IMRB score could be examined in conjunction with the verbal SAT/ACT score, if available. More specifically, advisors and professors should discuss with students who have a low IMRB score or composite (SAT/ACT/IMRB) score the possibility that they may encounter coursework challenges that could result in a nonpassing course grade. It is strongly recommended that advisors and professors, together with these students, construct a proactive mitigation plan to increase the chance for success in the course. Interventions that show promise include elaborative interrogation (27), worked examples (28), prescribed active learning (26), and direct training on how to make inferences (29). While there are several interventions that show promise, more research in this area is necessary. Additionally, advisors and professors should have a separate discussion with students whose composite score suggests that they could earn a course grade in the B-C range. While a C would allow a student to proceed in the major, it could also indicate the need for a proactive mitigation plan or a shift in the student’s major concentration/emphasis. As proof of feasibility, one university is engaging in this level of academic advisement with over 1,000 entering biology majors each year.

## CONCLUSION

The IMRB is data driven and is grounded in a strong theoretical base. Therefore, various constituencies may want to know about or use the IMRB—undergraduate biology faculty (and administrators), academic advisors, researchers in biology education and education disciplines, and university institutional research staff—as it is designed to identify students who may struggle with making bridging inferences (see Fig. 1 for an example) and is grounded in Kintsch’s construction-integration model (3).

One strength of the IMRB is that items were developed from interviews with undergraduate students learning from illustrated biology texts. The incorrect answer choices on the IMRB are actual reasoning errors verbalized by target students when faced with new biology information. A different set of students was then interviewed as they answered finalized IMRB items, and correct answers came from using the reasoning skills that the IMRB is designed to test, not from test-taking strategies, knowledge, or other strategies.

Based on our research, when administered as directed, the IMRB is indeed reliable, it measures deductive reasoning with new biology information, and it is fair across various student groups. Since inference-making skills are teachable, IMRB test scores are best used for advising students on course placement, remediation, and interventions that support optimal academic outcomes for students.

## OTHER SUPPLEMENTAL TOOLS

To complement the IMRB test forms, there exists a user manual and a predicted course grade calculator.

### User manual

The user manual provides the information that university personnel need to consider before adopting the IMRB. It explicitly states appropriate and inappropriate uses, provide test administration directions and options, explain the development and evaluation of the IMRB, provides validity evidence in support of its appropriate uses, and guidance on how to expand upon the existing IMRB for research and operational uses. It also provides all the test items in an appendix. Ultimately, the manual serves to help university personnel interpret test results. For questions or concerns regarding appropriate use and expansion, please contact the corresponding author.

### Course grade calculator

**FIG 2:**
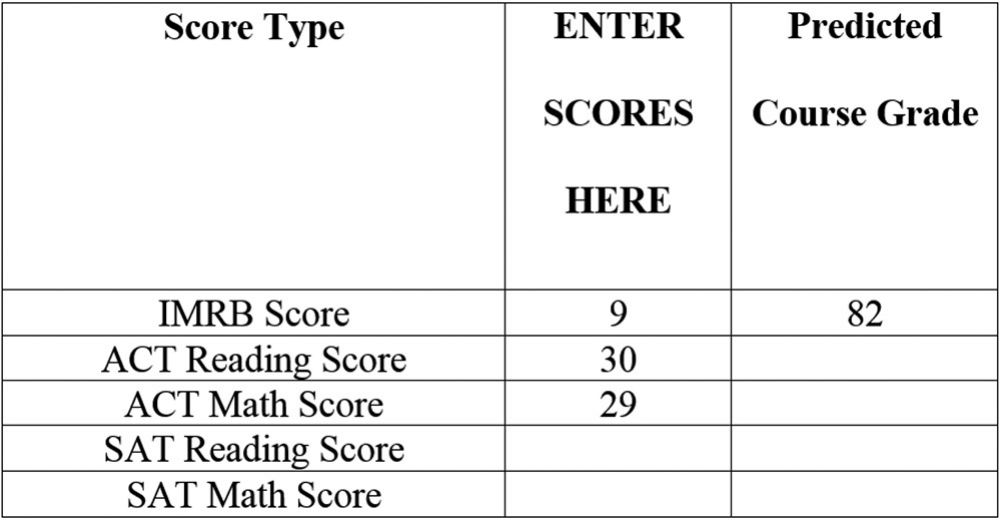
Course grade calculator example.

The course grade calculator is a spreadsheet-based tool designed for use by university personnel. Available scores can be entered, and the most appropriate prediction model will be selected and implemented to obtain a student’s predicted course grade. Once scores are input, the calculator applies the most appropriate linear regression weights to predict the final introductory biology course grade for a student. The tool looks similar to the image in Fig. 2. The example provided indicates that a student earned a score of 9 on the IMRB, 30 on the ACT reading test, and 29 on the ACT math test, resulting in a predicted course grade of an 82. Note that the IMRB can also be used in the absence of SAT/ACT scores to predict course grades with efficacy; however, the most accurate predictions result when either SAT or ACT scores are also available.

## References

[1] Alexander PA, Jablansky S, Singer LM, Dumas D. 2016. Relational reasoning: what we know and why it matters. Policy Insights Behav Brain Sci 3:36–44. doi:10.1177/2372732215622029.

[2] Butcher KR. 2006. Learning from text with diagrams: promoting mental model development and inference generation. J Educ Psychol 98:182–197. doi:10.1037/0022-0663.98.1.182.

[3] Kintsch W. 1998. Comprehension: a paradigm for cognition. Cambridge University Press, Cambridge, United Kingdom.

[4] McNamara DS. 2004. SERT: self-explanation reading training. Discourse Process 38:1–30. doi:10.1207/s15326950dp3801_1.

[5] Cromley JG, Snyder-Hogan LE, Luciw-Dubas UA. 2010. Cognitive activities in complex science text and diagrams. Contemp Educ Psychol 35:59–74. doi:10.1016/j.cedpsych.2009.10.002.

[6] Otero J, León JA, Graesser AC (ed). 2002. The psychology of science text comprehension. Lawrence Erlbaum Associates Inc., Mahwah, NJ.

[7] Cromley JG, Bergey BW, Fitzhugh S, Newcombe N, Wills TW, Shipley TF, Tanaka JC. 2013. Effects of three diagram instruction methods on transfer of diagram comprehension skills: the critical role of inference while learning. Learn Instr 26:45–58. doi:10.1016/j.learninstruc.2013.01.003.

[8] Ozuru Y, Dempsey K, McNamara DS. 2009. Prior knowledge, reading skill, and text cohesion in the comprehension of science texts. Learn Instr 19:228–242. doi:10.1016/j.learninstruc.2008.04.003.

[9] Van Den Broek P, Virtue S, Everson MG, Tzeng Y, Sung YC. 2002. Comprehension and memory of science texts: inferential processes and the construction of a mental representation, p 131–154. In Otero J, Leon JA, Graesser AC (ed), The psychology of science text comprehension. Lawrence Erlbaum Associates Inc., Mahwah, NJ.

[10] Maltese AV, Tai RH. 2011. Pipeline persistence: examining the association of educational experiences with earned degrees in STEM among US students. Sci Ed 95:877–907. doi:10.1002/sce.20441.

[11] Lawson AE, Banks DL, Logvin M. 2007. Self-efficacy, reasoning ability and achievement in college biology. J Res Sci Teach 44:706–724. doi:10.1002/tea.20172.

[12] Hurst RW, Milkent MM. 1996. Facilitating successful prediction problem solving in biology through application of skill theory. J Res Sci Teach 33:541–552. doi:10.1002/(SICI)1098-2736(199605)33:5<541::AID-TEA5>3.0.CO;2-R.

[13] Kılıç D, Sağlam N. 2014. Students’ understanding of genetics concepts: the effect of reasoning ability and learning approaches. J Biol Educ 48:63–70. doi:10.1080/00219266.2013.837402.

[14] Betts JG, Young KA, Wise JA, Johnson E, Poe B, Kruse DH, Korol O, Johnson JE, Womble M, DeSaix P. 2013. Anatomy and physiology. OpenStax. https://openstax.org/books/anatomy-and-physiology/pages/preface.

[15] Sung R, Swarat SL, Lo SM. 2020. Doing coursework without doing biology: undergraduate students’ non-conceptual strategies to problem solving. J Biol Educ 2020:1785925. doi:10.1080/00219266.2020.1785925.

[16] Sato BK, Hill CF, Lo SM. 2019. Testing the test: are exams measuring understanding? Biochem Mol Biol Educ 47:296–302. doi:10.1002/bmb.21231. 30844134

[17] Cromley JG, Ma S, Van Boekel M, Dane N. 2020. Pickup of causal language and inference during and after reading illustrated text. Read Psychol 41:57–182. doi:10.1080/02702711.2020.1768974.

[18] Cromley JG, Dane P, Fechter T, Dai T, Nelson FE, Zelenka E, Shah J. 2020. Cognitive interviews on new biology reasoning items—inference, yes, test-taking strategies, no. Presented at the Annual Meeting of the American Educational Research Association, San Francisco, CA.

[19] Cromley JG, Fechter T, Van Boekel M, Dai T, Nelson F, Parpucu AN. 2018. Think-alouds provide supporting validity evidence: expected cognitive processes are measured. Presented at the Institute of Educational Sciences Annual Principal Investigators Meeting, Arlington, VA.

[20] Cromley JG, Fechter TS, Dai T, Nelson F. 2020. Inference-making and reasoning in biology (IMRB) user manual & technical report. http://hdl.handle.net/2142/110042. 10.1128/jmbe.00159-21 PMC8442032 34594465

[21] Cromley JG, Dai T, Fechter T, Van Boekel M, Nelson FE, Dane N. 2021. What cognitive interviewing reveals about a new measure of undergraduate biology reasoning. J Exp Educ 89:145–168. doi:10.1080/00220973.2019.1613338.

[22] Fechter TM, Dai T, Cromley JG, Van Boekel M, Nelson F. 2020. Developing a validity argument for an inference-making and reasoning measure. Presented at the annual meeting of the American Educational Research Association, San Francisco, CA.

[23] Dai T, Boekel M, Cromley J, Nelson F, Fechter T. 2018. Using think-alouds to create a better measure of biology reasoning. SAGE Res Methods Cases. doi:10.4135/9781526437167.

[24] Woods CM, Cai L, Wang M. 2013. The Langer-improvedWald test for DIF testing with multiple groups: evaluation and comparison to two-group IRT. Educ Psychol Meas 73:532–547. doi:10.1177/0013164412464875.

[25] Dai T, Cromley JG. 2014. Changes in implicit theories of ability in biology and dropout from STEM majors: a latent growth curve approach. Contemp Educ Psychol 39:233–247. doi:10.1016/j.cedpsych.2014.06.003.

[26] Freeman S, O'Connor E, Parks JW, Cunningham M, Hurley D, Haak D, Dirks C, Wenderoth MP. 2007. Prescribed active learning increases performance in Introductory Biology. CBE Life Sci Educ 6:132–139. doi:10.1187/cbe.06-09-0194. 17548875 PMC1885904

[27] Seifert TL. 1993. Effects of elaborative interrogation with prose passages. J Educ Psychol 85:642–651. doi:10.1037/0022-0663.85.4.642.

[28] Dyer JO, Hudon A, Montpetit-Tourangeau K, Charlin B, Mamede S, van Gog T. 2015. Example-based learning: comparing the effects of additionally providing three different integrative learning activities on physiotherapy intervention knowledge. BMC Med Educ 15:37. doi:10.1186/s12909-015-0308-3. 25889066 PMC4414367

[29] Elleman A. 2017. Examining the impact of inference instruction on the literal and inferential comprehension of skilled and less skilled readers: a meta-analytic review. J Educ Psychol 109:761–781. doi:10.1037/edu0000180.

