# Supplementary material for: Development of a Tool to Assess Inference-Making and Reasoning in Biology
Source: J Microbiol Biol Educ. 2021 Jun 30;22(2):e00159-21. doi: 10.1128/jmbe.00159-21 (PMC8442032; doi:10.1128/jmbe.00159-21)
Supplement: SUPPLEMENTAL FILE 1 — Download JMBE00159-21_Supp_1_seq4.docx, DOCX file, 10.8 MB [file jmbe00159-21_supp_1_seq4.docx]

Supplemental Materials

Development of a Tool to Assess Inference-Making and Reasoning in Biology (IMRB)

Jennifer G. Cromley^1^, Ph.D. (PI); Ting Dai^2^, Ph.D. (Co-PI); Tia S. Fechter^3^, Ed.D. (Co-PI);
Frank E. Nelson^4^, Ph.D. (Co-PI); Martin Van Boekel^5^, Ph.D.; Yang Du^1^, M.S.Ed., M.S.

*^1^Department of Educational Psychology, University of Illinois at Urbana-Champaign, Champaign, IL 61820*

*^2^Department of Educational Psychology, University of Illinois at Chicago, Chicago, IL 60607*

*^3^Sole Proprietor*

*^4^Department of Biology, Temple University, Philadelphia, PA 19122*

*^5^Department of Educational Psychology, University of Minnesota, Minneapolis MN 55455*

Appendix 1: Inference-Making and Reasoning in Biology (IMRB) User Manual & Technical Report.

Appendix 2: IMRB Validity Framework Documentation.

Appendix 3: Course Grade Calculator.

Dear Reader,

These supplemental materials are meant to further inform you and the institution you represent about the usefulness of the Inference-Making and Reasoning in Biology (IMRB) tool as you consider whether you would like to adopt the use of the measure. For your convenience, we identify specific audiences for each resource included here.

Appendix 1: Inference-Making and Reasoning in Biology (IMRB) User Manual & Technical Report.

This resource is provided as an implementation guide for using the IMRB. It also provides concrete support for the IMRB development and use. It serves to explicitly state appropriate and inappropriate uses, provide test administration directions and options, explain the development and evaluation of the IMRB, provide validity evidence in support of its appropriate uses, and provide guidance on how to expand upon the existing IMRB for research and operational uses. Additionally, this manual serves to help university personnel interpret test results.

This manual is accessible to university personnel who would make final decisions on IMRB implementation and those who would be responsible for its administration to students.

Appendix 2: IMRB Validity Framework Documentation.

This resource is provided to supplement the IMRB User Manual & Technical Report by supplying detailed validity evidence for its supported uses. The IMRB Validity Framework Documentation is intended for university personnel who would be responsible for assessing the quality of the IMRB for adoption at their institution.

Appendix 3: Course Grade Calculator.

The Course Grade Calculator is intended to help university advisors provide some context for how student reasoning skills, as measured by the IMRB, may impact performance in introductory biology courses. This resource is provided for use by university personnel responsible for score interpretation and those directly providing guidance to students on course placement, choice of major, and additional interventions they may need.

Appendix 1:

Inference-Making and Reasoning in Biology (IMRB) User Manual & Technical Report

Inference-Making and Reasoning in Biology (IMRB) User Manual & Technical Report

Jennifer G. Cromley, PI, University of Illinois at Urbana-Champaign, Department of Educational Psychology

Tia S. Fechter, Co-PI, Sole Proprietor

Ting Dai, Co-PI, University of Illinois at Chicago, Department of Educational Psychology

Frank E. Nelson, Co-PI, Temple University, Department of Biology

The research reported here was supported by the Institute of Education Sciences, U.S. Department of Education, through Grant R305A160335 to University of Illinois, Urbana-Champaign. The opinions expressed are those of the authors and do not represent views of the Institute or the U.S. Department of Education.

# Purpose of This Manual

This manual provides the information necessary for university personnel to consider before making a decision to adopt the use of the Inference-Making and Reasoning in Biology (IMRB) assessment—an assessment of deductive reasoning with newly-presented biology information. It serves to explicitly state appropriate and inappropriate uses, provide test administration directions and options, explain the development and evaluation of the IMRB, provide validity evidence in support of its appropriate uses, and provide guidance on how to expand upon the existing IMRB for research and operational uses. Additionally, this manual serves to help university personnel interpret test results.

Additional resources include the IMRB Validity Framework which this manual references and a test score interpretation spreadsheet tool. These resources should be consulted as necessary.

For questions or concerns regarding appropriate use and expansion, please contact Jennifer G. Cromley at [jcromley@illinois.edu](mailto:jcromley@illinois.edu).

# Motivation for IMRB Development

Researchers across universities partnered to develop a measure to assess students’ inference-making and reasoning abilities in biology. The need for the measure is the finding that many undergraduate students fail to perform well in freshman biology courses—one-half of students drop out of life sciences majors and most after their first year (National Science Foundation [NSF], 2006). One possible cause of low performance and attrition is that these students are not adept at drawing inferences from material used in classes and from textbooks (Cromley, Snyder-Hogan, & Luciw-Dubas, 2010). Identifying these students early could allow for the provision of additional support and course placement recommendations that would allow students to develop reasoning abilities, leading to better performance and less attrition within biology courses.

# Overview of IMRB

The IMRB is a 15-item multiple-choice assessment that uses short paragraphs of content—from the most used textbook—taught at the end of a semester of survey biology courses designed for science majors (e.g., biology, biochemistry, neuroscience). The specific content area of the measure is the immune system.

Table of Contents

[Purpose of This Manual 2](file:///C:\Users\Keith\Desktop\TF%20paper\JMBE_additional_resources\IMRB%20User%20Manual.docx#_Toc49788727)

[Motivation for IMRB Development 2](file:///C:\Users\Keith\Desktop\TF%20paper\JMBE_additional_resources\IMRB%20User%20Manual.docx#_Toc49788728)

[Overview of IMRB 2](file:///C:\Users\Keith\Desktop\TF%20paper\JMBE_additional_resources\IMRB%20User%20Manual.docx#_Toc49788729)

[1. Executive Summary 5](file:///C:\Users\Keith\Desktop\TF%20paper\JMBE_additional_resources\IMRB%20User%20Manual.docx#_Toc49788730)

[2. Appropriate and Inappropriate Uses 6](file:///C:\Users\Keith\Desktop\TF%20paper\JMBE_additional_resources\IMRB%20User%20Manual.docx#_Toc49788731)

[Table 2-1. IMRB: Summary of Appropriate and Inappropriate Uses 6](file:///C:\Users\Keith\Desktop\TF%20paper\JMBE_additional_resources\IMRB%20User%20Manual.docx#_Toc49788732)

[3. Validity Evidence 7](file:///C:\Users\Keith\Desktop\TF%20paper\JMBE_additional_resources\IMRB%20User%20Manual.docx#_Toc49788733)

[Table 3-1. IMRB: Summary of Validity Evidence 7](file:///C:\Users\Keith\Desktop\TF%20paper\JMBE_additional_resources\IMRB%20User%20Manual.docx#_Toc49788734)

[4. Test Administration 7](file:///C:\Users\Keith\Desktop\TF%20paper\JMBE_additional_resources\IMRB%20User%20Manual.docx#_Toc49788735)

[Who? 7](file:///C:\Users\Keith\Desktop\TF%20paper\JMBE_additional_resources\IMRB%20User%20Manual.docx#_Toc49788736)

[When? 8](file:///C:\Users\Keith\Desktop\TF%20paper\JMBE_additional_resources\IMRB%20User%20Manual.docx#_Toc49788737)

[How? 8](file:///C:\Users\Keith\Desktop\TF%20paper\JMBE_additional_resources\IMRB%20User%20Manual.docx#_Toc49788738)

[Scoring 8](file:///C:\Users\Keith\Desktop\TF%20paper\JMBE_additional_resources\IMRB%20User%20Manual.docx#_Toc49788739)

[5. Score Interpretation 8](file:///C:\Users\Keith\Desktop\TF%20paper\JMBE_additional_resources\IMRB%20User%20Manual.docx#_Toc49788740)

[IMRB Cut Matrix 9](file:///C:\Users\Keith\Desktop\TF%20paper\JMBE_additional_resources\IMRB%20User%20Manual.docx#_Toc49788741)

[Table 5-1. IMRB Cut Matrix for ACT Reading 10](file:///C:\Users\Keith\Desktop\TF%20paper\JMBE_additional_resources\IMRB%20User%20Manual.docx#_Toc49788742)

[Table 5-2. IMRB Cut Matrix for ACT Math and SAT Math 11](file:///C:\Users\Keith\Desktop\TF%20paper\JMBE_additional_resources\IMRB%20User%20Manual.docx#_Toc49788743)

[Table 5-3. Regression Coefficients and R-Square for Course Grade Prediction Models 12](file:///C:\Users\Keith\Desktop\TF%20paper\JMBE_additional_resources\IMRB%20User%20Manual.docx#_Toc49788744)

[Course Grade Calculator 12](file:///C:\Users\Keith\Desktop\TF%20paper\JMBE_additional_resources\IMRB%20User%20Manual.docx#_Toc49788745)

[Figure 5-1. Course Grade Calculator Example 12](file:///C:\Users\Keith\Desktop\TF%20paper\JMBE_additional_resources\IMRB%20User%20Manual.docx#_Toc49788746)

[6. Construct Definition 12](file:///C:\Users\Keith\Desktop\TF%20paper\JMBE_additional_resources\IMRB%20User%20Manual.docx#_Toc49788747)

[7. Item Development 12](file:///C:\Users\Keith\Desktop\TF%20paper\JMBE_additional_resources\IMRB%20User%20Manual.docx#_Toc49788748)

[8. Psychometric Form Analysis 14](file:///C:\Users\Keith\Desktop\TF%20paper\JMBE_additional_resources\IMRB%20User%20Manual.docx#_Toc49788749)

[Descriptive Statistics 14](file:///C:\Users\Keith\Desktop\TF%20paper\JMBE_additional_resources\IMRB%20User%20Manual.docx#_Toc49788750)

[Table 8-1. Descriptive Statistics for Form A and Form B 14](file:///C:\Users\Keith\Desktop\TF%20paper\JMBE_additional_resources\IMRB%20User%20Manual.docx#_Toc49788751)

[Reliability 14](file:///C:\Users\Keith\Desktop\TF%20paper\JMBE_additional_resources\IMRB%20User%20Manual.docx#_Toc49788752)

[Standard error of measurement 15](file:///C:\Users\Keith\Desktop\TF%20paper\JMBE_additional_resources\IMRB%20User%20Manual.docx#_Toc49788753)

[Dimensionality Analysis 15](file:///C:\Users\Keith\Desktop\TF%20paper\JMBE_additional_resources\IMRB%20User%20Manual.docx#_Toc49788754)

[Test Characteristic Curves, Test Information Functions, and Conditional Standard Error of Measurement 16](file:///C:\Users\Keith\Desktop\TF%20paper\JMBE_additional_resources\IMRB%20User%20Manual.docx#_Toc49788755)

[Figure 8-1. IMRB Form A and Form B Test Characteristic Curves, Information, and Conditional Standard Error of Measurement 17-19](file:///C:\Users\Keith\Desktop\TF%20paper\JMBE_additional_resources\IMRB%20User%20Manual.docx#_Toc49788756)

[Form Equivalence and Quality 19](file:///C:\Users\Keith\Desktop\TF%20paper\JMBE_additional_resources\IMRB%20User%20Manual.docx#_Toc49788757)

[Table 8-2. Form A and Form B Psychometric Characteristics 19](file:///C:\Users\Keith\Desktop\TF%20paper\JMBE_additional_resources\IMRB%20User%20Manual.docx#_Toc49788758)

[9. Psychometric Item Analysis 19](file:///C:\Users\Keith\Desktop\TF%20paper\JMBE_additional_resources\IMRB%20User%20Manual.docx#_Toc49788759)

[*p*-value 19](file:///C:\Users\Keith\Desktop\TF%20paper\JMBE_additional_resources\IMRB%20User%20Manual.docx#_Toc49788760)

[*Point Biserial Correlation* 20](file:///C:\Users\Keith\Desktop\TF%20paper\JMBE_additional_resources\IMRB%20User%20Manual.docx#_Toc49788761)

[*Factor Loadings* 20](file:///C:\Users\Keith\Desktop\TF%20paper\JMBE_additional_resources\IMRB%20User%20Manual.docx#_Toc49788762)

[*IRT Parameter Estimates* 20](file:///C:\Users\Keith\Desktop\TF%20paper\JMBE_additional_resources\IMRB%20User%20Manual.docx#_Toc49788763)

[*Differential Item Functioning* 21](file:///C:\Users\Keith\Desktop\TF%20paper\JMBE_additional_resources\IMRB%20User%20Manual.docx#_Toc49788764)

[Table 9-1. IMRB Item Statistics 23](file:///C:\Users\Keith\Desktop\TF%20paper\JMBE_additional_resources\IMRB%20User%20Manual.docx#_Toc49788765)

[10. Future IMRB Development 24](file:///C:\Users\Keith\Desktop\TF%20paper\JMBE_additional_resources\IMRB%20User%20Manual.docx#_Toc49788766)

[A Note on Missing Data 24](file:///C:\Users\Keith\Desktop\TF%20paper\JMBE_additional_resources\IMRB%20User%20Manual.docx#_Toc49788767)

[References 25](file:///C:\Users\Keith\Desktop\TF%20paper\JMBE_additional_resources\IMRB%20User%20Manual.docx#_Toc49788768)

# 1. Executive Summary

The Inference-Making and Reasoning in Biology (IMRB) measure was designed and validated with funds from the US Department of Education, Institute for Education Sciences (IES). The goal of our undergraduate biology reasoning project was three-fold:

1. To develop a measure that is reliable, valid, and fair across male/female, URM/non-URM, and first-generation college/not first-generation students.
2. To document that the measure can predict undergraduate introductory biology course grades

Two different 15-item, 1-hour forms were developed (Section 7), and they can be used in any order. They may be administered by any research or course staff, and can be given on paper or on computer, to individuals or groups, once or twice per semester (Section 4). Appropriate examinees (4-year college students planning to take or taking introductory biology) do not need to have any specific preparation or knowledge before taking the IMRB, as they are given all the biology information they need to reason with and are asked to draw valid conclusions with that information (Section 4).

Various constituencies may want to know about or use the IMRB—undergraduate biology faculty (and administrators), academic advisors, researchers in biology education and education disciplines, and university institutional research staff.

One strength of the IMRB is that items were developed from interviews with undergraduate students learning from illustrated biology texts. The incorrect answer choices on the IMRB are actual reasoning errors verbalized by undergraduate biology students when faced with new biology information (Section 7). A different set of students was then interviewed as they answered finalized IMRB items, and correct answers came from using the reasoning that the IMRB tests, not from test-taking strategies, knowledge, or other strategies (Section 7).

Based on our research, when administered as directed, the IMRB is indeed reliable—students answer in an internally consistent way (Section 8)—it measures deductive reasoning with new biology information (Section 8), and it is fair across various student groups (Section 9). As with all measures, it is suitable for certain specific uses, and unsuitable for other uses (Section 2).

The IMRB can be used together with SAT or ACT scores to place students into regular undergraduate introductory biology courses, to predict grades in such courses, and/or to identify students who may need extra supports or remediation in reasoning with new biology information (Section 5).

The IMRB can be used in limited settings to track growth in reasoning; specifically, it can be used when a biology course specifically focuses on building reasoning skills. This cannot simply be an implicit aim or hope of the instructor—direct instruction in learning how to reason with biology information must be part of the course before the IMRB can be used in a valid way to track growth in reasoning (Section 2).

There are various limitations to how the IMRB should be used, and we detail these in Table 2-1. More generally, the IMRB should not be used as the only measure to make consequential decisions for students, it should not be used to evaluate instructor or program performance, and results should not be used to predict anything but undergraduate introductory biology grades (Section 2).

# 2. Appropriate and Inappropriate Uses

Based on 9 studies at 2 universities over 3 years, we have evidence to support appropriate uses presented in the first column of Table 2-1. Additionally, based on national standards for fair testing and the above studies, we caution that some uses may be inappropriate (as presented in the second column of Table 2-1) as they lack validity evidence.

### Table 2-1. IMRB: Summary of Appropriate and Inappropriate Uses

| Appropriate Uses  (paper or computer administration, group or individual administration) | Inappropriate Uses  (unless or until evidence is gathered to support validity for these purposes) |
| --- | --- |
| - Together with ACT or SAT scores, to place students into regular introductory undergraduate biology courses without any remedial work on biology reasoning. - **Exception:** Students who sincerely try to answer all questions and obtain a score of 2, 1, or zero should not be placed in regular introductory undergraduate biology courses without any remedial work on biology reasoning, regardless of ACT or SAT scores. - To identify students possibly at risk of undergraduate introductory biology course failure, in order to provide supplemental help - For research purposes, as a predictor of student course grades—with or without other measures—provided the IMRB is administered within the first 2 weeks of a regular semester (14-16 weeks) | - To place students into regular introductory undergraduate biology courses based on the IMRB scores alone, without taking account of ACT or SAT scores. - To exclude students in order to reduce class size or other non-academic reasons - To inform students whether they are “suited” for biology as a discipline - To track growth in student reasoning - To evaluate faculty work individually or as a department, to reward or punish biology instructors or to make decisions about teaching assignments - To directly predict whether students might remain in a STEM major - To predict scores on other tests (besides course grade) - To “stand for” or measure general reasoning, reasoning in domains other than biology, or to measure learning ability or anything other than reasoning with new biology information - As part of assigning grades in a course, or to use instead of instruction - To make any other coursework placement, scholarship/funding, program continuance, or other consequential decisions other than as noted in *Appropriate uses* - To use with 2-year college or high school, or non-US undergraduate biology students - To make any decisions based on improper administration of the IMRB (e.g., completed collaboratively or with help, given as a “take home”, used as “practice” in a class meeting, etc.) |

# 3. Validity Evidence

Validity evidence to support appropriate IMRB uses is provided throughout this manual. However, a comprehensive validity narrative should be obtained (Fechter et. al., 2020) to further explore the evidence that supports IMRB appropriate uses. Validity evidence presented in this manual relates to

- Test design and development;
- Test administration;
- Test scoring; and
- Psychometric evidence of test quality.

Table 3-1 summarizes the validity evidence for the IMRB appropriate uses provided in each section of this manual.

### Table 3-1. IMRB: Summary of Validity Evidence

| *Type of Validity Evidence* | *Section* | *Description of Information Provided* |
| --- | --- | --- |
| Reliability and classical item analyses; scoring consistency and classification consistency | 8 | Classical item analyses; Overall reliability and standard error of measurement; reliability by student subgroups; Decision accuracy and consistency (DAC) |
| Content-related validity evidence | 7 | Test blueprints; item alignment to test blueprints and standards |
| Construct-related and structural validity evidence | 9 | Response process validity evidence: item response theory modeling; dimensionality; scaling; linking; differential item functioning; item bias review and procedures; cognitive interviewing/think alouds |
| Consequential validity | 5 | Supporting the valid use of IMRB data; interpreting scores |

# 4. Test Administration

This section provides test administrators with directions for administering the IMRB; and is organized around who the assessment was designed for and who is qualified to administered it, when the assessment should be given, how it should be administered, and how to score the assessment.

## Who?

The IMRB will provide valid results for introductory biology students at 4-year US colleges and universities.

Any instructor/TA, researcher (including student research assistants), or research or course staff may administer the IMRB, as no special knowledge or skills are needed to administer it. Anyone administering the measure is responsible for test security (e.g., not allowing copies to leave the room, to be reproduced by examinees).

## When?

The IMRB may be given up to twice per (14-16) week semester, the first time within the first 2 weeks of the semester. If given at the beginning of the semester, it may be given again within the last 2 weeks of the semester—provided reasoning and logic skills are explicitly taught during the semester. If given only once, give as a pretest.

The IMRB was designed as an untimed assessment. However, research shows that allowing at least 1 hour for completion is sufficient time for most examinees to finish the assessment. For students who completed all items, their mean response time is 33.53 minutes (*SD* = 34.95). For students who did not complete the test forms, their mean response time is 12.51 minutes (*SD* = 21.69). Based on these descriptive statistics, it’s clear that students’ not completing the test forms might be attributed to low-motivation (quitting in the middle of the test) instead of running out of time.

The IMRB should be given in a single session, not allowing partial completion and then a break before fully completing the measure.

## How?

The IMRB may be given in paper or electronic format, in individual or whole-class administration. Scantron sheets may be used to collect responses.

To protect the integrity of the assessment, the IMRB must not be given as a paper measure to be taken out of the classroom (i.e., a “take home”), nor may it be used as “practice” to be discussed.

Students may backtrack, check and/or change previous answers. They may write on the test sheet (e.g., eliminating options they believe are incorrect).

Students must work alone and without assistance from people or any other resources.

Students may not copy questions from the IMRB.

The items on “Form A” and “Form B” have been carefully selected based on various statistical criteria; items should not be selected from or re-arranged between forms. Doing so will invalidate the cut scores used for making guidance and placement decisions.

Scores may be discussed with examinees, but answers to individual questions should not be discussed with them, nor should examines see whether any particular question was answered correctly or incorrectly.

## Scoring

Simply sum up the number of correctly-answered questions to derive an examinee’s score.

# 5. Score Interpretation

This section discusses how to interpret IMRB scores alongside ACT or SAT scores, when available. Two tools were developed to help aid IMRB test score users—both are based on the same ordinary least squares (OLS) regression-based prediction models. The first we refer to as the IMRB Cut Matrix and the second we refer to as the Course Grade Calculator. Each is described next.

## IMRB Cut Matrix

The IMRB Cut Matrix contains two matrices where 1) provides predicted course grades based on ACT Reading and IMRB scores (Table 5-1) and 2) provides predicted course grades based on ACT Math and IMRB scores (Table 5-2). SAT equivalents are available for the Math ACT scores but not for the Reading ACT scores because as of 2018, the SAT conversion table includes the sum of ACT Reading and ACT English scores.^[[1]](#footnote-1)^ IMRB studies, to date, have only collected ACT Reading scores—not ACT English scores.

### Table 5-1. IMRB Cut Matrix for ACT Reading

|  | IMRB Score | | | | | | | | | | | | | | | |
| --- | --- | --- | --- | --- | --- | --- | --- | --- | --- | --- | --- | --- | --- | --- | --- | --- |
| ACT_Read | 0 | 1 | 2 | 3 | 4 | 5 | 6 | 7 | 8 | 9 | 10 | 11 | 12 | 13 | 14 | 15 |
| 1 | 48 | 49 | 49 | 50 | 50 | 51 | 51 | 52 | 53 | 53 | 54 | 54 | 55 | 56 | 56 | 57 |
| 2 | 49 | 49 | 50 | 51 | 51 | 52 | 52 | 53 | 54 | 54 | 55 | 55 | 56 | 57 | 57 | 58 |
| 3 | 50 | 50 | 51 | 52 | 52 | 53 | 53 | 54 | 55 | 55 | 56 | 56 | 57 | 58 | 58 | 59 |
| 4 | 51 | 51 | 52 | 53 | 53 | 54 | 54 | 55 | 56 | 56 | 57 | 57 | 58 | 59 | 59 | 60 |
| 5 | 52 | 52 | 53 | 54 | 54 | 55 | 55 | 56 | 57 | 57 | 58 | 58 | 59 | 60 | 60 | 61 |
| 6 | 53 | 53 | 54 | 55 | 55 | 56 | 56 | 57 | 58 | 58 | 59 | 59 | 60 | 61 | 61 | 62 |
| 7 | 54 | 54 | 55 | 56 | 56 | 57 | 57 | 58 | 59 | 59 | 60 | 60 | 61 | 61 | 62 | 63 |
| 8 | 55 | 55 | 56 | 57 | 57 | 58 | 58 | 59 | 60 | 60 | 61 | 61 | 62 | 62 | 63 | 64 |
| 9 | 56 | 56 | 57 | 58 | 58 | 59 | 59 | 60 | 60 | 61 | 62 | 62 | 63 | 63 | 64 | 65 |
| 10 | 57 | 57 | 58 | 59 | 59 | 60 | 60 | 61 | 61 | 62 | 63 | 63 | 64 | 64 | 65 | 66 |
| 11 | 58 | 58 | 59 | 59 | 60 | 61 | 61 | 62 | 62 | 63 | 64 | 64 | 65 | 65 | 66 | 67 |
| 12 | 59 | 59 | 60 | 60 | 61 | 62 | 62 | 63 | 63 | 64 | 65 | 65 | 66 | 66 | 67 | 68 |
| 13 | 60 | 60 | 61 | 61 | 62 | 63 | 63 | 64 | 64 | 65 | 66 | 66 | 67 | 67 | 68 | 69 |
| 14 | 61 | 61 | 62 | 62 | 63 | 64 | 64 | 65 | 65 | 66 | 67 | 67 | 68 | 68 | 69 | 70 |
| 15 | 62 | 62 | 63 | 63 | 64 | 65 | 65 | 66 | 66 | 67 | 68 | 68 | 69 | 69 | 70 | 71 |
| 16 | 63 | 63 | 64 | 64 | 65 | 66 | 66 | 67 | 67 | 68 | 69 | 69 | 70 | 70 | 71 | 71 |
| 17 | 64 | 64 | 65 | 65 | 66 | 67 | 67 | 68 | 68 | 69 | 70 | 70 | 71 | 71 | 72 | 72 |
| 18 | 65 | 65 | 66 | 66 | 67 | 68 | 68 | 69 | 69 | 70 | 70 | 71 | 72 | 72 | 73 | 73 |
| 19 | 66 | 66 | 67 | 67 | 68 | 69 | 69 | 70 | 70 | 71 | 71 | 72 | 73 | 73 | 74 | 74 |
| 20 | 67 | 67 | 68 | 68 | 69 | 69 | 70 | 71 | 71 | 72 | 72 | 73 | 74 | 74 | 75 | 75 |
| 21 | 68 | 68 | 69 | 69 | 70 | 70 | 71 | 72 | 72 | 73 | 73 | 74 | 75 | 75 | 76 | 76 |
| 22 | 68 | 69 | 70 | 70 | 71 | 71 | 72 | 73 | 73 | 74 | 74 | 75 | 76 | 76 | 77 | 77 |
| 23 | 69 | 70 | 71 | 71 | 72 | 72 | 73 | 74 | 74 | 75 | 75 | 76 | 77 | 77 | 78 | 78 |
| 24 | 70 | 71 | 72 | 72 | 73 | 73 | 74 | 75 | 75 | 76 | 76 | 77 | 78 | 78 | 79 | 79 |
| 25 | 71 | 72 | 73 | 73 | 74 | 74 | 75 | 76 | 76 | 77 | 77 | 78 | 79 | 79 | 80 | 80 |
| 26 | 72 | 73 | 74 | 74 | 75 | 75 | 76 | 77 | 77 | 78 | 78 | 79 | 80 | 80 | 81 | 81 |
| 27 | 73 | 74 | 75 | 75 | 76 | 76 | 77 | 78 | 78 | 79 | 79 | 80 | 80 | 81 | 82 | 82 |
| 28 | 74 | 75 | 76 | 76 | 77 | 77 | 78 | 79 | 79 | 80 | 80 | 81 | 81 | 82 | 83 | 83 |
| 29 | 75 | 76 | 77 | 77 | 78 | 78 | 79 | 79 | 80 | 81 | 81 | 82 | 82 | 83 | 84 | 84 |
| 30 | 76 | 77 | 78 | 78 | 79 | 79 | 80 | 80 | 81 | 82 | 82 | 83 | 83 | 84 | 85 | 85 |
| 31 | 77 | 78 | 78 | 79 | 80 | 80 | 81 | 81 | 82 | 83 | 83 | 84 | 84 | 85 | 86 | 86 |
| 32 | 78 | 79 | 79 | 80 | 81 | 81 | 82 | 82 | 83 | 84 | 84 | 85 | 85 | 86 | 87 | 87 |
| 33 | 79 | 80 | 80 | 81 | 82 | 82 | 83 | 83 | 84 | 85 | 85 | 86 | 86 | 87 | 88 | 88 |
| 34 | 80 | 81 | 81 | 82 | 83 | 83 | 84 | 84 | 85 | 86 | 86 | 87 | 87 | 88 | 89 | 89 |
| 35 | 81 | 82 | 82 | 83 | 84 | 84 | 85 | 85 | 86 | 87 | 87 | 88 | 88 | 89 | 90 | 90 |
| 36 | 82 | 83 | 83 | 84 | 85 | 85 | 86 | 86 | 87 | 88 | 88 | 89 | 89 | 90 | 90 | 91 |

### Table 5-2. IMRB Cut Matrix for ACT Math and SAT Math

|  | IMRB Score | | | | | | | | | | | | | | | |  |
| --- | --- | --- | --- | --- | --- | --- | --- | --- | --- | --- | --- | --- | --- | --- | --- | --- | --- |
| ACT_Math | 0 | 1 | 2 | 3 | 4 | 5 | 6 | 7 | 8 | 9 | 10 | 11 | 12 | 13 | 14 | 15 | **SAT** |
| 1 | 41 | 41 | 42 | 43 | 44 | 44 | 45 | 46 | 46 | 47 | 48 | 48 | 49 | 50 | 50 | 51 |  |
| 2 | 42 | 43 | 43 | 44 | 45 | 45 | 46 | 47 | 47 | 48 | 49 | 49 | 50 | 51 | 51 | 52 |  |
| 3 | 43 | 44 | 45 | 45 | 46 | 47 | 47 | 48 | 49 | 49 | 50 | 51 | 51 | 52 | 53 | 53 |  |
| 4 | 44 | 45 | 46 | 46 | 47 | 48 | 48 | 49 | 50 | 51 | 51 | 52 | 53 | 53 | 54 | 55 |  |
| 5 | 46 | 46 | 47 | 48 | 48 | 49 | 50 | 50 | 51 | 52 | 52 | 53 | 54 | 54 | 55 | 56 |  |
| 6 | 47 | 48 | 48 | 49 | 50 | 50 | 51 | 52 | 52 | 53 | 54 | 54 | 55 | 56 | 56 | 57 |  |
| 7 | 48 | 49 | 49 | 50 | 51 | 51 | 52 | 53 | 53 | 54 | 55 | 55 | 56 | 57 | 58 | 58 |  |
| 8 | 49 | 50 | 51 | 51 | 52 | 53 | 53 | 54 | 55 | 55 | 56 | 57 | 57 | 58 | 59 | 59 |  |
| 9 | 50 | 51 | 52 | 52 | 53 | 54 | 55 | 55 | 56 | 57 | 57 | 58 | 59 | 59 | 60 | 61 |  |
| 10 | 52 | 52 | 53 | 54 | 54 | 55 | 56 | 56 | 57 | 58 | 58 | 59 | 60 | 60 | 61 | 62 | **260** |
| 11 | 53 | 54 | 54 | 55 | 56 | 56 | 57 | 58 | 58 | 59 | 60 | 60 | 61 | 62 | 62 | 63 | **280** |
| 12 | 54 | 55 | 55 | 56 | 57 | 57 | 58 | 59 | 59 | 60 | 61 | 62 | 62 | 63 | 64 | 64 | **310** |
| 13 | 55 | 56 | 57 | 57 | 58 | 59 | 59 | 60 | 61 | 61 | 62 | 63 | 63 | 64 | 65 | 65 | **330** |
| 14 | 57 | 57 | 58 | 59 | 59 | 60 | 61 | 61 | 62 | 63 | 63 | 64 | 65 | 65 | 66 | 67 | **360** |
| 15 | 58 | 58 | 59 | 60 | 60 | 61 | 62 | 62 | 63 | 64 | 64 | 65 | 66 | 66 | 67 | 68 | **400** |
| 16 | 59 | 60 | 60 | 61 | 62 | 62 | 63 | 64 | 64 | 65 | 66 | 66 | 67 | 68 | 68 | 69 | **430** |
| 17 | 60 | 61 | 61 | 62 | 63 | 63 | 64 | 65 | 66 | 66 | 67 | 68 | 68 | 69 | 70 | 70 | **470** |
| 18 | 61 | 62 | 63 | 63 | 64 | 65 | 65 | 66 | 67 | 67 | 68 | 69 | 69 | 70 | 71 | 71 | **500** |
| 19 | 63 | 63 | 64 | 65 | 65 | 66 | 67 | 67 | 68 | 69 | 69 | 70 | 71 | 71 | 72 | 73 | **510** |
| 20 | 64 | 64 | 65 | 66 | 66 | 67 | 68 | 68 | 69 | 70 | 70 | 71 | 72 | 73 | 73 | 74 | **520** |
| 21 | 65 | 66 | 66 | 67 | 68 | 68 | 69 | 70 | 70 | 71 | 72 | 72 | 73 | 74 | 74 | 75 | **530** |
| 22 | 66 | 67 | 68 | 68 | 69 | 70 | 70 | 71 | 72 | 72 | 73 | 74 | 74 | 75 | 76 | 76 | **540** |
| 23 | 67 | 68 | 69 | 69 | 70 | 71 | 71 | 72 | 73 | 73 | 74 | 75 | 75 | 76 | 77 | 77 | **560** |
| 24 | 69 | 69 | 70 | 71 | 71 | 72 | 73 | 73 | 74 | 75 | 75 | 76 | 77 | 77 | 78 | 79 | **580** |
| 25 | 70 | 70 | 71 | 72 | 72 | 73 | 74 | 75 | 75 | 76 | 77 | 77 | 78 | 79 | 79 | 80 | **590** |
| 26 | 71 | 72 | 72 | 73 | 74 | 74 | 75 | 76 | 76 | 77 | 78 | 78 | 79 | 80 | 80 | 81 | **610** |
| 27 | 72 | 73 | 74 | 74 | 75 | 76 | 76 | 77 | 78 | 78 | 79 | 80 | 80 | 81 | 82 | 82 | **640** |
| 28 | 73 | 74 | 75 | 75 | 76 | 77 | 77 | 78 | 79 | 79 | 80 | 81 | 81 | 82 | 83 | 84 | **660** |
| 29 | 75 | 75 | 76 | 77 | 77 | 78 | 79 | 79 | 80 | 81 | 81 | 82 | 83 | 83 | 84 | 85 | **680** |
| 30 | 76 | 76 | 77 | 78 | 79 | 79 | 80 | 81 | 81 | 82 | 83 | 83 | 84 | 85 | 85 | 86 | **700** |
| 31 | 77 | 78 | 78 | 79 | 80 | 80 | 81 | 82 | 82 | 83 | 84 | 84 | 85 | 86 | 86 | 87 | **710** |
| 32 | 78 | 79 | 80 | 80 | 81 | 82 | 82 | 83 | 84 | 84 | 85 | 86 | 86 | 87 | 88 | 88 | **720** |
| 33 | 79 | 80 | 81 | 81 | 82 | 83 | 83 | 84 | 85 | 86 | 86 | 87 | 88 | 88 | 89 | 90 | **740** |
| 34 | 81 | 81 | 82 | 83 | 83 | 84 | 85 | 85 | 86 | 87 | 87 | 88 | 89 | 89 | 90 | 91 | **760** |
| 35 | 82 | 83 | 83 | 84 | 85 | 85 | 86 | 87 | 87 | 88 | 89 | 89 | 90 | 91 | 91 | 92 | **780** |
| 36 | 83 | 84 | 84 | 85 | 86 | 86 | 87 | 88 | 88 | 89 | 90 | 90 | 91 | 92 | 92 | 93 | **800** |

Table 5.1 and 5.2 provide the predicted course grade for each ACT and IMRB score combination. That is, Table 5.1 uses ACT Reading and IMRB scores to predict introductory biology course grade; and Table 5.2 uses ACT Math (or SAT Math) and IMRB scores to predict introductory biology course grade. Highlighted in dark yellow are the cells across the matrix where course grade is expected to be a 70 (or equivalent to a grade of C in most universities). Highlighted in light green are the cells across the matrix where course grade is expected to an 80 (or equivalent to a grade of B in most universities). Table 5.3 provides the regression coefficients and R-square estimates for each of the prediction models and also includes the model where all predictors are used (i.e., IMRB score, ACT Reading, and ACT Math are used in the Course Grade Calculator—see next section) and for when only an IMRB score is available (i.e., IMRB Only).

### Table 5-3. Regression Coefficients and R-Square for Course Grade Prediction Models

| Model | Intercept | IMRB | ACT Reading | ACT Math | R^2^ |
| --- | --- | --- | --- | --- | --- |
| **IMRB Only** | 68.6810 | 1.1150 | **---** | **---** |  |
| **IMRB Cut Matrix for ACT Reading** | 46.9314 | 0.5926 | 0.9796 | --- | 0.27 |
| **IMRB Cut Matrix for ACT Math** | 39.6147 | 0.6751 | --- | 1.2064 | 0.26 |
| **Course Grade Calculator** | 38.2848 | 0.5346 | 0.6096 | 0.6998 | 0.30 |

## Course Grade Calculator

The Course Grade Calculator is tool designed for use by university personnel where within a spreadsheet, scores available can be entered and then the most appropriate prediction model will be selected and implemented to obtain a student’s predicted course grade. Within Excel, the tool looks similar to the image in Figure 5-1. The example provided indicates a student earned a score of 9 on the IMRB, 30 on the ACT Reading test, and 29 on the ACT Math test, resulting in a predicted course grade of an 82.

| Score Type | ENTER SCORES HERE | Predicted Course Grade |
| --- | --- | --- |
| IMRB Score | 9 | 82 |
| ACT Reading Score | 30 |  |
| ACT Math Score | 29 |  |
| SAT Reading Score |  |  |
| SAT Math Score |  |  |

### Figure 5-1. Course Grade Calculator Example

# 6. Construct Definition

The construct measured by the IMRB is defined as applied reasoning with recently presented information (i.e., given evidence statements and artifacts, the ability to use those statements and artifacts to arrive at sensible and accurate conclusions). The context for this construct is undergraduate-level introductory biology coursework. The IMRB measure is intended to provide valid inferences for undergraduate students in introductory biology who mostly represent biology, biochemistry, and other biology-related majors.

# 7. Item Development

The IMRB is designed to measure deductive reasoning from newly-presented biology information. The reasoning is deductive in that conclusions can be drawn from two pieces of presented information; this is distinguished from inductive reasoning or pattern detection, which is also important for biology learning. No information from prior knowledge is needed, beyond the most basic information such as “cells make up tissues.”

The assessment tasks for the IMRB are short paragraphs of content taught at the end of a semester of survey biology courses designed for science majors (e.g., biology, biochemistry, neuroscience). The stimuli are provided to examinees to read and then examinees are asked to respond to multiple-choice questions. The multiple-choice questions are designed to elicit inference-making and reasoning skills as the distractors contain common misconceptions that students make based on the stimuli.

The first version of the IMRB was developed from student statements while reading from their biology textbook (Cromley, Snyder-Hogan, & Luciw-Dubas, 2010). Students (*n* = 91) were asked to say everything they were thinking while learning from passages about the immune system, and—among other codes—students’ inferences were categorized as correct or incorrect. The think-aloud sessions were 40-minutes in length. The resulting passages and statements were then used to create brief deductive reasoning items, where the correct inferences from the think-aloud study were used as correct answers to 4-option multiple-choice reasoning items, and incorrect inferences (e.g., over-generalizations, under-generalizations, or restatements of a premise) were used as distractors.

Twenty-five items were initially developed and piloted with 737 undergraduate introductory biology students. Items that did not perform well in reliability analysis were deleted, resulting in the first 15 items of the IMRB.

For the development of new IMRB items, the 15 old items were reviewed to uncover content-based perspectives for why those items performed well. Additionally, 86 think-alouds were conducted on the newly selected passages—also obtained from an introductory undergraduate biology textbook—using biology course alumni. Based on the information collected, item specifications were reverse engineered for the development of a new set of 21 items to be field tested and added to the IMRB pool. Of these, 15 new IMRB items were preserved—resulting in a total of 30 IMRB items.

Two parallel 15-item IMRB forms were developed from these 30 items. The validity argument (Fechter et al., 2020) for the IMRB contains additional details that support the validity of this test development process. Additionally, one research study (Cromley et al., 2019) and one research methods manuscript (Dai et al., 2018) provide supporting details regarding the cognitive interviewing approach, importance, and findings.

# 8. Psychometric Form Analysis

This section provides relevant statistical and evaluative information about IMRB test forms—Form A and Form B. These forms were built to be parallel to one another meaning that scores on Form A are interchangeable (or comparable) with scores on Form B.

## Descriptive Statistics

For IMRB Form A and Form B, Table 8-1 provides a summary of distributional estimates for number correct scores. These estimates come from a summary of the Fall 2019 IMRB administration where 116 students from one university responded to Form A test questions and 114 students from the same university responded to Form B test questions.

### Table 8-1. Descriptive Statistics for Form A and Form B

| Form | | ***A*** | **B** |
| --- | --- | --- | --- |
| Number of Examinees | 116 | | 114 |
| Mean Number Correct | 8.28 | | 8.22 |
| Median Number Correct | 9 | | 8 |
| Minimum Number Correct | 1 | | 0 |
| Maximum Number Correct | 15 | | 15 |
| Number Correct Standard Deviation | 3.46 | | 3.13 |

## Reliability

This section provides estimates of reliability based on internal consistency (also known as Cronbach alpha; Cronbach, 1951). Coefficient alpha is a lower-bound estimate of a test’s reliability, and its value depends on how highly items correlate. Coefficient alpha assumes that all items have the same true variance, which is estimated by the average item covariance. These coefficients generally range from 0 to 1, with larger values indicating more internal consistency, and thus providing supporting evidence that the test items are measuring a similar construct. It should be noted that high values of coefficient alpha should not be interpreted to mean that the test is unidimensional (or measuring only one construct), as it is possible to obtain high values of reliability even when multiple dimensions exist. (Note: It is rare, but coefficient alpha can attain negative values.) Equation 1 presents the formula for calculating coefficient alpha.

|  | $\boldsymbol{\alpha=}\frac{\boldsymbol{n}}{\boldsymbol{n-1}}\boldsymbol{(1 -}\frac{\sum_{\boldsymbol{i=1}}^{\boldsymbol{n}} \boldsymbol{\sigma}_{\boldsymbol{Yi}}^{\boldsymbol{2}}}{\boldsymbol{\sigma}_{\boldsymbol{x}}^{\boldsymbol{2}}}\boldsymbol{)}$ | (1) |
| --- | --- | --- |

The variables in this equation consist of 𝑛 items (the number of items appearing on the test form), the index for item (𝑖), which will begin with one and hold a maximum value of 𝑛, the variance ($\sigma_{x}^{2}$) of the total raw scores (*x*) for the test, and the variance ($\sigma_{Yi}^{2}$) of each item (𝑌𝑖). For the IMRB, all items are scored as either incorrect (0) or correct (1). Therefore, $\sigma_{Yi}^{2}$ is estimated by the proportion of correct responses to an item times the proportion of incorrect responses.

The reliability estimates come from the Fall 2019 IMRB administration where 116 students from one university responded to Form A test questions and 114 students from the same university responded to Form B test questions. Test Form A has a Cronbach alpha of 0.75 and Test Form B has a Cronbach alpha of 0.70. These reliability estimates are modest—typically, one would like to see reliability estimates of 0.85 or higher—but considering that the IMRB is used for making low-stake decisions, a reliability of 0.70 or higher is considered adequate.

## Standard error of measurement

This section presents the standard error of measurement (SEM) for each test form. Consider a hypothetical distribution of test scores for a fixed person over repeated measurements with the same test. This distribution is called the propensity distribution (Lord & Novick, 1968); the mean of this distribution is the person’s true score, and the standard deviation is called the SEM. The SEM is an index of the expected variation in the raw scores due to measurement error. The SEM can be used to determine the range of scores for a specified confidence level (e.g., 95%) in which an examinee’s score will fall with repeated testing. The true score is an examinee’s error-free score, and is, of course, unobservable.

Equation 2 provides the algorithm for calculating the SEM.

|  | $\boldsymbol{SEM=}\boldsymbol{\sigma}_{\boldsymbol{x}}\sqrt{\boldsymbol{1-\alpha}}$ | (2) |
| --- | --- | --- |

Two variables make up the SEM calculation, the standard deviation ($\sigma_{x}$) of the total raw scores (*x*), and the reliability estimate, e.g., coefficient alpha (α). As reliability increases, the SEM decreases. The greater the SEM, the more variance in the raw scores can be attributed to poor test design.

The SEM estimates come from the Fall 2019 IMRB administration where 116 students from one university responded to Form A test questions and 114 students from the same university responded to Form B test questions. Test Form A has an SEM of 1.73 and Test Form B has an SEM of 1.71. The following is an example of how to use the SEM to calculate the range of scores that contains an examinee’s scores—with 95% (± 2 SEMs) confidence—upon repeated testing.

For an examinee with a total raw score of 5 on Form A, we can calculate a confidence interval using the SEM of 1.73. For 95% confidence, 1.73 must be multiplied by 2 (= 3.46). The possible score range, over repeated measurements using the same test, is calculated by subtracting 3.46 from 5 and adding 3.46 to 5 (= 1.54 to 8.46)—this means that there is 95% confidence that the interval 1.54 to 8.46 includes the scores of an examinee that would be attained with repeated measurement using the same test.

## Dimensionality Analysis

This section presents the results of confirmatory factor analysis (CFA) of our 30 items. Since the 30 items are designed to measure the deductive reasoning from newly-presented biology information, we expect our test forms are unidimensional, i.e. measuring deductive reasoning only. Our CFA results are consistent with our expectation: the chi-square test is nonsignificant, indicating that our one-factor (unidimensional) CFA model fits our data well. The fit indices, such as CFI, TLI, RMSEA, and SRMR, all meet the cutoff values in Hu and Bentler (1999), suggesting that the unidimensional model fits our data well.

## Test Characteristic Curves, Test Information Functions, and Conditional Standard Error of Measurement

This section relies upon item response theory (IRT) modeling as a vehicle for describing test form difficulty and performance of examinees on the same common scale (i.e., Theta). The Theta scale is fixed to have a mean of 0 and standard deviation of 1 with respect to examinee performance, and ranges from negative infinity to positive infinity with the majority of the scale falling between -4 (very low performance) and 4 (very high performance). This Theta scale can be mapped to the number correct (raw score) scale; this mapping is determined by how difficult items on any given form are for examinees. The extent to which unique test forms produce the same Theta-to-raw-score conversion tables is an indication of how parallel (or similar in difficulty) the test forms are. An analysis of test form equivalence will be provided in the next section. A graphical display of the Theta-to-raw-score mapping is presented in the first pane of Figure 8-1.

With the three IRT-based parameter estimates, the probability (𝑃) of an examinee with any given ability (𝜃) for answering an item correctly can be determined. When these probabilities (for any given ability) are summed across items on a test form, the expected number correct score (𝐸(𝑋)) can be obtained (see Equation 3). Expected number correct scores (raw scores) are mapped across the ability, or Theta, continuum—referred to as a test characteristic curve (TCC) as presented in the first pane of Figure 8-1.

|  | $\boldsymbol{E}\left( \boldsymbol{X} \right)\boldsymbol{=}\sum_{\boldsymbol{i=1}}^{\boldsymbol{n}} \boldsymbol{P}_{\boldsymbol{i}}\boldsymbol{(\theta)}$ | (3) |
| --- | --- | --- |

The second pane displays the test information function (TIF, see Equation 4). Information (𝐼) is a measure of how well a test functions with respect to an ability (𝜃) region of interest. That is, higher values of information are indicative of the ability to more strongly support classification decisions made at the points where higher information is realized along the ability continuum. Likewise, higher values are associated with a smaller standard error of measurement.

|  | $\boldsymbol{I}\left( \boldsymbol{\theta} \right)\boldsymbol{=}\sum_{\boldsymbol{i=1}}^{\boldsymbol{n}} \boldsymbol{a}_{\boldsymbol{i}}^{\boldsymbol{2}}\frac{\left( \boldsymbol{P}_{\boldsymbol{i}}\left( \boldsymbol{\theta} \right)\boldsymbol{-}\boldsymbol{c}_{\boldsymbol{i}} \right)^{\boldsymbol{2}}}{{\boldsymbol{(1-}\boldsymbol{c}_{\boldsymbol{i}}\boldsymbol{)}}^{\boldsymbol{2}}}\frac{\boldsymbol{(1-}\boldsymbol{P}_{\boldsymbol{i}}\left( \boldsymbol{\theta} \right)\boldsymbol{)}}{\boldsymbol{P}_{\boldsymbol{i}}\left( \boldsymbol{\theta} \right)}$ | (4) |
| --- | --- | --- |

The final pane displays the conditional standard error of measurement (CSEM, see Equation 5). The CSEM is the reciprocal of the square root of the TIF. Smaller values are indicative of less error in the examinee ability estimates. Larger values are indicative of more error in the examinee ability estimates, and thus are less trustworthy classification decisions for examinees. Assessments designed well will have information highest (and CSEM lowest) surrounding the cut points (the location where classification decisions are made).

|  | $\boldsymbol{CSEM}\left( \boldsymbol{\theta} \right)\boldsymbol{=}\frac{\boldsymbol{1}}{\sqrt{\boldsymbol{I(\theta)}}}$ | (5) |
| --- | --- | --- |

A typical rule of thumb is that a CSEM of 0.3 or less is considered reasonable. This is approximately equivalent to 𝐼(𝜃) = 10, or a conditional reliability (CREL) 0.9. Therefore, it is desirable to realize values of 10 or higher for information surrounding the cut points. This is practically impossible for a 15-item test. Thus, these thresholds serve as guidance and not as rules.


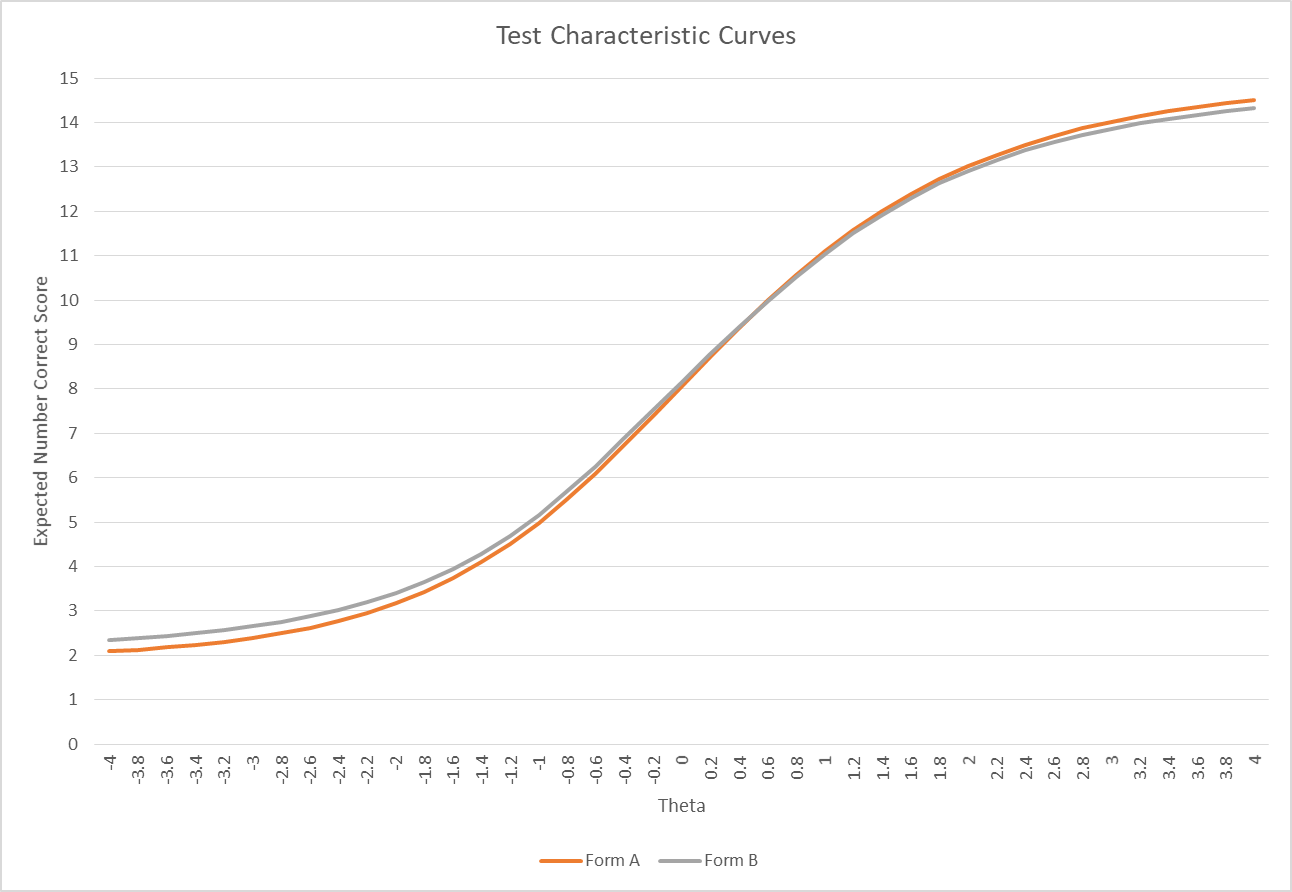


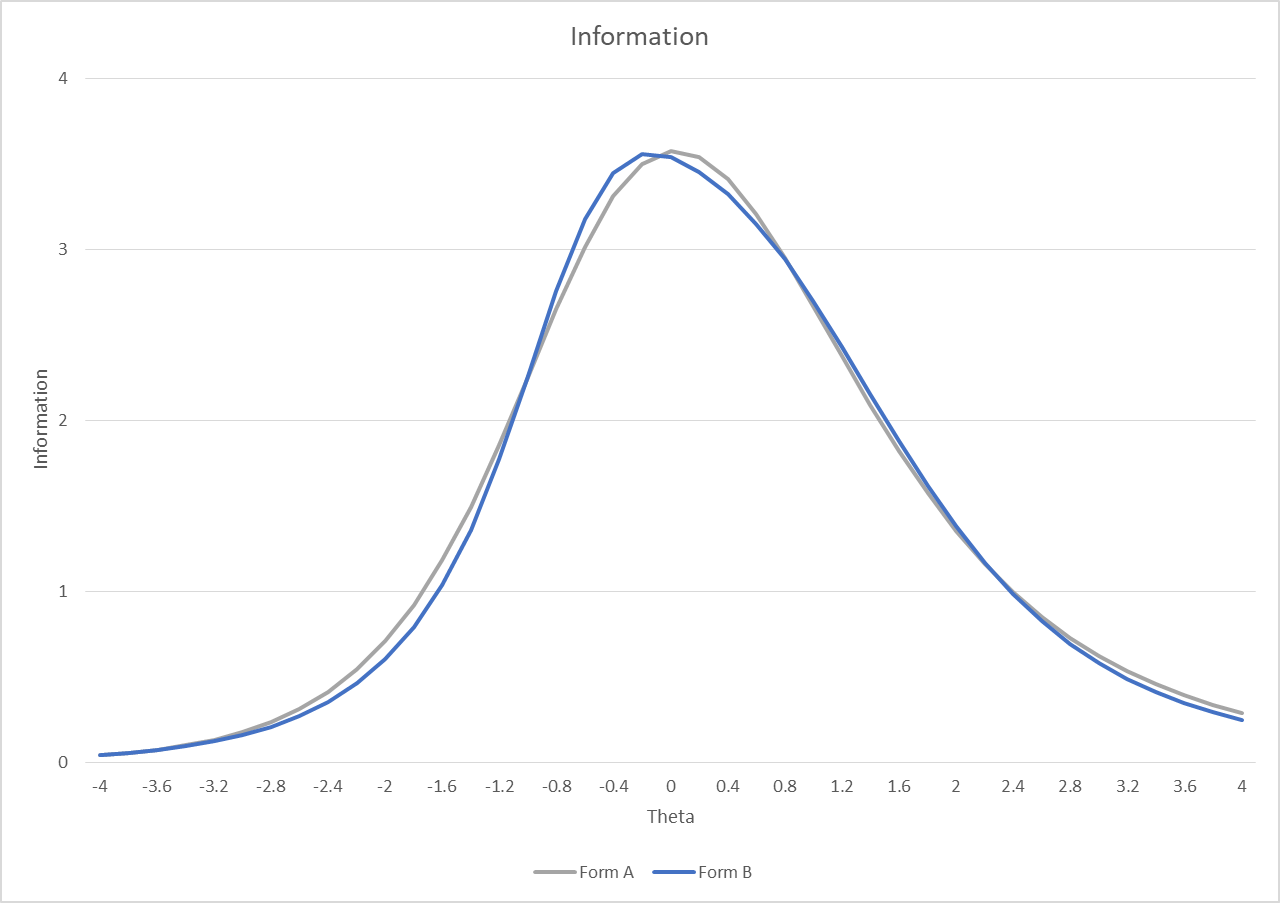


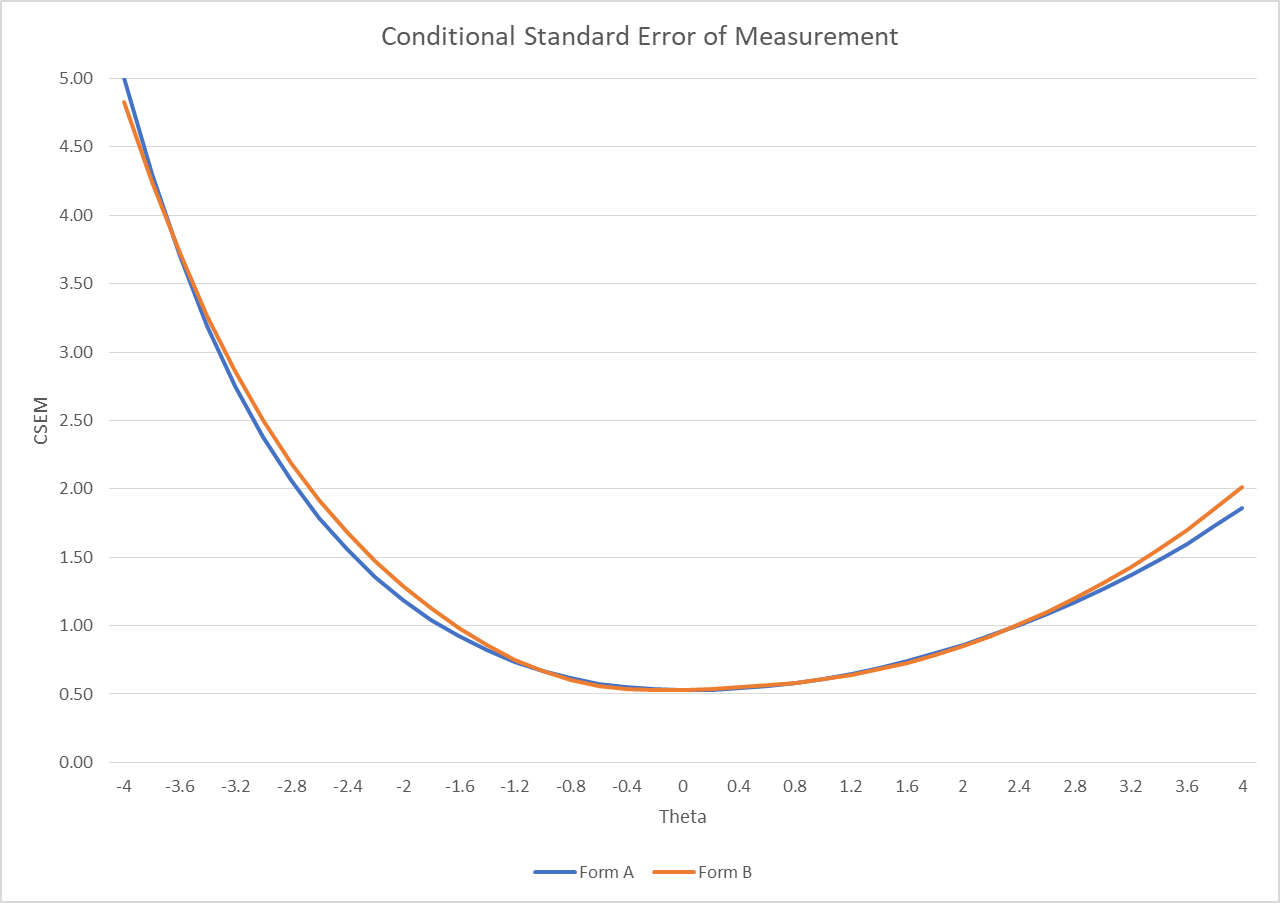


### Figure 8-1. IMRB Form A and Form B Test Characteristic Curves, Information, and Conditional Standard Error of Measurement

## Form Equivalence and Quality

Table 8-2, which summarizes the classical psychometric characteristics of the IMRB test forms, taken together with the item response theory (IRT) characteristics presented in Figure 8-1 provide ample evidence that the two test forms are comparable. That is, Form A and Form B express very similar classical and IRT properties. Observing the panes of Figure 8-1 support that for all ability groups, the two test forms are interchangeable—that is, the curves are all significantly overlapping.

As observed in Figure 8-1—the second pane—the IMRB test forms are most informative between -1.0 and +1.2 theta which by looking at the first pane (or calculating), translates to a range of 5 to 12 number correct score points. Thus, scores between these two values will be the most precise and will be most useful for making decisions about examinees.

## Table 8-2. Form A and Form B Psychometric Characteristics

| Form | | ***A*** | **B** |
| --- | --- | --- | --- |
| **Number of Examinees** | 116 | | 114 |
| **Mean Number Correct** | 8.28 | | 8.22 |
| **Median Number Correct** | 9 | | 8 |
| **Minimum Number Correct** | 1 | | 0 |
| **Maximum Number Correct** | 15 | | 15 |
| **Number Correct Standard Deviation** | 3.46 | | 3.13 |
| **Cronbach Alpha (Reliability)** | 0.75 | | 0.70 |
| **Standard Error of Measurement** | 1.73 | | 1.71 |

# 9. Psychometric Item Analysis

This section provides relevant statistical and evaluative information about individual items contained within the IMRB. There are two developed test forms. Information describing each item will be provided for all items on Form A and then for all items on Form B. As described in the last section, these forms were built to be parallel to one another meaning that scores on Form A are interchangeable (or comparable) with scores on Form B. Items are presented in the order they appear on each form.

Information presented includes the *p*-value, point biserial correlation (ptbsrl), factor loading, IRT parameter estimates (i.e., difficulty, discrimination, and likelihood of correct guessing), and differential item functioning evaluation. Each of these analyses are described next, followed by a table or graphics that provide the results for items on the IMRB.

## *p*-value

The *p*-value, or classical item difficulty, is defined as the average proportion of examinees who answer the item correctly. Items that are answered correctly by almost all examinees provide little information about differences in the examinees tested since nearly all examinees were capable of providing a correct response. Similarly, items that are answered incorrectly by almost all examinees also provide little information about differences in the examinees tested since nearly all examinees were incapable of providing a correct response. However, when the focus of an assessment is on mastery of content or a specified skill (i.e., reasoning)—and not on comparing the relative performance of examinees to one another—it is not unreasonable that some items may be very easy or very hard.

In general, to provide the best measurement, p-values should have a range from near-chance performance (0.25 for four-option multiple-choice items) to 0.90, with the majority of items generally falling between 0.40 and 0.70.

Table 9-1 provides item summary statistics for each item. *p*-values come from the field test conducted in Fall 2017 with 1,435 examinees.

## *Point Biserial Correlation*

The point biserial correlation is the correlation between performance on a single item with the total test score. This provides an indication as to whether the item is capable of discriminating the higher-ability examinees from the lower-ability examinees. The Pearson product-moment correlation (i.e., point biserial correlation) is used to calculate classical item discrimination and can range from -1.0 to 1.0. Typically, values of 0.20 to 0.60 are considered desirable.

Table 9-1 provides the point biserial correlations from the field test conducted in Fall 2017 with 1,435 examinees.

## *Factor Loadings*

Factor loadings for items are derived following a confirmatory factor analysis (CFA). For the IMRB, one factor is extracted as it is a unidimensional assessment of inference-making and reasoning. The factor loadings represent the strength of the relationship (or correlation) that the item has with the latent construct of interest—deductive reasoning with new information. Factor loadings for the IMRB were computed using the January 2019 examinee data from UIUC (*N* = 201; presented in Table 9-1). The model fit was excellent (Hu & Bentler, 1999), with a chi-square of 475.99 (*p* = .0085), CFI = .961, and RMSEA = .030 [CI90%: .016, .040], indicating the 1-factor, 30-item model fits the data adequately. Standardized factor loadings greater than .30 are considered to be acceptable.

## *IRT Parameter Estimates*

Item Response Theory (IRT) modeling is the process of calibrating items (estimating item parameter values) by fitting a statistical model to examinee item response data. IRT statistics were estimated using version 2.0.5.3 of the flexMIRT computer program (Cai, 2013). Each test form’s items were calibrated concurrently, meaning that all items are placed on the same ability scale. Examinee ability is denoted as theta (𝜃) and is scaled to have a mean of 0 and a standard deviation of 1 with respect to the calibration sample. The three-parameter logistic model (3PL) was chosen to calibrate item statistics. Three parameters, difficulty (𝑏), discrimination (𝑎), and pseudo-guessing (c), are estimated for each item (𝑖). Equation 6 introduces the 3PL model.

|  | $\boldsymbol{P}_{\boldsymbol{i}}\boldsymbol{(\theta)=}\boldsymbol{c}_{\boldsymbol{i}}\boldsymbol{+(1-}\boldsymbol{c}_{\boldsymbol{i}}\boldsymbol{)}\frac{\boldsymbol{1}}{\boldsymbol{1+exp(-}\boldsymbol{a}_{\boldsymbol{i}}\boldsymbol{(\theta-}\boldsymbol{b}_{\boldsymbol{i}}\boldsymbol{))}}$ | (6) |
| --- | --- | --- |

Item difficulty is represented by a location parameter. The location parameter is generally designated by the letter, *b*, as in the “*b*-value.” Lower values indicate easier items with respect to examinee ability, and higher values indicate harder items with respect to examinee ability. Values typically range from -4 to 4, and can take on values between positive and negative infinity.

Item discrimination is designated by the letter *a*, which is commonly called the “*a*-value”. The *a*-value refers to the slope of the item characteristic curve. Lower values indicate that the item performs poorly for distinguishing between low- and high-performing examinees. Higher values indicate that the item performs well for distinguishing between low- and high-performing examinees. Values typically range from 0.0 to 3.5.

The pseudo-guessing parameter (*c*-value) provides an estimate for the propensity of examinees to correctly respond to a given item by chance alone (i.e., guess the correct response). Higher values indicate a greater propensity for guessing correctly the answer to the given item, and lower values, a lower propensity. Values typically hover around 0.20 for items with four response options.

Table 9-1 provides the number of examinees estimates are based, item parameter estimates, and their associated standard errors. Generally, standard errors (SE) above 0.40 for a- and b-values are considered unacceptable, and use of an item with such a value should be reconsidered. For c-values, the threshold for SE is 0.05. Parameters for IMRB items are calibrated using 3,969 examinees over several IMRB test administrations and various form configurations. Thus, each item is calibrated with a varying number of examinees. Fifteen items were originally created for the IMRB and thus have significantly more data associated with them. The other fifteen items were trialed during the field test and have been matrixed across forms and thus have fewer data associated. Standard errors for the parameter estimates of these items are expected to be higher as a result. Therefore, the general guidelines for interpreting standard errors does not apply for the new 15 items. New items have less than 1,000 examinees. Examinees choosing to skip an item were not included in the calibration for that item. That is, instead of the response being scored as incorrect, it was coded as not presented.

## *Differential Item Functioning*

Differential item functioning (DIF) is a phrase used to identify the potential presence of item bias, where an item favors one subgroup (e.g., males) over another (e.g., females). The cause of the favoritism cannot be attributed to a real difference in ability on the construct of interest (e.g., reasoning) because DIF studies first match examinees in each subgroup based on overall performance on the assessment. Overall performance is based on either total score on a set of anchor items—items known to not display DIF between the subgroups—or on the total score on the assessment except for the one item being analyzed for DIF. Generally, there is a referent and a focal group and the focal group is considered the more vulnerable subgroup (e.g., females) based on historical observations and outcomes.

Three separate DIF analyses were conducted for the IMRB:

1. Sex: Referent Group = Males; Focal Group = Females,
2. Race: Referent Group = Whites; Focal Group = Non-Whites, and
3. College Generation: Referent Group = 2^nd^ Generation College+; Focal Group = 1^st^ Generation College.

Examinees were identified with subgroups based on how they responded to demographic information asked following the IMRB administration. 1^st^ generation college students are identified as any student who does not have a parent (mother or father) with an education level of obtaining a Bachelor’s degree or higher. Everyone else is considered 2^nd^ generation or more.

The first 15 IMRB test items underwent a DIF analysis using data from 1,665 examinees tested between 2009 and 2015. Items flagged for DIF all had effect sizes that indicated negligible DIF was present. That is, based on the Mantel-Haenszel (MH) DIF detection method (1959) and Educational Testing Service (ETS) derived effect size interpretations (Dorans & Holland, 1993; Zieky, 1993), none of the items presented with significant DIF. Negligible DIF (present, but unlikely to be practically significant) was detected for four items based on sex, one item based on race, and one item based on college generation. All items were unique (no item was flagged more than once). Respective to their comparison group, these items were removed from the calculation of the total score for the purpose of matching groups for future DIF detection studies. Thus, the remaining items of the 15 for each comparison is considered the anchor set for detecting DIF.

The matrixed item response patterns from various IMRB test administrations (consisting of 3,969 examinees) were used to detect DIF for the newly created items and the items flagged for DIF were negligible, according to their effect sizes. Given the larger sample size, an IRT-based method for DIF detection was employed. The steps for this method are presented.

1. Calibrate item parameters using a 3-parameter IRT model
   1. Constrain the model so that the anchor items are fixed to have the same values between subgroups
   2. Separately calibrate the remaining items between subgroups
2. Test for parameter estimate differences for the separately calibrated items between subgroups using the improved Wald test (Woods, Cai, & Wang, 2012)

Results indicate no items were flagged for DIF in the three subgroup comparisons of interest. The exception is that within the sex comparison, one item (new_7) did not reach convergence for its calibration for the female subgroup, and thus, DIF was detected, but not interpretable due to poor calibration. This result is noted in Table 9-1.

## Table 9-1. IMRB Item Statistics

|  |  |  |  | | IRT Parameter Estimates | | | | | | |  |
| --- | --- | --- | --- | --- | --- | --- | --- | --- | --- | --- | --- | --- |
| Item | ***p*-value** | **ptbsrl** | **Factor**  **Loading** | **N** | | **Discrimination** | ***SE*** | **Difficulty** | ***SE*** | **Guessing** | ***SE*** | ***DIF*** |
| new_2 | 0.47 | 0.37 | 0.65 | 748 | | 1.48 | *0.32* | 0.41 | *0.17* | 0.13 | *0.07* |  |
| new_3 | 0.66 | 0.40 | 0.69 | 655 | | 0.98 | *0.19* | -0.20 | *0.32* | 0.10 | *0.11* |  |
| new_4 | 0.51 | 0.32 | 0.54 | 612 | | 0.91 | *0.30* | 0.89 | *0.32* | 0.18 | *0.09* |  |
| new_5 | 0.41 | 0.20 | 0.30 | 196 | | 0.30 | *0.24* | 2.76 | *2.19* | 0.15 | *0.14* |  |
| new_6 | 0.41 | 0.42 | 0.64 | 651 | | 0.99 | *0.27* | 0.99 | *0.22* | 0.09 | *0.07* |  |
| new_7 | 0.60 | 0.27 | 0.70 | 634 | | 1.48 | *0.25* | -0.09 | *0.17* | 0.07 | *0.08* | *** |
| new_9 | 0.62 | 0.37 | 0.61 | 768 | | 1.30 | *0.30* | 0.09 | *0.27* | 0.18 | *0.10* |  |
| new_10 | 0.39 | 0.23 | 0.43 | 795 | | 0.59 | *0.15* | 0.72 | *0.44* | 0.10 | *0.10* |  |
| new_11 | 0.54 | 0.37 | 0.74 | 802 | | 0.66 | *0.14* | 0.04 | *0.41* | 0.08 | *0.11* |  |
| new_12 | 0.37 | 0.22 | 0.31 | 597 | | 0.77 | *0.51* | 2.11 | *0.64* | 0.23 | *0.09* |  |
| new_13 | 0.38 | 0.37 | 0.45 | 747 | | 0.76 | *0.21* | 0.90 | *0.33* | 0.09 | *0.10* |  |
| new_14 | 0.76 | 0.32 | 0.64 | 599 | | 1.24 | *0.28* | -0.61 | *0.39* | 0.22 | *0.14* |  |
| new_15 | 0.51 | 0.44 | 0.56 | 855 | | 1.07 | *0.18* | 0.16 | *0.22* | 0.07 | *0.08* |  |
| new_18 | 0.17 | 0.29 | 0.67 | 795 | | 0.69 | *0.14* | 0.24 | *0.36* | 0.07 | *0.10* |  |
| new_21 | 0.64 | 0.44 | 0.74 | 795 | | 1.15 | *0.18* | -0.22 | *0.23* | 0.07 | *0.09* |  |
| old_1 | 0.37 | 0.38 | -0.04 | 3,510 | | 0.75 | *0.16* | 1.27 | *0.19* | 0.07 | *0.07* |  |
| old_2 | 0.77 | 0.35 | 0.24 | 3,455 | | 1.18 | *0.16* | -0.36 | *0.23* | 0.17 | *0.09* |  |
| old_3 | 0.71 | 0.49 | 0.67 | 3,753 | | 1.82 | *0.16* | -0.39 | *0.10* | 0.09 | *0.05* |  |
| old_4 | 0.85 | 0.46 | 0.77 | 3,456 | | 2.42 | *0.28* | -0.59 | *0.11* | 0.24 | *0.06* |  |
| old_5 | 0.58 | 0.47 | 0.45 | 3,698 | | 1.52 | *0.20* | 0.46 | *0.09* | 0.22 | *0.04* |  |
| old_6 | 0.67 | 0.35 | 0.73 | 3,649 | | 0.98 | *0.09* | -0.50 | *0.21* | 0.04 | *0.08* |  |
| old_7 | 0.55 | 0.43 | 0.58 | 3,452 | | 1.25 | *0.17* | 0.03 | *0.16* | 0.16 | *0.07* |  |
| old_8 | 0.73 | 0.42 | 0.59 | 3,750 | | 1.22 | *0.13* | 0.10 | *0.11* | 0.05 | *0.05* |  |
| old_9 | 0.66 | 0.45 | 0.79 | 3,495 | | 1.20 | *0.12* | -0.67 | *0.20* | 0.07 | *0.08* |  |
| old_10 | 0.41 | 0.47 | 0.63 | 3,514 | | 1.42 | *0.19* | 0.86 | *0.07* | 0.16 | *0.03* |  |
| old_11 | 0.77 | 0.45 | 0.62 | 3,675 | | 1.05 | *0.11* | -0.82 | *0.28* | 0.08 | *0.11* |  |
| old_12 | 0.46 | 0.48 | 0.70 | 3,547 | | 1.34 | *0.19* | 0.33 | *0.13* | 0.20 | *0.05* |  |
| old_13 | 0.53 | 0.44 | 0.59 | 3,418 | | 0.84 | *0.11* | 0.20 | *0.23* | 0.06 | *0.07* |  |
| old_14 | 0.76 | 0.48 | 0.94 | 3,404 | | 1.62 | *0.22* | -0.62 | *0.20* | 0.22 | *0.10* |  |
| old_15 | 0.51 | 0.43 | 0.72 | 3,473 | | 1.36 | *0.21* | 0.60 | *0.11* | 0.24 | *0.04* |  |

* Note that in conducting the IRT-based DIF analysis for item new_7 for the Female/Male comparison, new_7 produced inconsistent results for the Female group, resulting in the inability to reach convergence. Therefore, the hypothesis test for the presence of DIF could not be determined for this subgroup comparison for this item. Otherwise, no DIF was detected for the subgroups analyzed across all items.

# 10. Future IMRB Development

Given the utility of the IMRB, future development efforts could include generating new and comparable test forms by expanding the existing item bank. Additionally, new test forms could be developed to focus on different content areas also not taught in most high schools. Likewise, the techniques we used (e.g., use of cognitive labs) can be replicated to create other tests of inference-making and reasoning for subject areas beyond biology (e.g., chemistry).

## A Note on Missing Data

The Monte Carlo simulation study (Dai et al., 2019 April) validated a multigroup CFA approach to overcoming the issue of zero covariances between items administered to different respondent groups (or a simple-matrix-sampling PM design). The large majority of models successfully converged and yielded the estimated variance-covariance matrices, which supports Cudeck (2000) in that the zero-covariance issue by the simple-matrix-sampling designs can be resolved. Contrary to popular opinions in the field (Graham et al., 2006; Rhemtulla & Hancock, 2016), our findings indicate that given proper conditions it is possible to conduct analyses that require covariances on variables obtained from a simple-matrix-sampling PM design: instead of relying on the observed variance-covariance matrix with zero covariances among variables not jointly observed, one can estimate variance-covariance matrix by the multigroup CFA model. Using a simple-matrix-sampling PM design no longer prevents researchers from estimating correlations or covariances and related statistics.

Our findings suggest critical elements with a planned missing design that determine statistical power to obtain adequate CFA model fit and those determine parameter estimation (hence the estimated variance-covariance matrix). Small sample sizes (e.g. 500 or 250), low r (e.g., .10 rather than .25), and small k (e.g., 2 rather than 4 anchor items) contribute to inadequate statistical power and inaccurate parameter estimation. Our findings also support the trade-off between sample size and anchor item (i.e., test length), which provides assistance to researchers who design simple-matrix sampling. Our findings further promote the use of simple-matrix-sampling designs. Given its high cost-efficiency, the multigroup modeling approach to estimating variance-covariance matrices, and our cautions about N, r, and k, the simple-matrix-sampling PM designs can be more widely employed in educational research. However, we note that an a priori power estimation with the intended simple-matrix-sampling PM design is most strongly recommended, or else one may be faced with poor model fit (particularly SRMR > .08) and inaccurate estimation of factor loadings and residual variances.

# References

American Educational Research Association (AERA), American Psychological Association (APA), & National Council on Measurement in Education (NCME). (2014)*. Standards for educational and psychological testing*. Washington, DC: American Psychological Association.

Cai, L. (2013). *flexMIRT*® *version 2: Flexible multilevel multidimensional item analysis and test scoring* [Computer software]. Chapel Hill, NC: Vector Psychometric Group.

Cromley, J. G., Dai, T., Fechter, T., Van Boekel, M., Nelson, F. E., & Dane, N. (2019). What cognitive interviewing reveals about a new measure of undergraduate biology reasoning. Revision requested by *Journal of Experimental Education* on April 2, 2019.

Cromley, J. G., Fechter, T., Van Boekel, M., Dai, T., Nelson, F., & Parpucu, A. N. (2018, January). *Think-alouds provide supporting validity evidence: Expected cognitive processes are measured.* Poster presented at the Institute of Educational Sciences Annual Principal Investigators Meeting, Arlington, VA.

Cromley, J. G., Snyder-Hogan, L. E., & Luciw-Dubas, U. A. (2010). Cognitive activities in complex science text and diagrams. *Contemporary Educational Psychology, 35*, 59–74.

Cronbach, L. J. (1951). Coefficient alpha and the internal structure of tests. *Psychometrika, 16*(3), 297–334.

Dai, T., Boekel, M., Cromley, J., Nelson, F. & Fechter, T, (2018). Using think-alouds to create a better measure of biology reasoning. SAGE Research Methods Cases. 10.4135/9781526437167

Dai, T., & Cromley, J. G. (2014). Changes in implicit theories of ability in biology and dropout from STEM majors: A latent growth curve approach. *Contemporary Educational Psychology, 39*(3), 233–247.

Dai, T., Du, Y., Cromley, J.G., Fechter, T., Nelson, F.E. (2019, April). A multigroup factor analysis approach to analyzing simple-matrix-sampling planned missing data: (When) does it work? Paper presented at the annual conference of the American Educational Research Association, Toronto, Canada, April 5-9, 2019.

Dorans, N. J., & Holland, P. W. (1993). DIF detection and description. In P. W. Holland & H. Wainer (Eds.), *Differential item functioning* (pp. 35–66). Hillsdale, NJ: Lawrence Erlbaum.

Hu, L. T., & Bentler, P. M. (1999). Cutoff criteria for fit indexes in covariance structure analysis: Conventional criteria versus new alternatives. *Structural equation modeling: a multidisciplinary journal*, *6*(1), 1-55.

Kane, M. T. (2013). Validating the interpretations and uses of test scores. *Journal of Educational Measurement, 50*, 1–73.

Lord, F. M., & Novick, M. R. (1968). *Statistical theories of mental test scores*. Reading, MA: Addison-Wesley.

Mantel, N., & Haenszel, W. (1959). Statistical aspects of the analysis of data from retrospective studies of disease*. Journal of the National Cancer Institute*, *22,* 719–748.

National Science Foundation. (2006). *Women, minorities, and persons with disabilities in science and engineering.* Available at <https://www.nsf.gov/statistics/2017/nsf17310/>.

Woods, C. M., Cai, L., & Wang, M. (2012). The Langer-improved Wald test for DIF testing with multiple groups: Evaluation and comparison to two-group IRT. *Educational and Psychological Measurement, 73*(3), 532-547.

Zieky, M. (1993). Practical questions in the use of DIF statistics in test development. In P. W. Holland & H. Wainer (Eds.), *Differential item functioning* (pp. 337–347). Hillsdale, NJ: Erlbaum.

Appendix 2:
IMRB Validity Framework Documentation


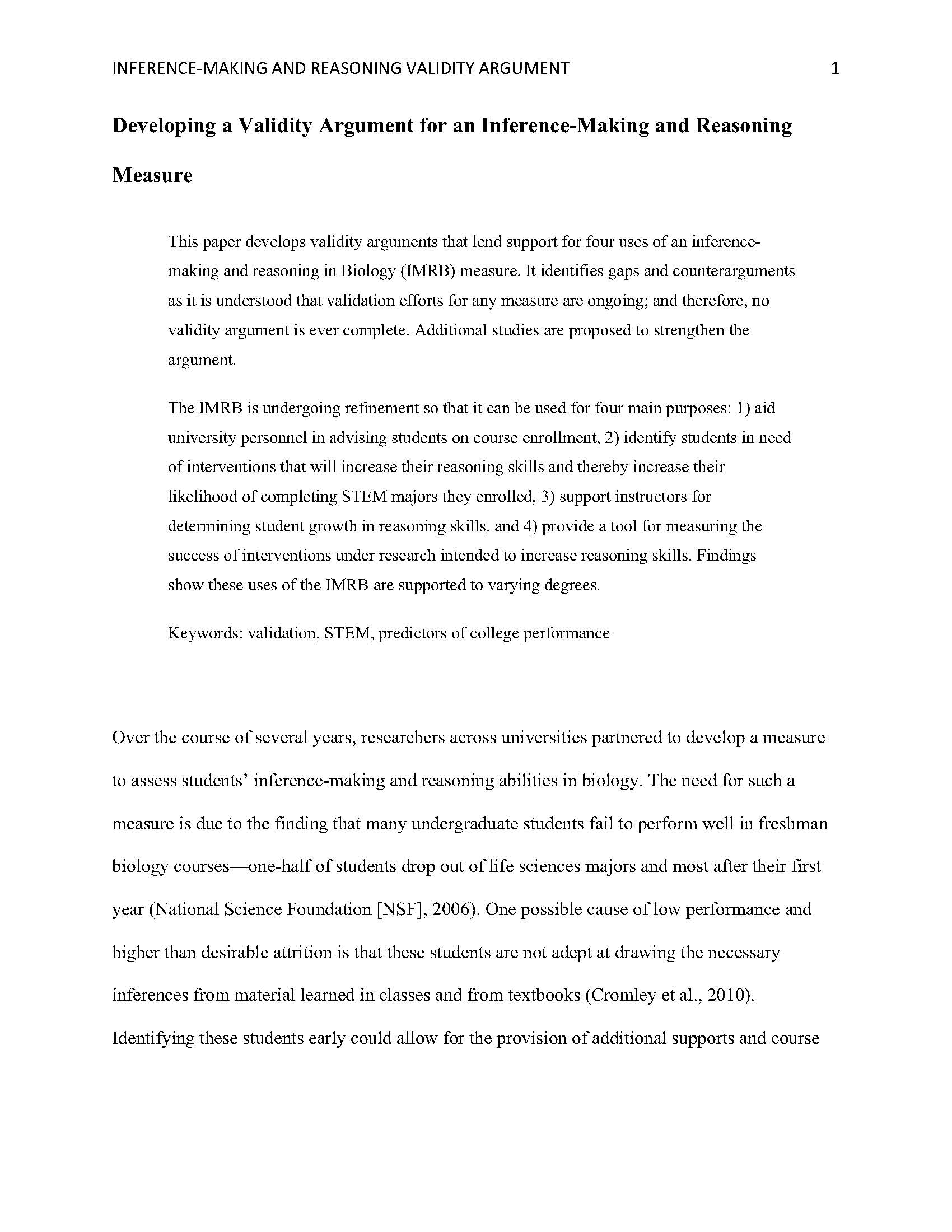


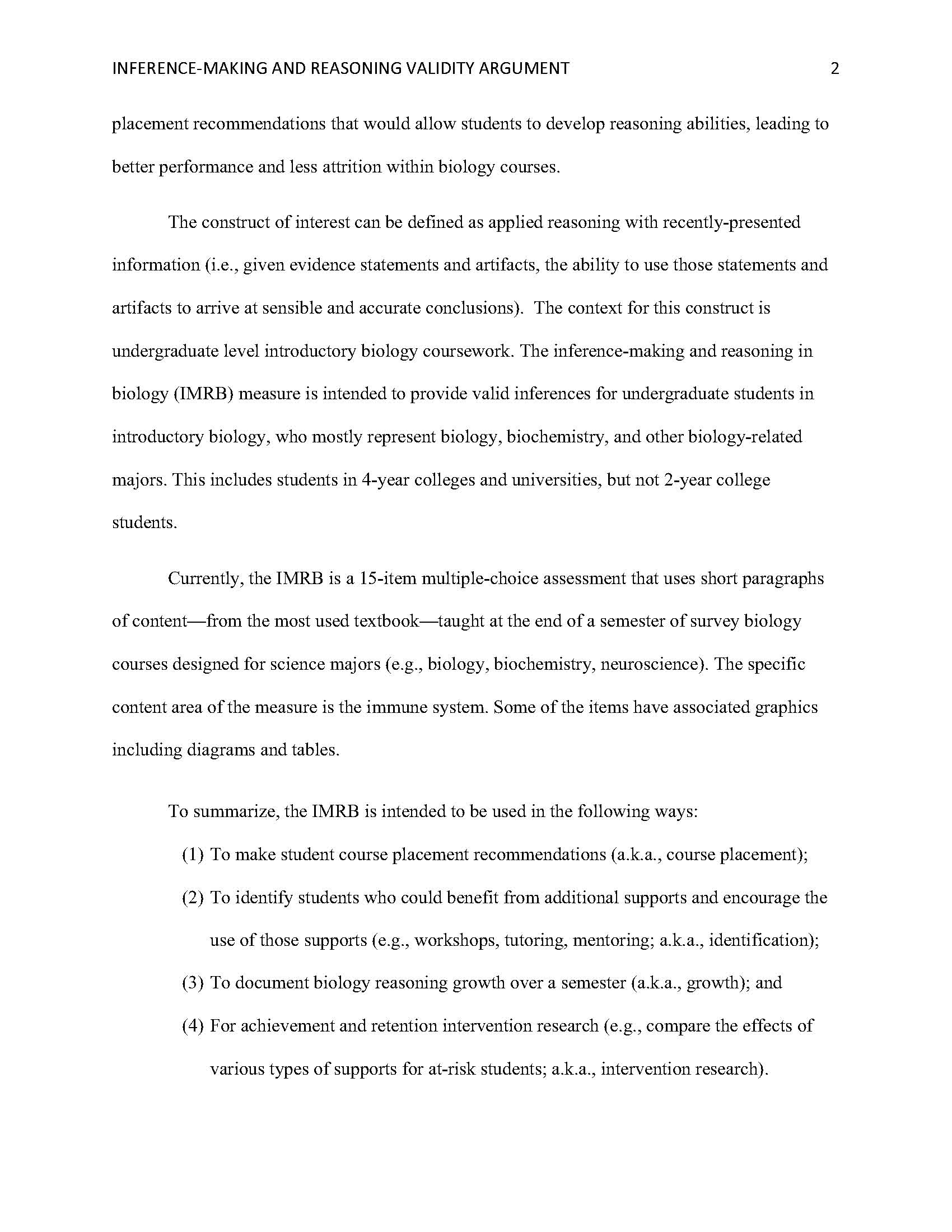


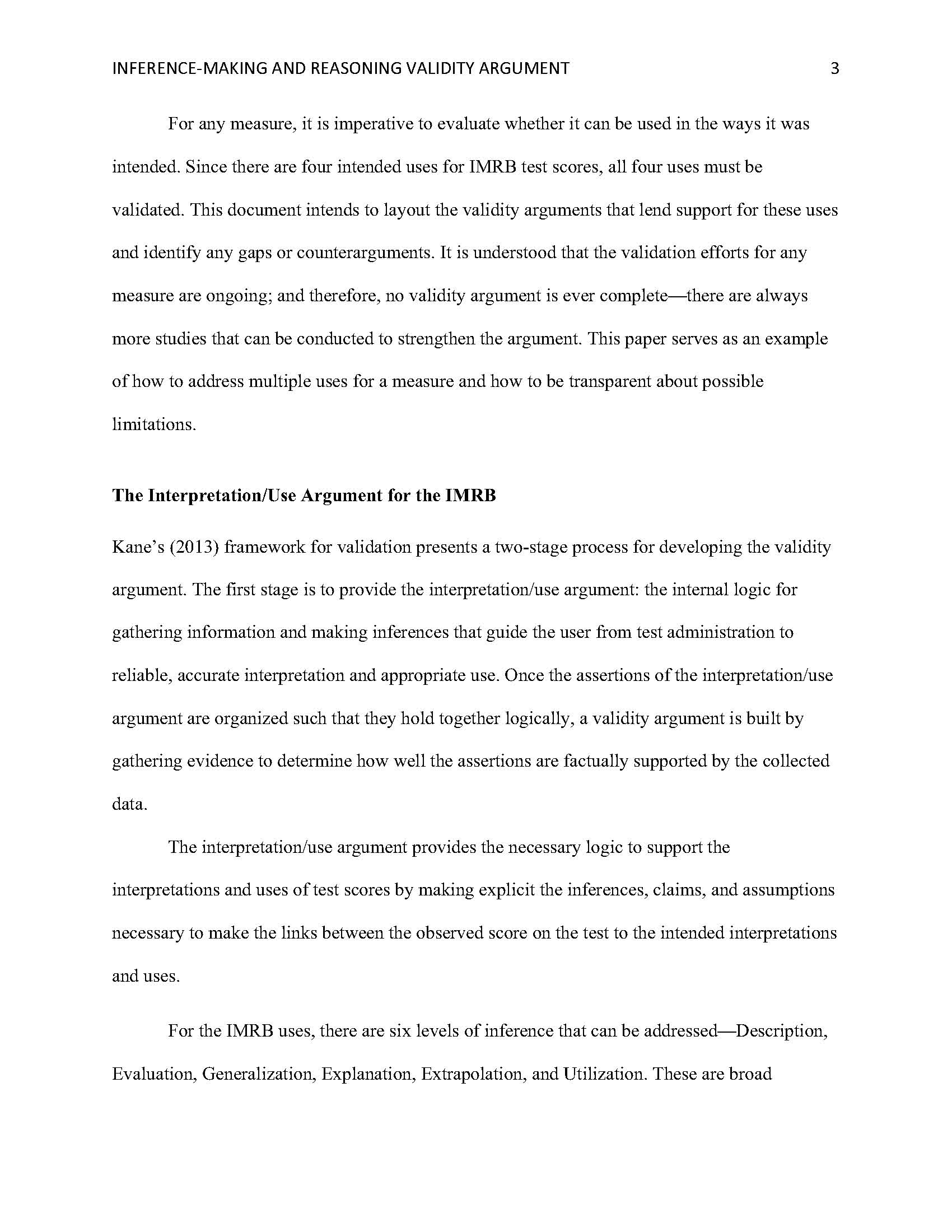

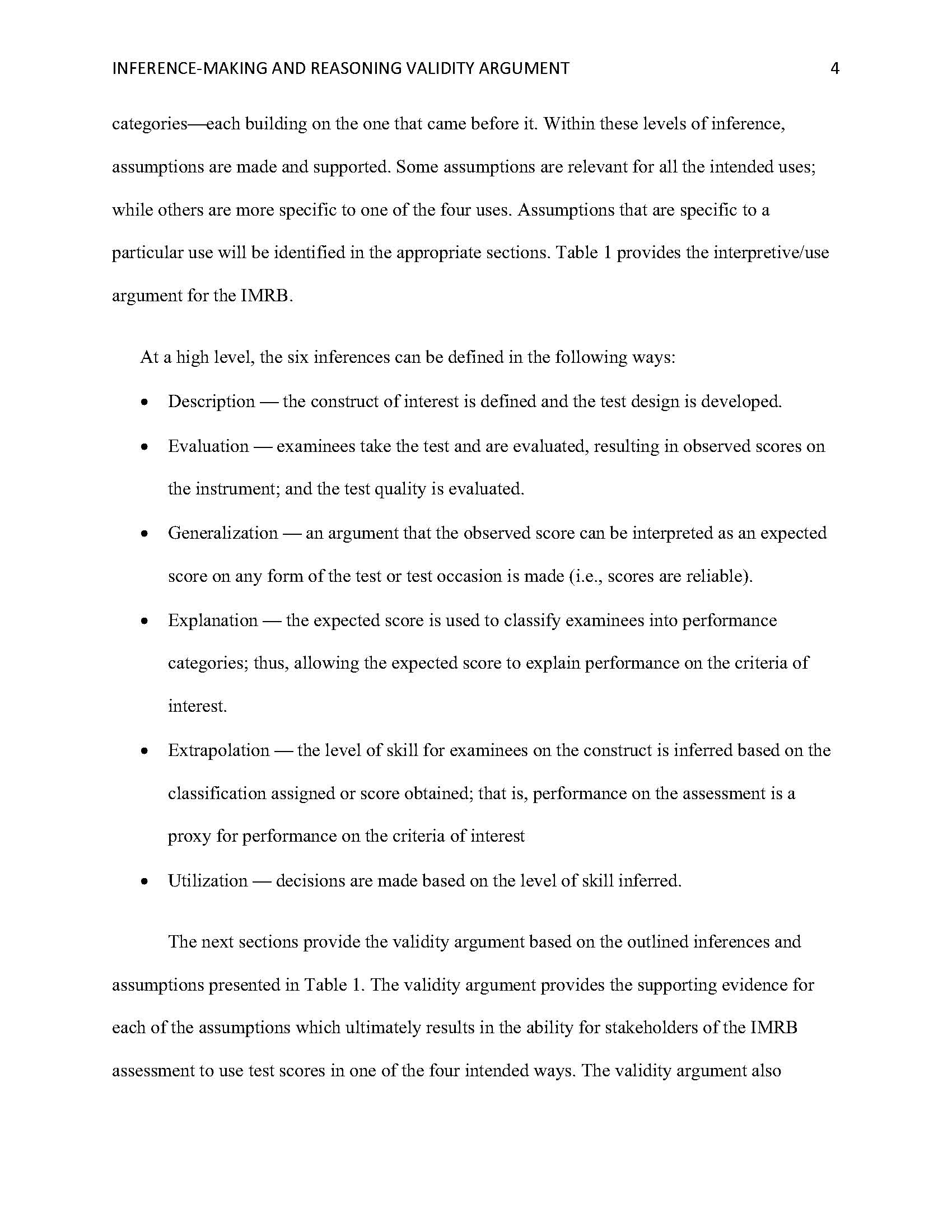


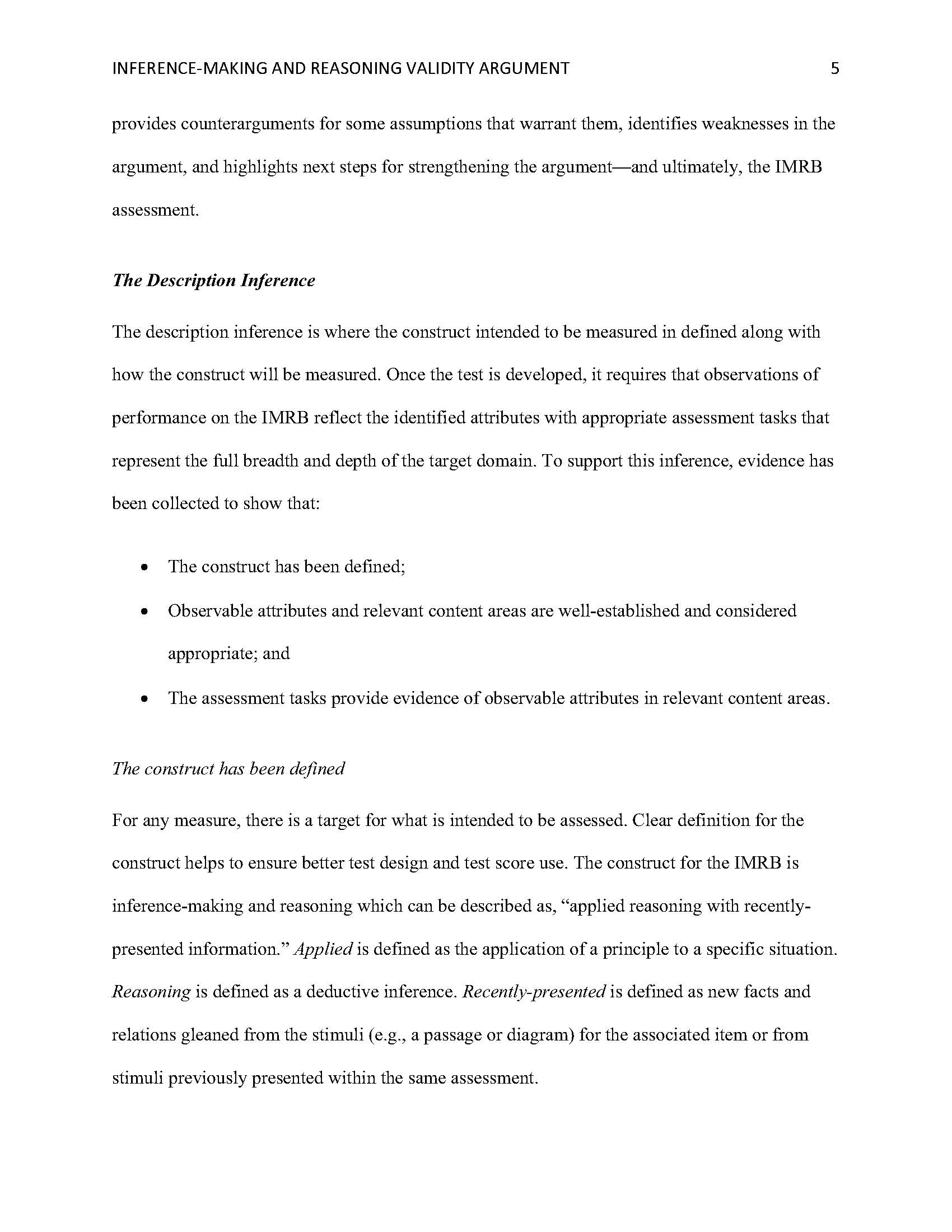


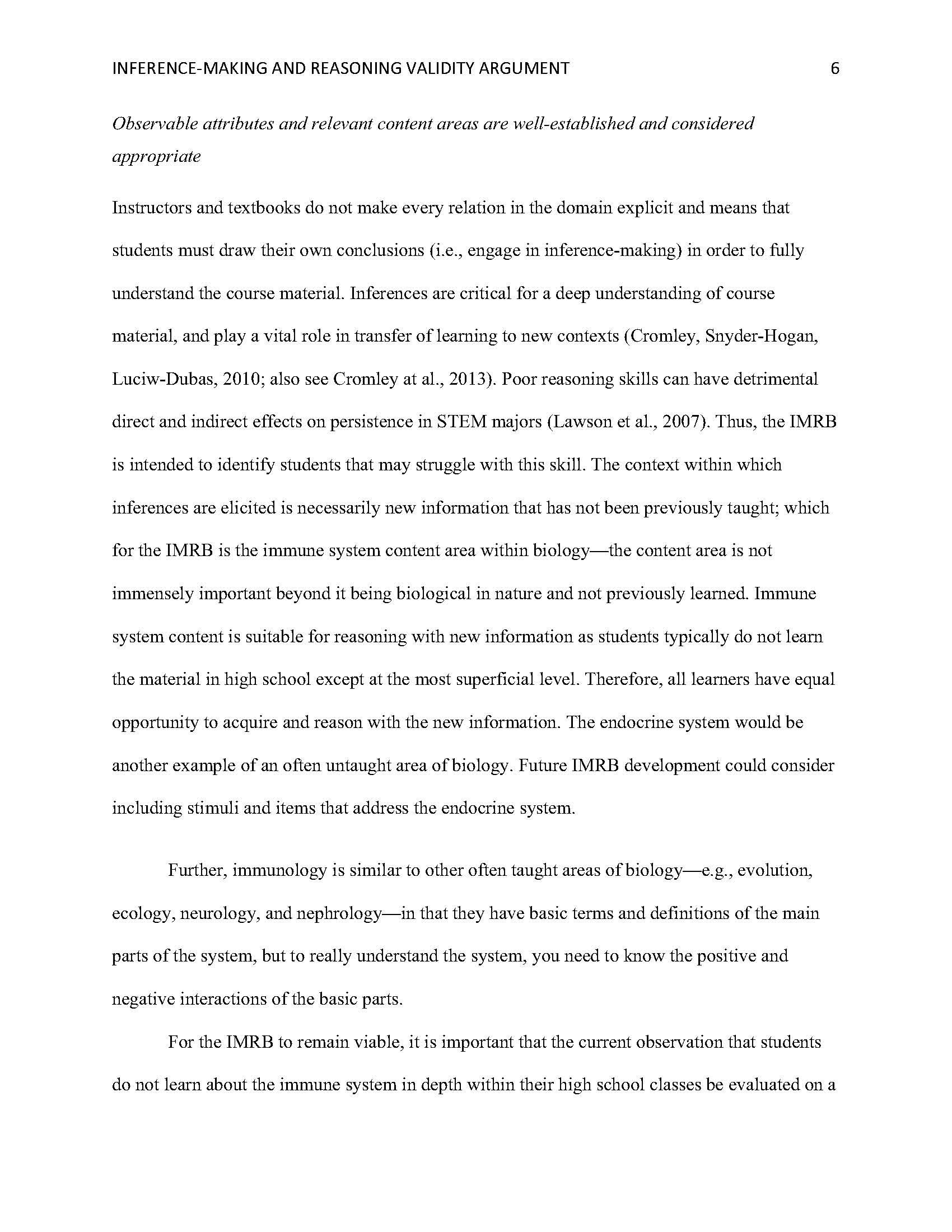


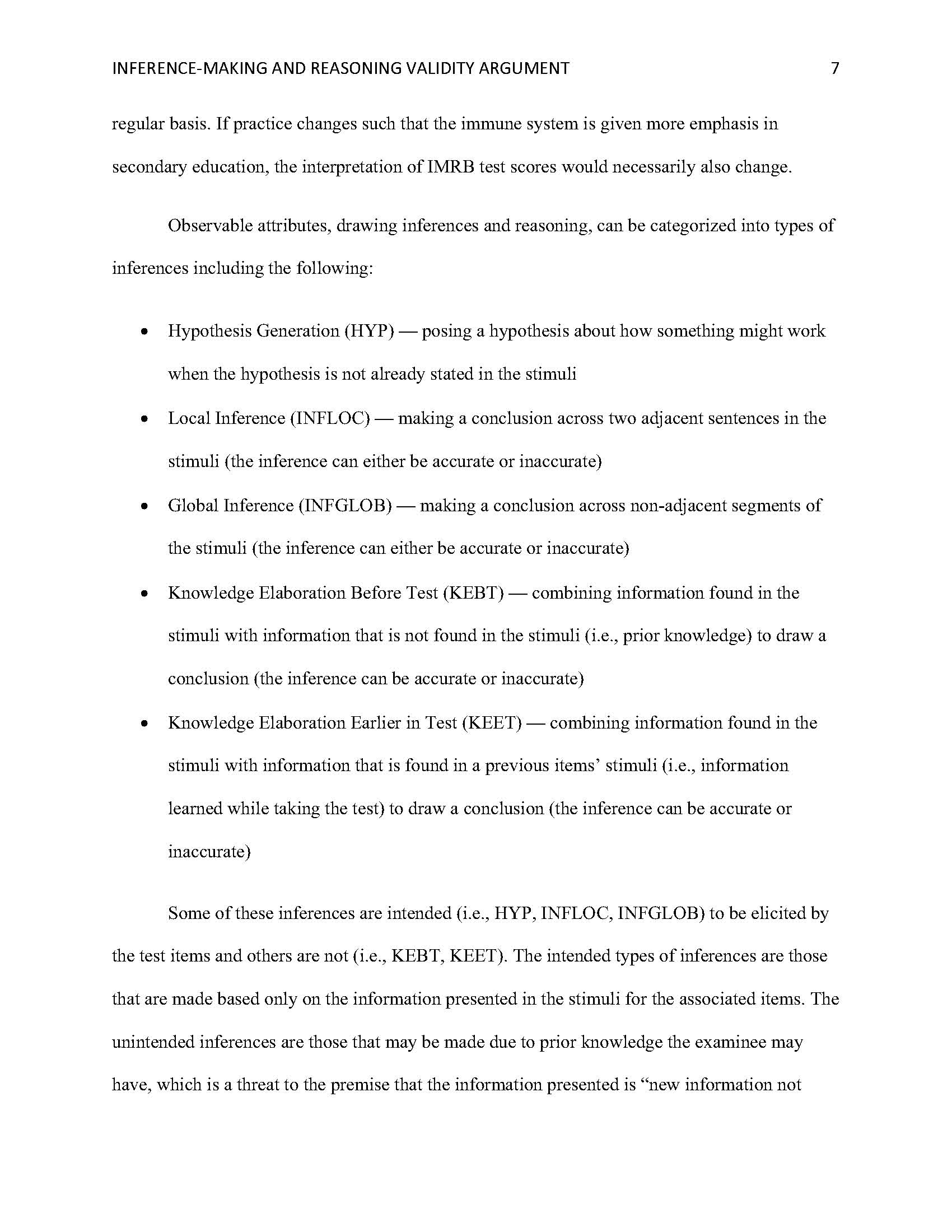

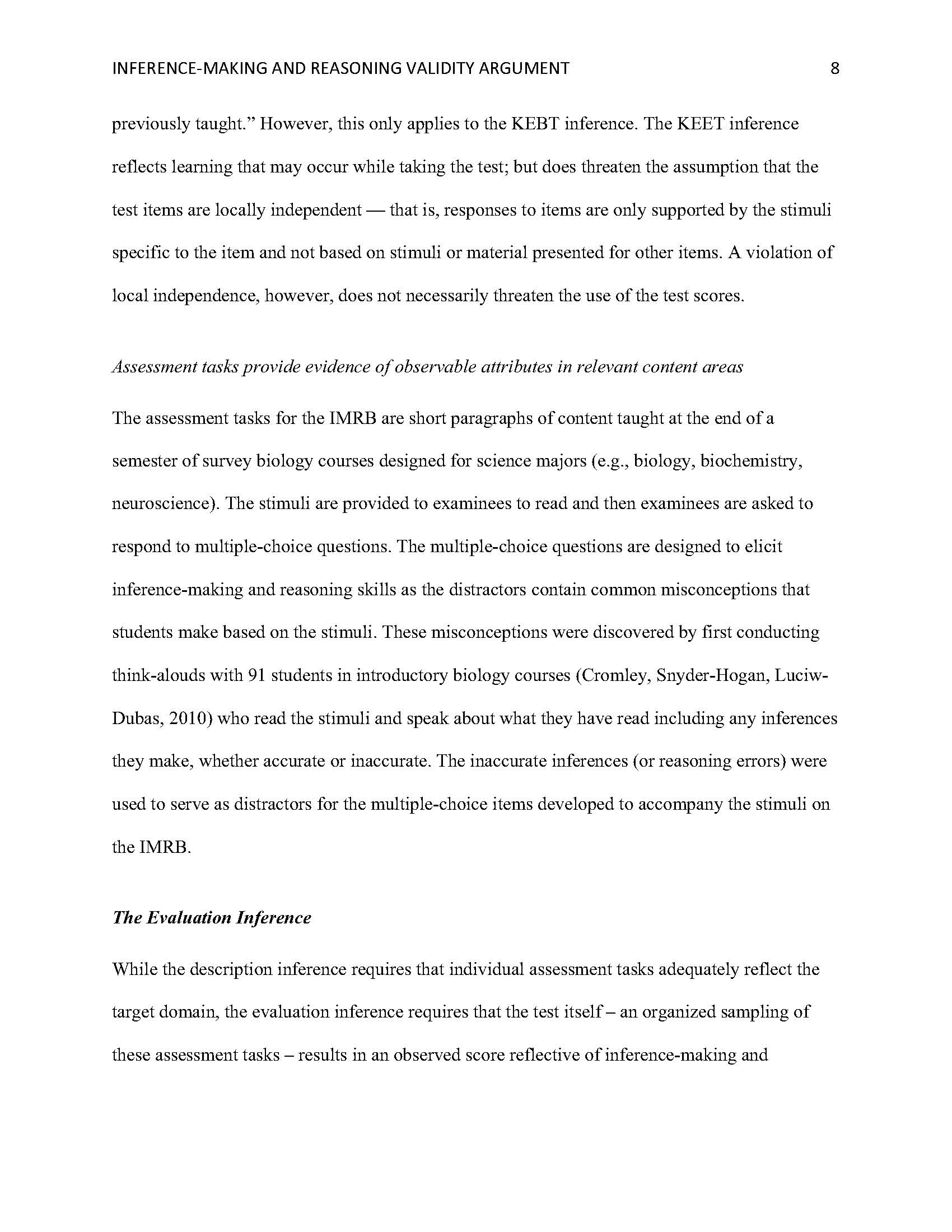

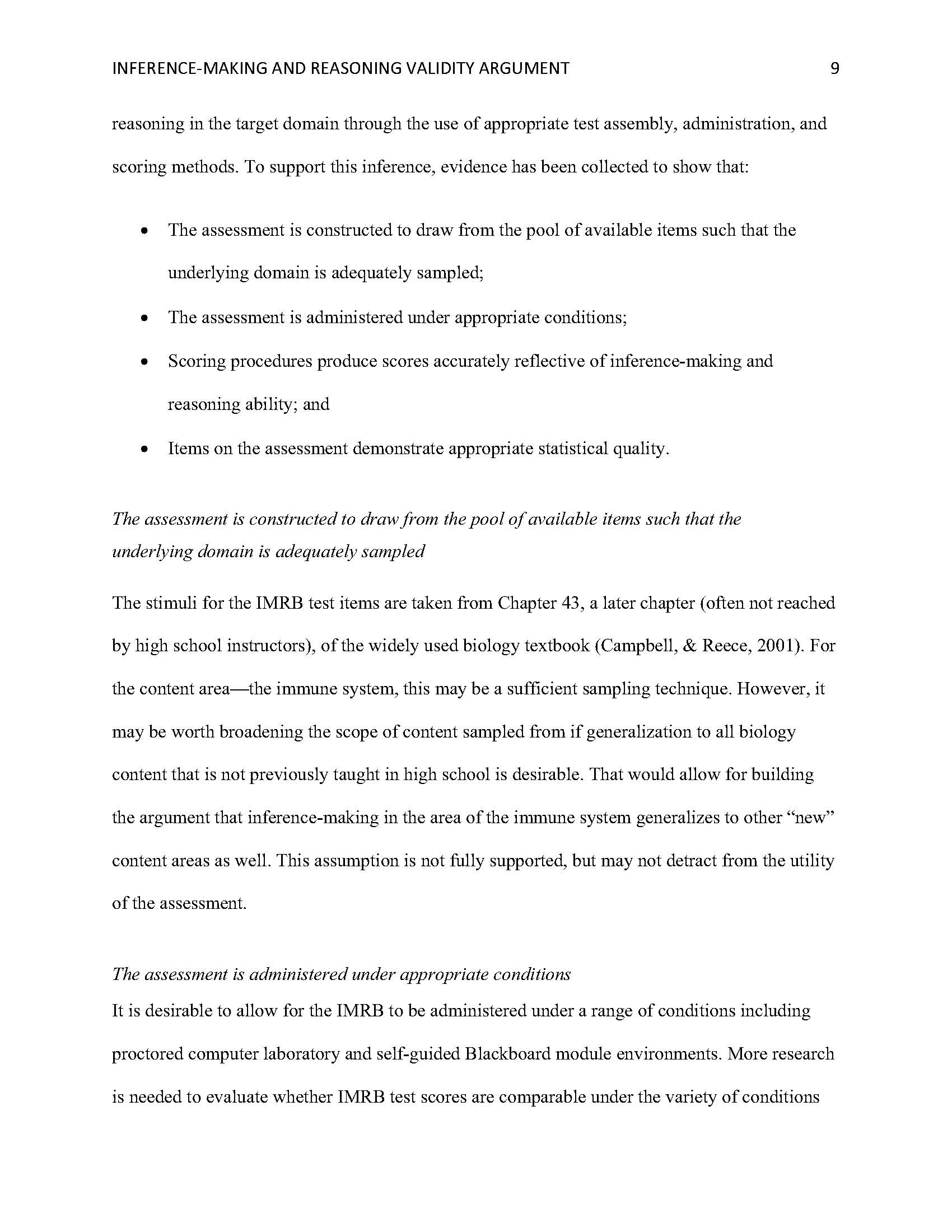

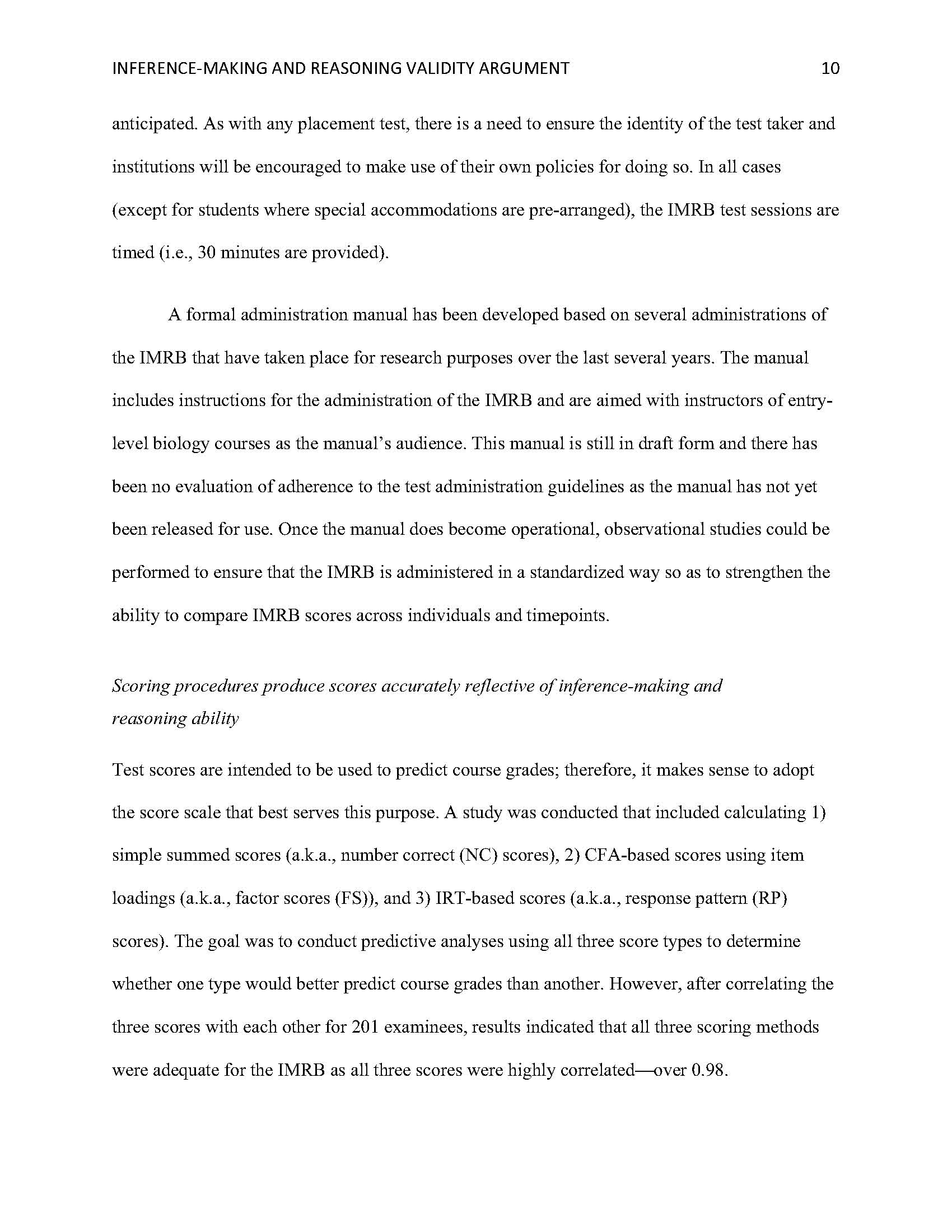


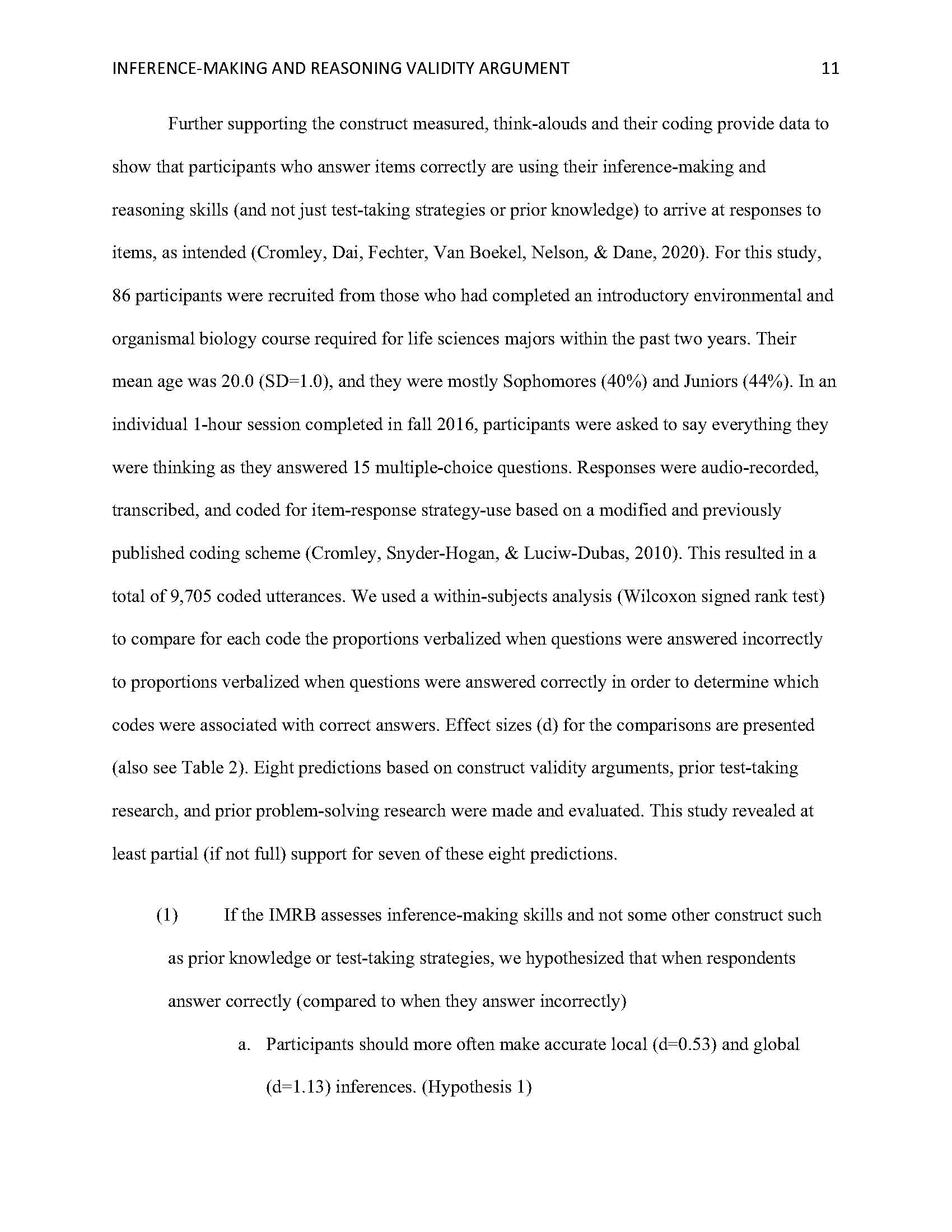

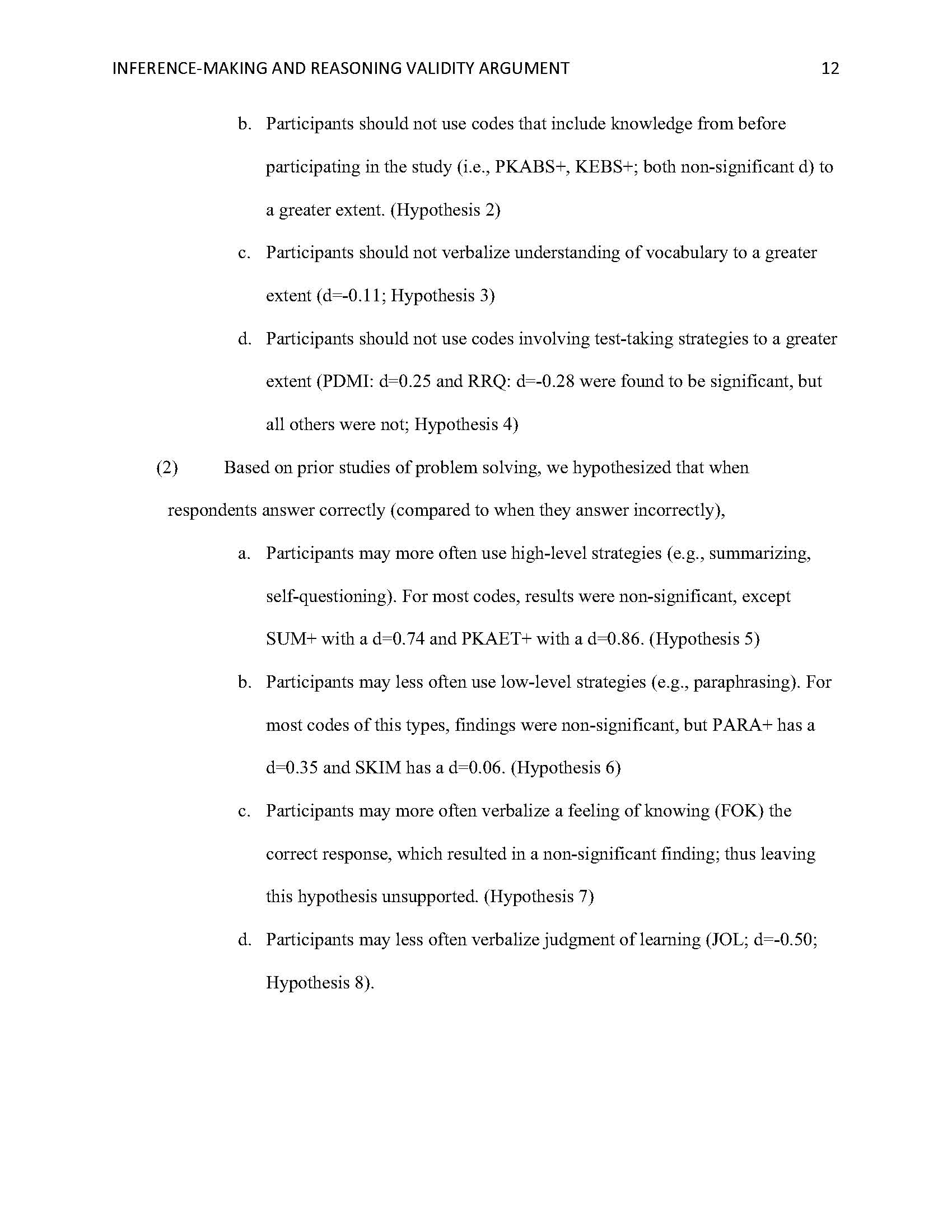

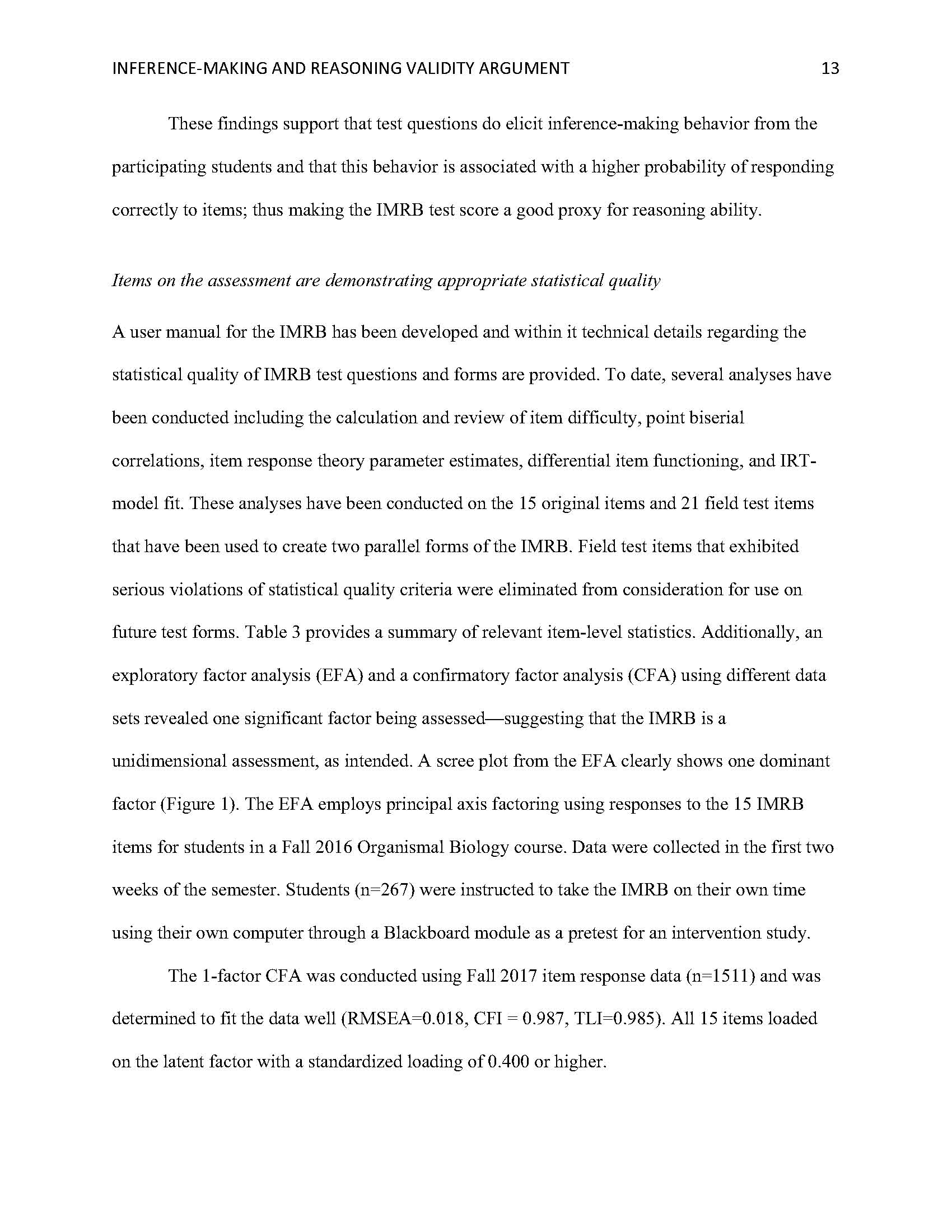

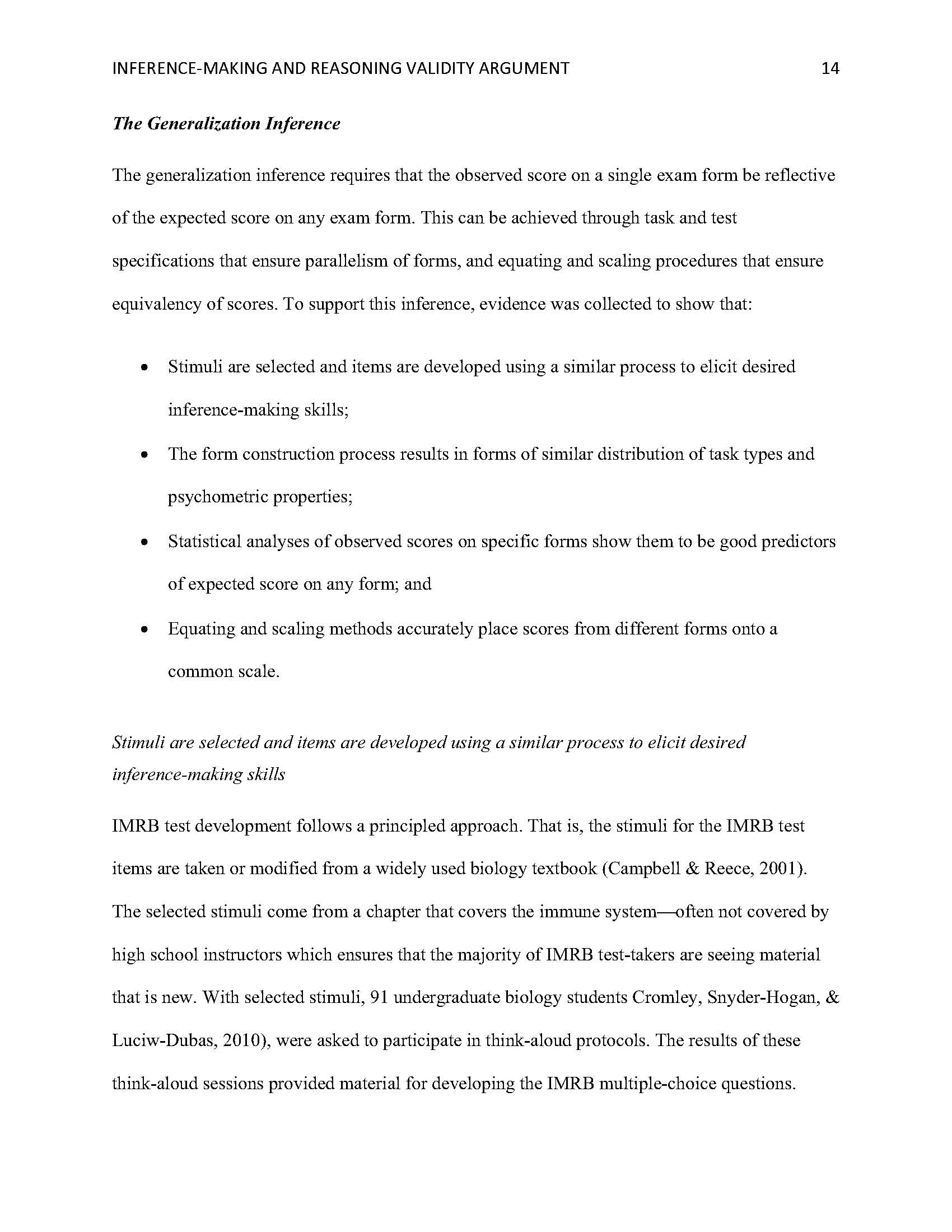

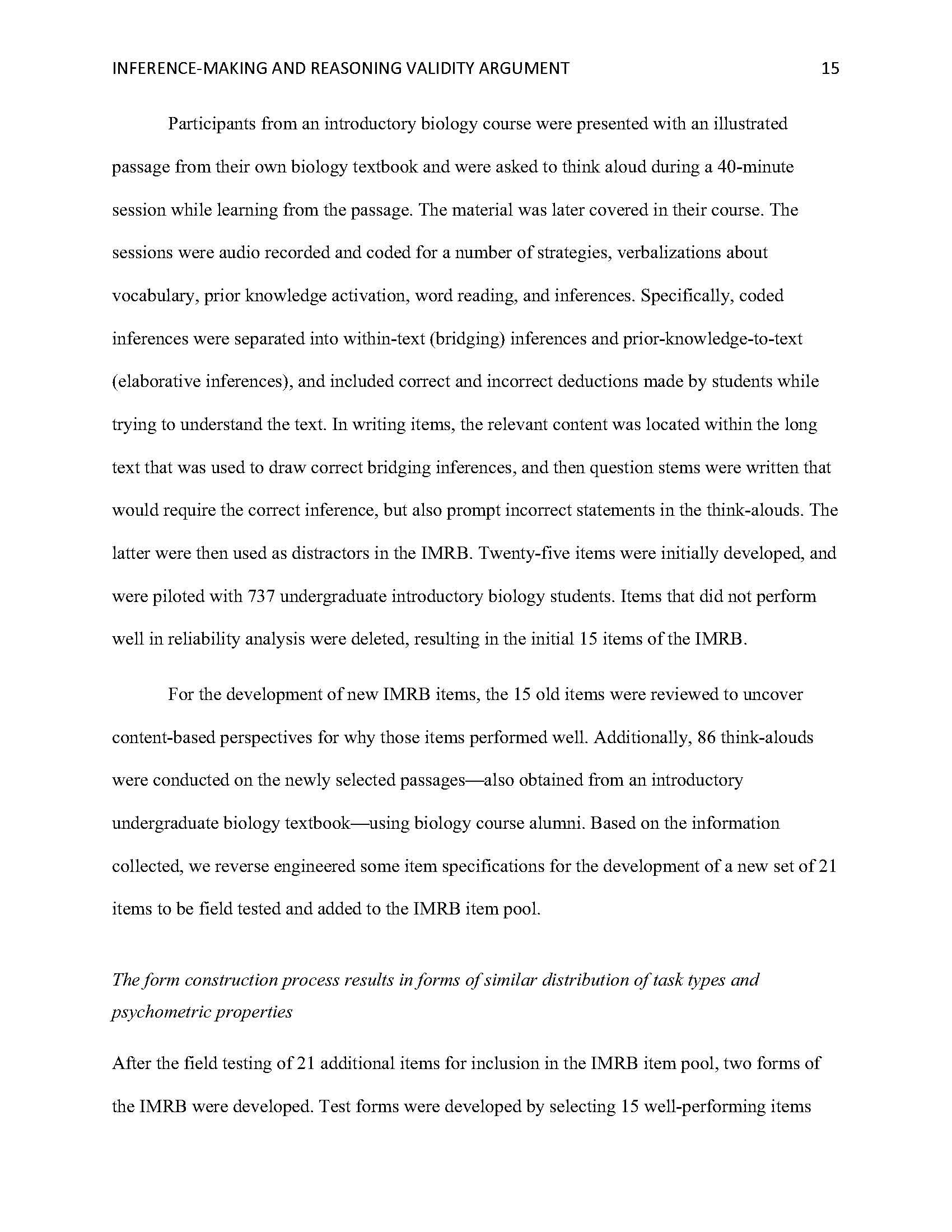

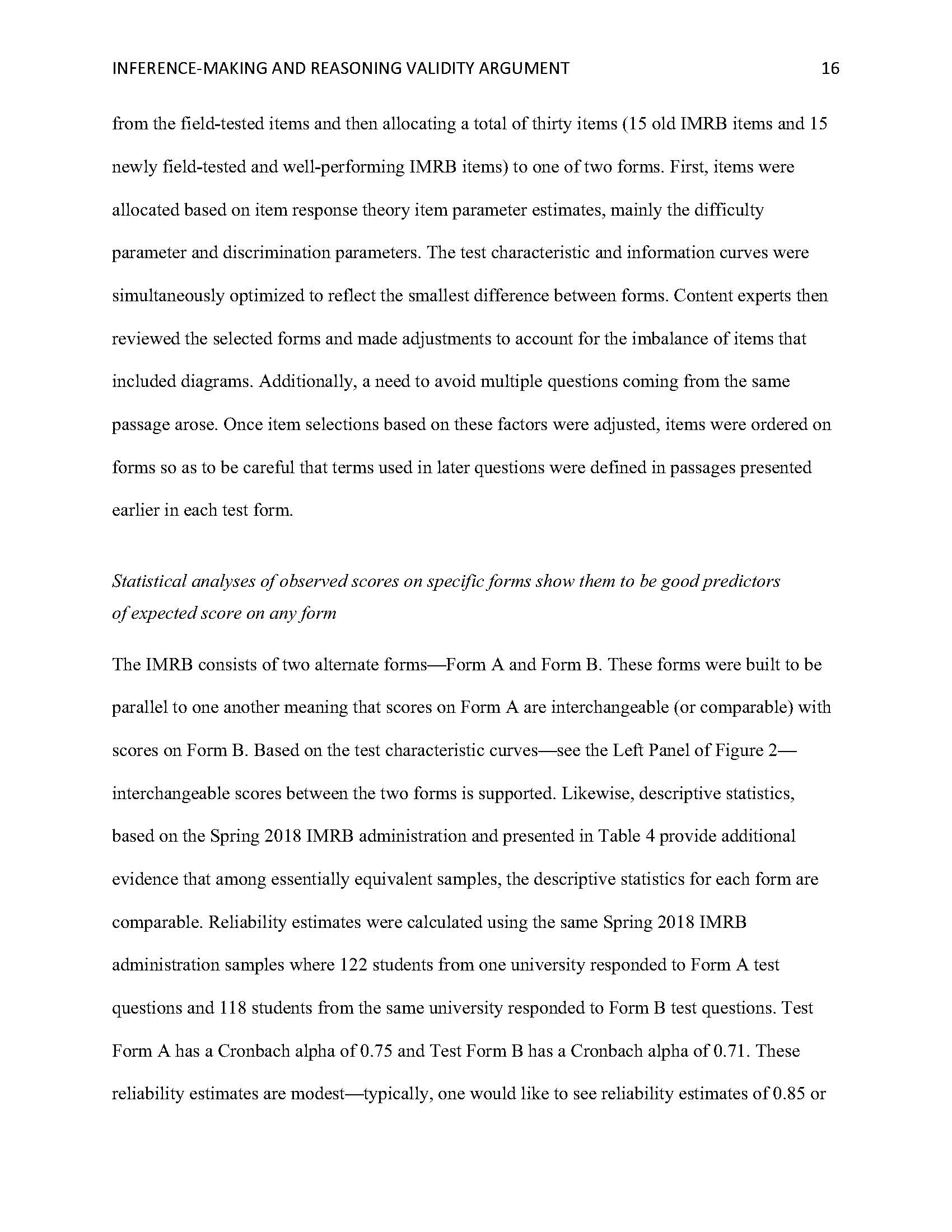

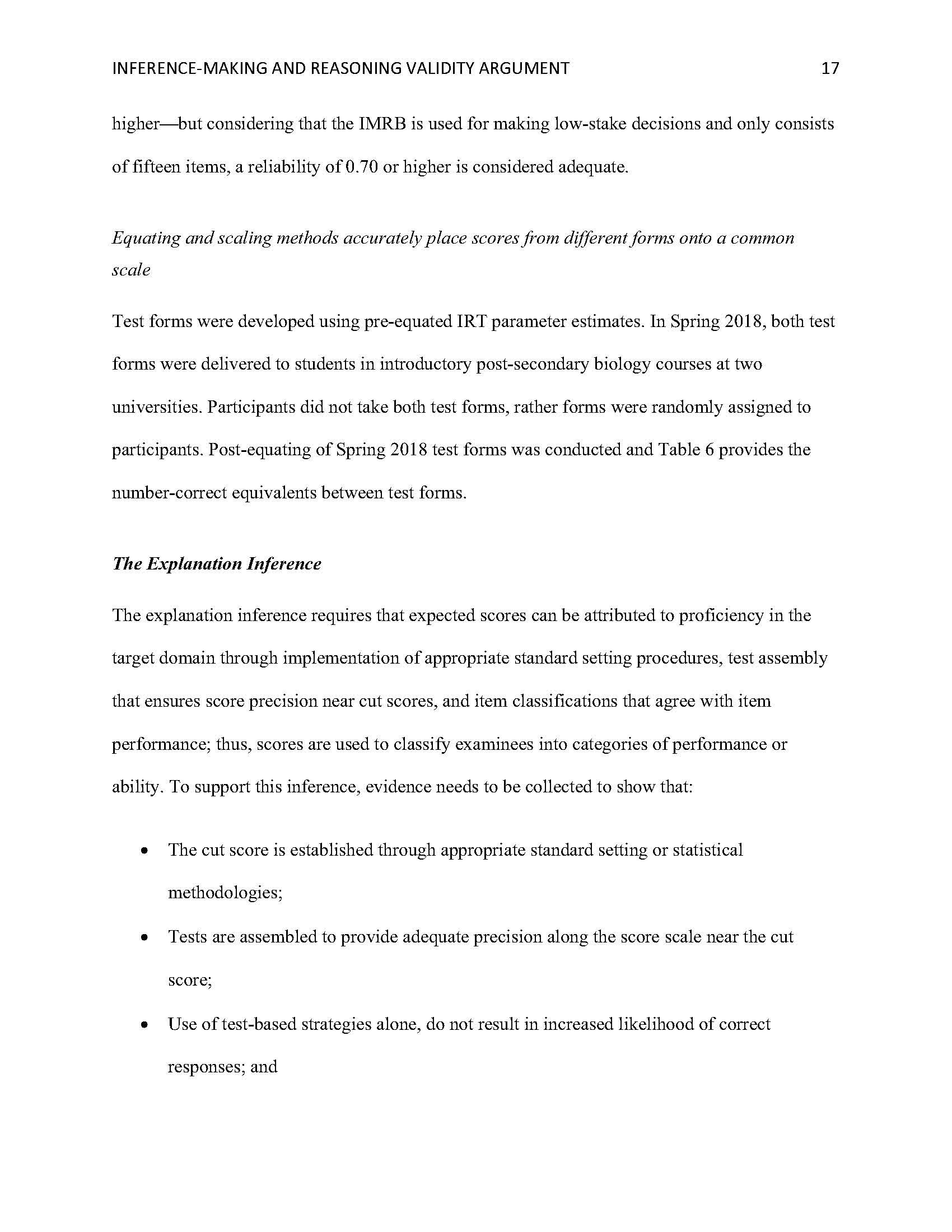

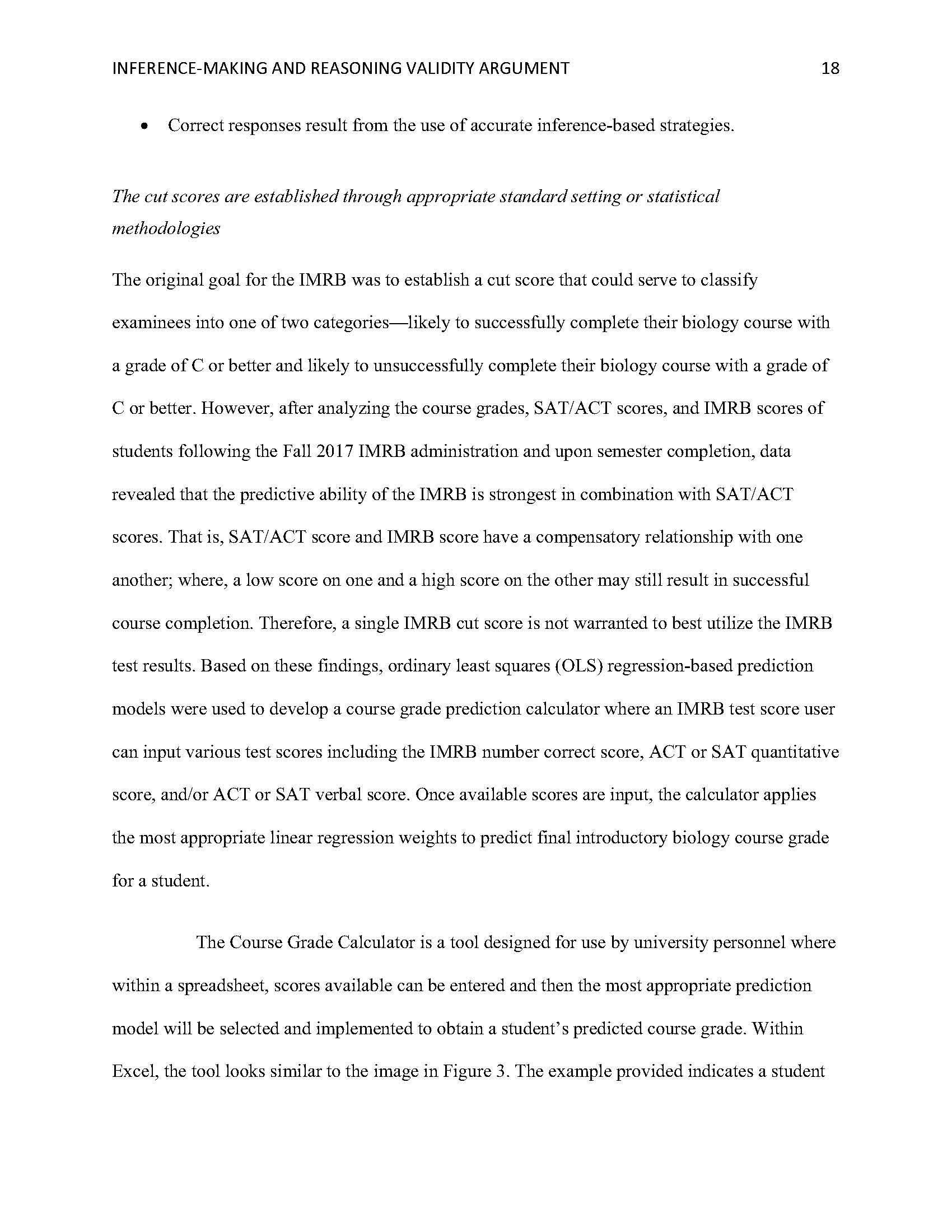

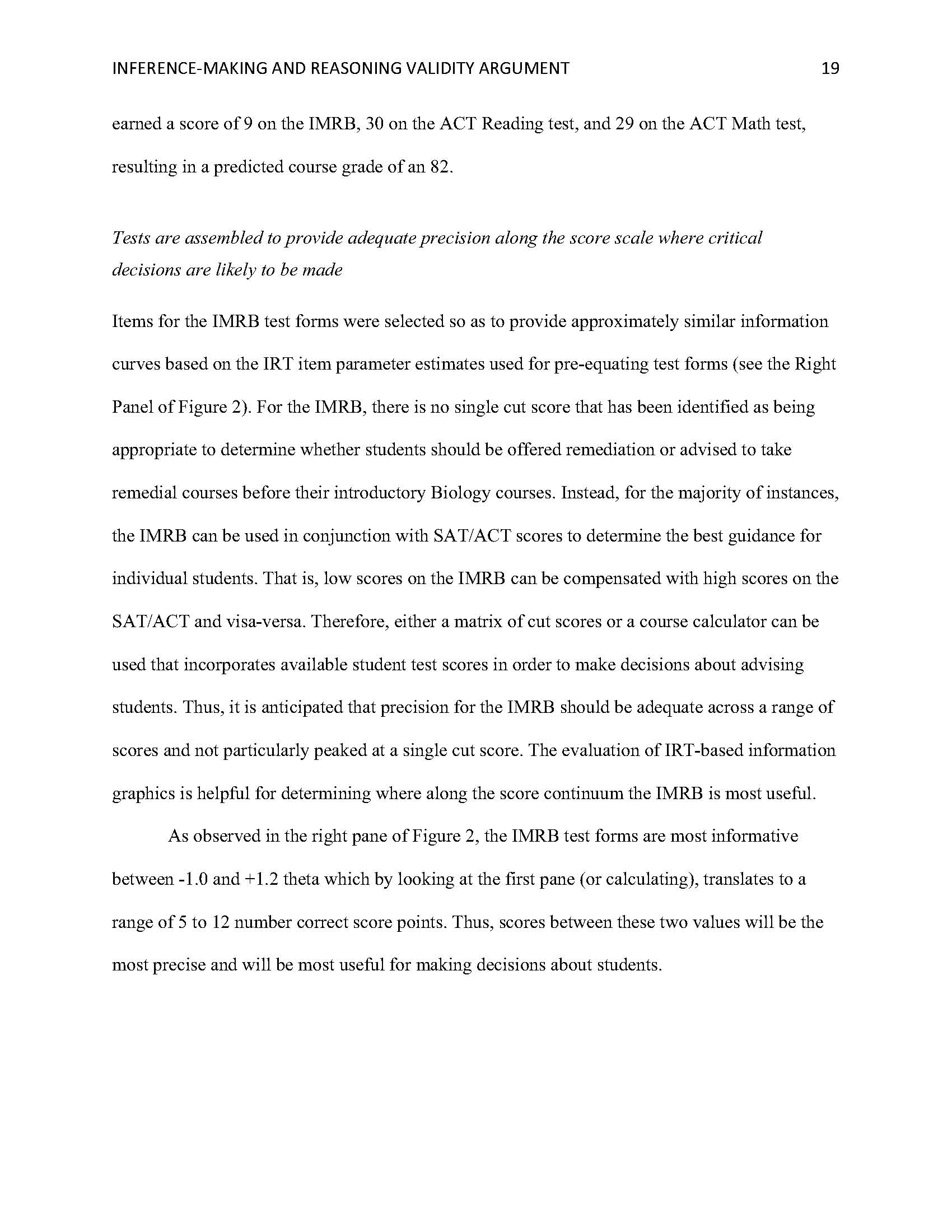

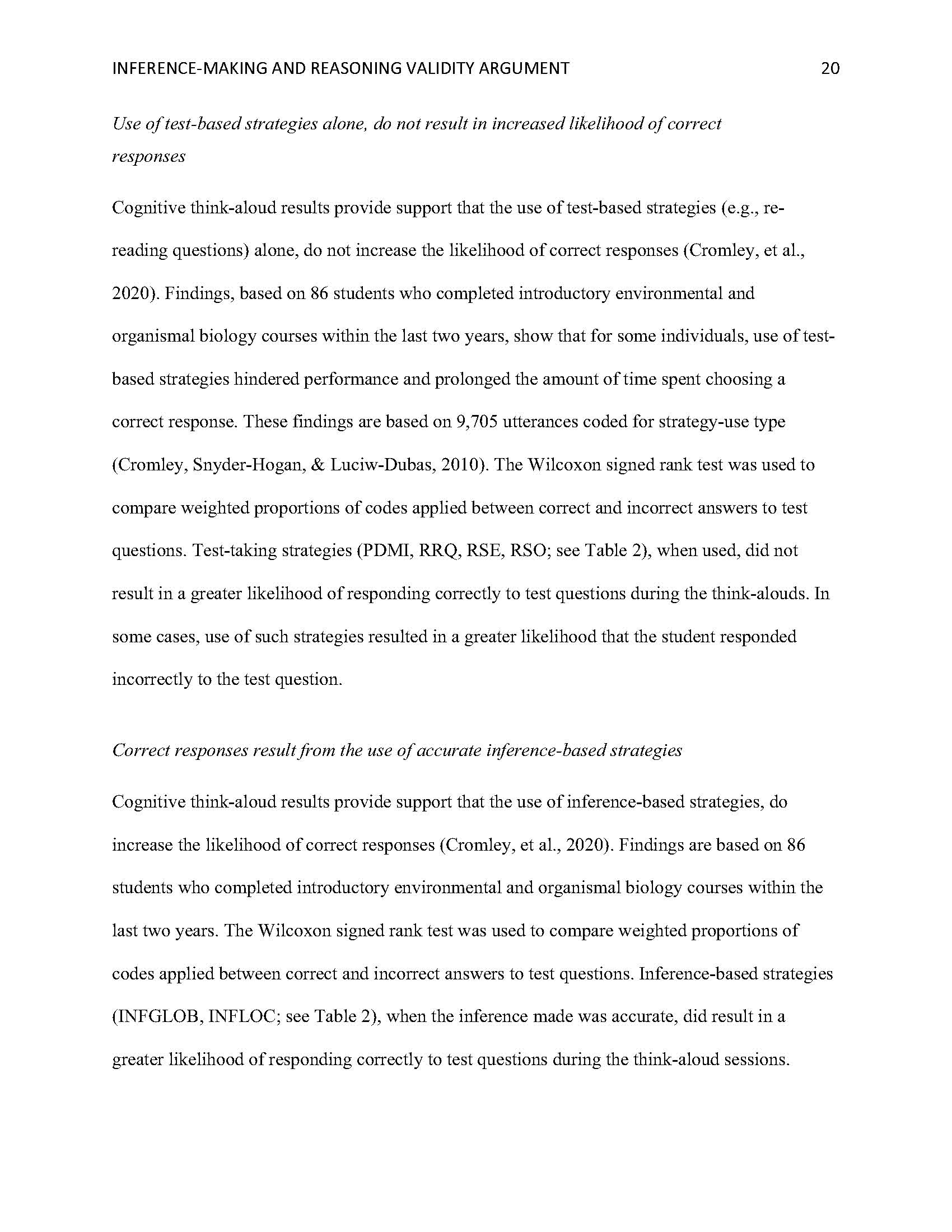


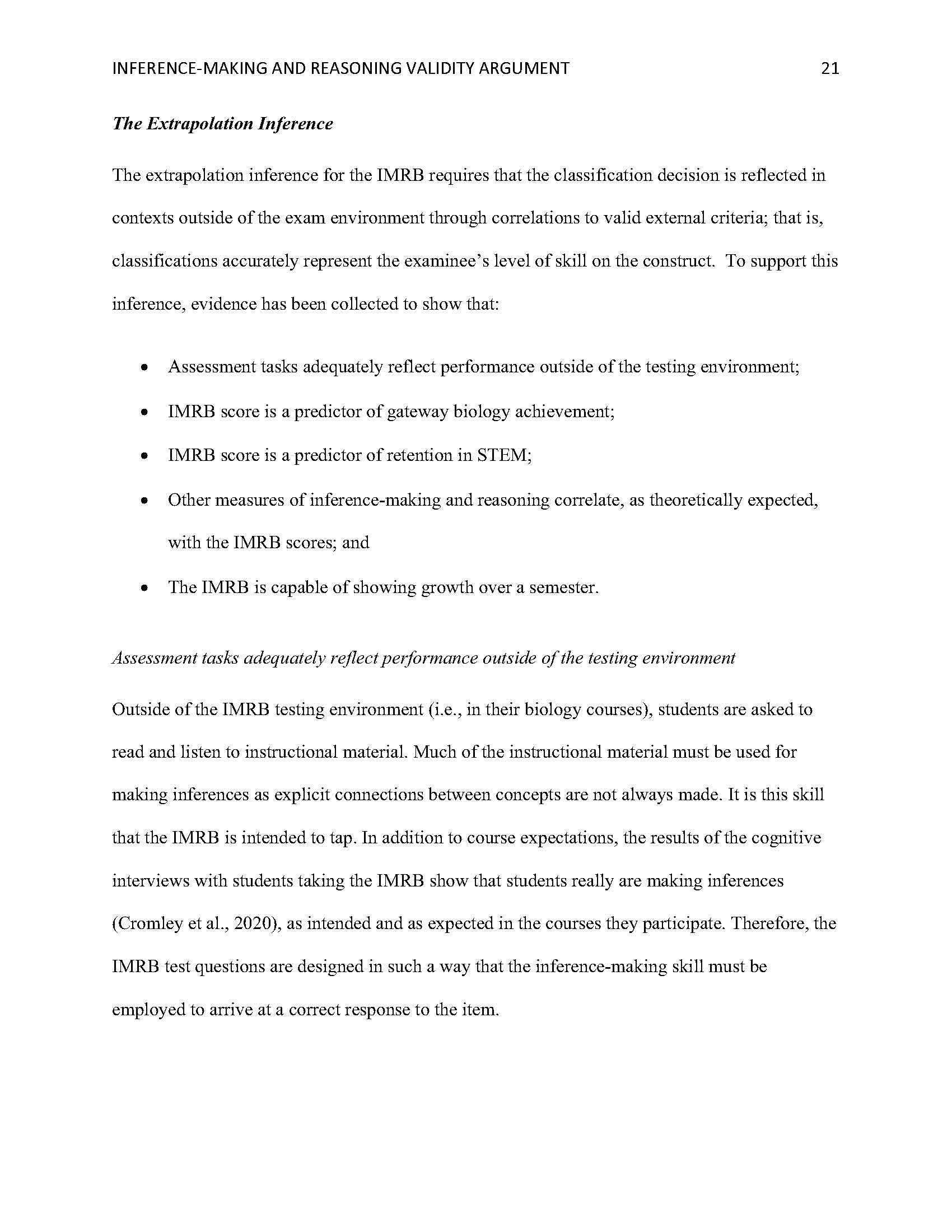

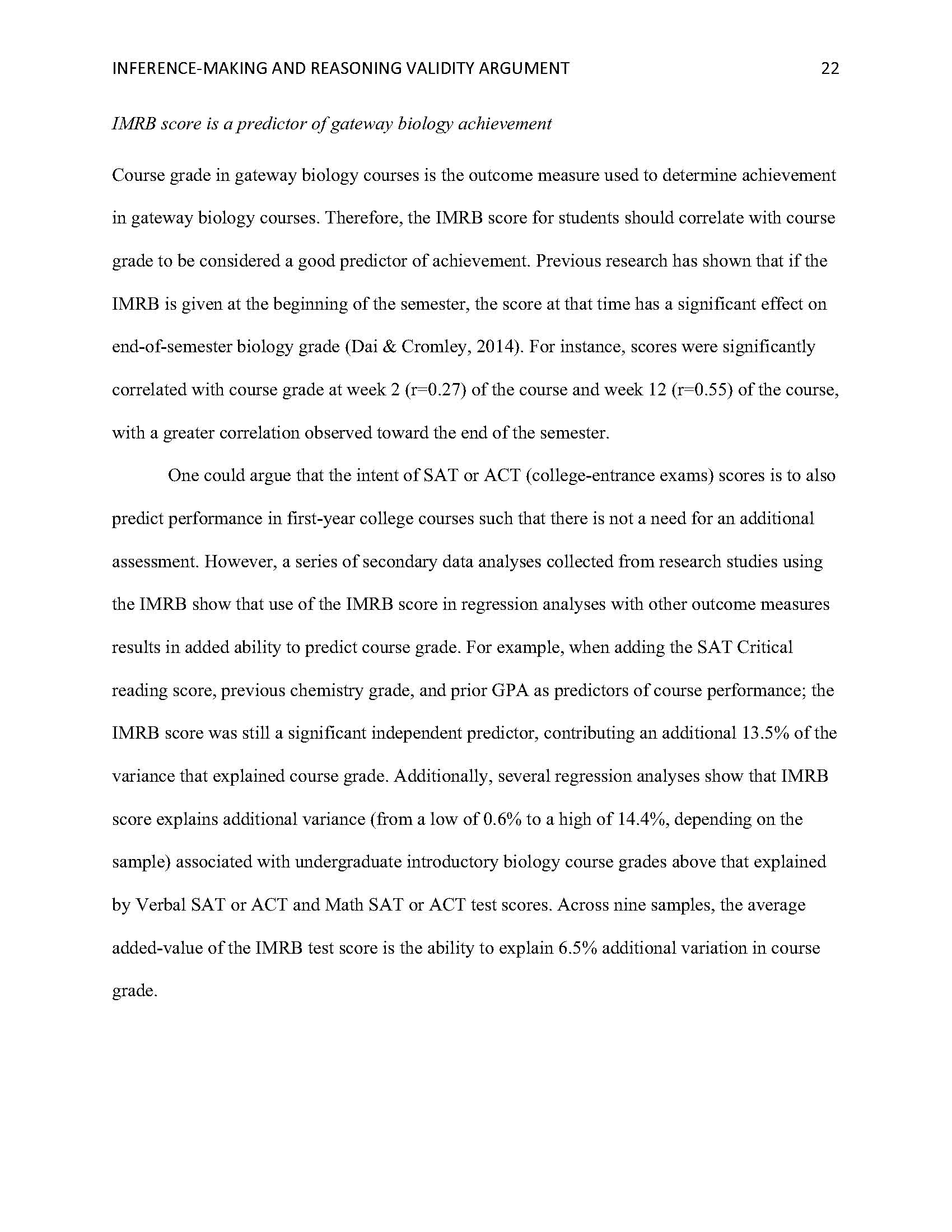

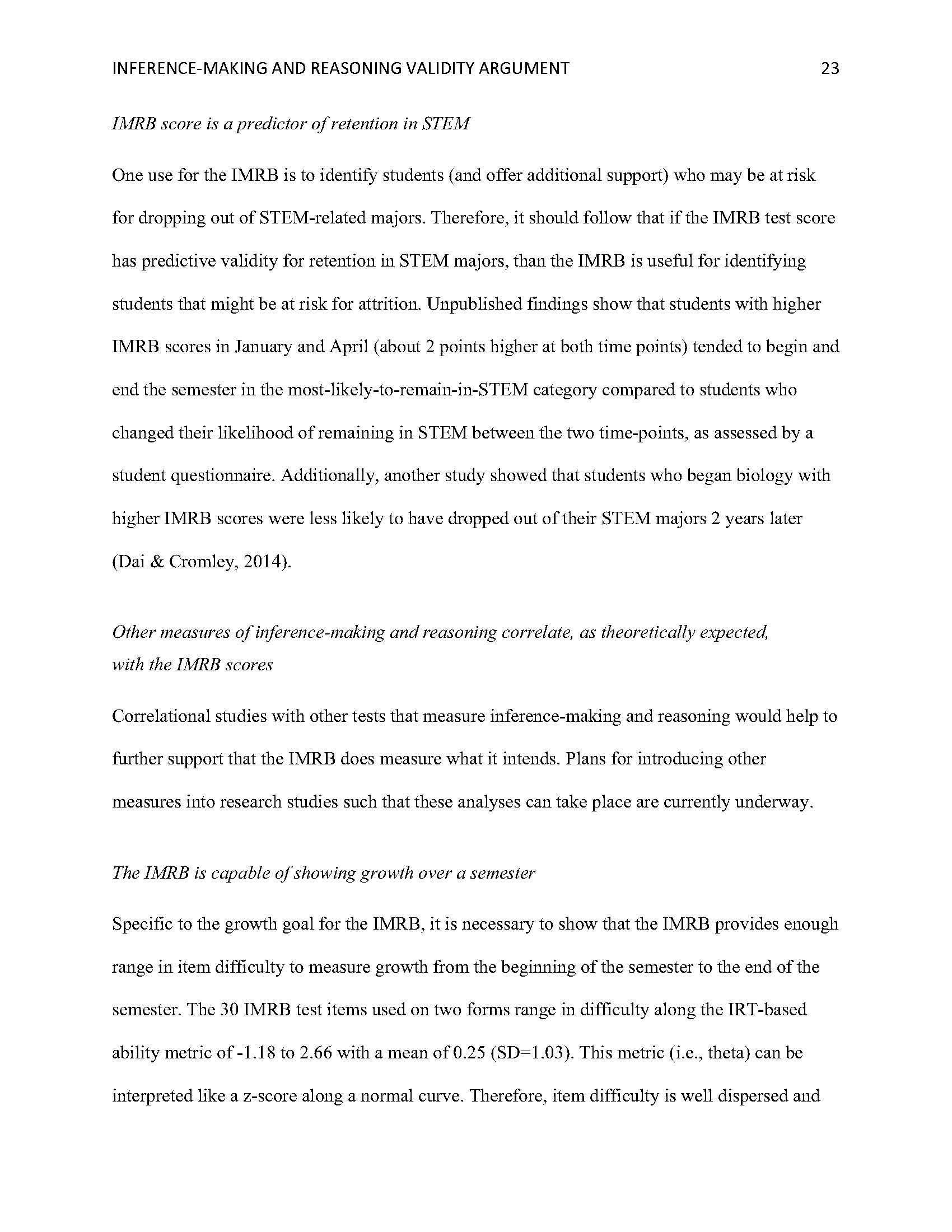

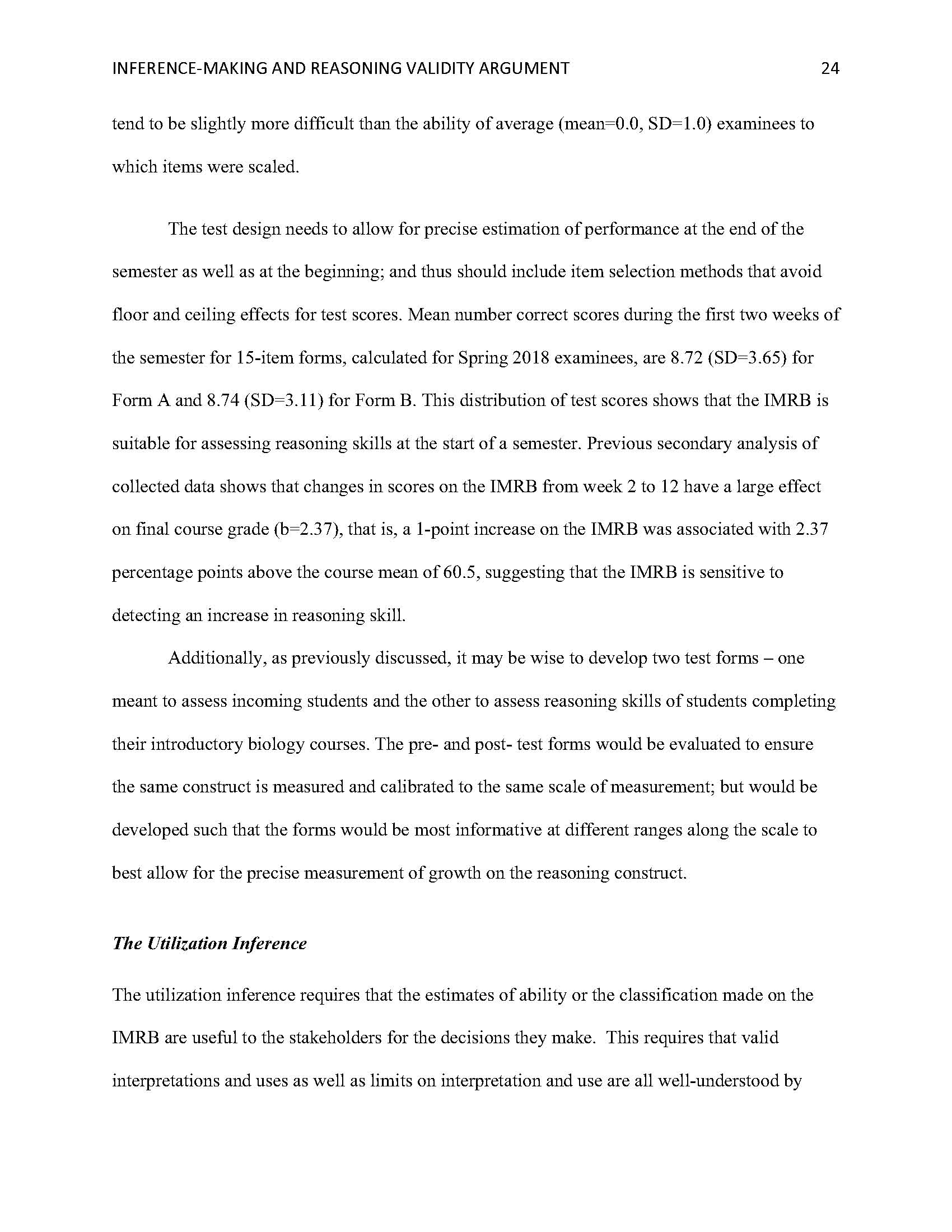

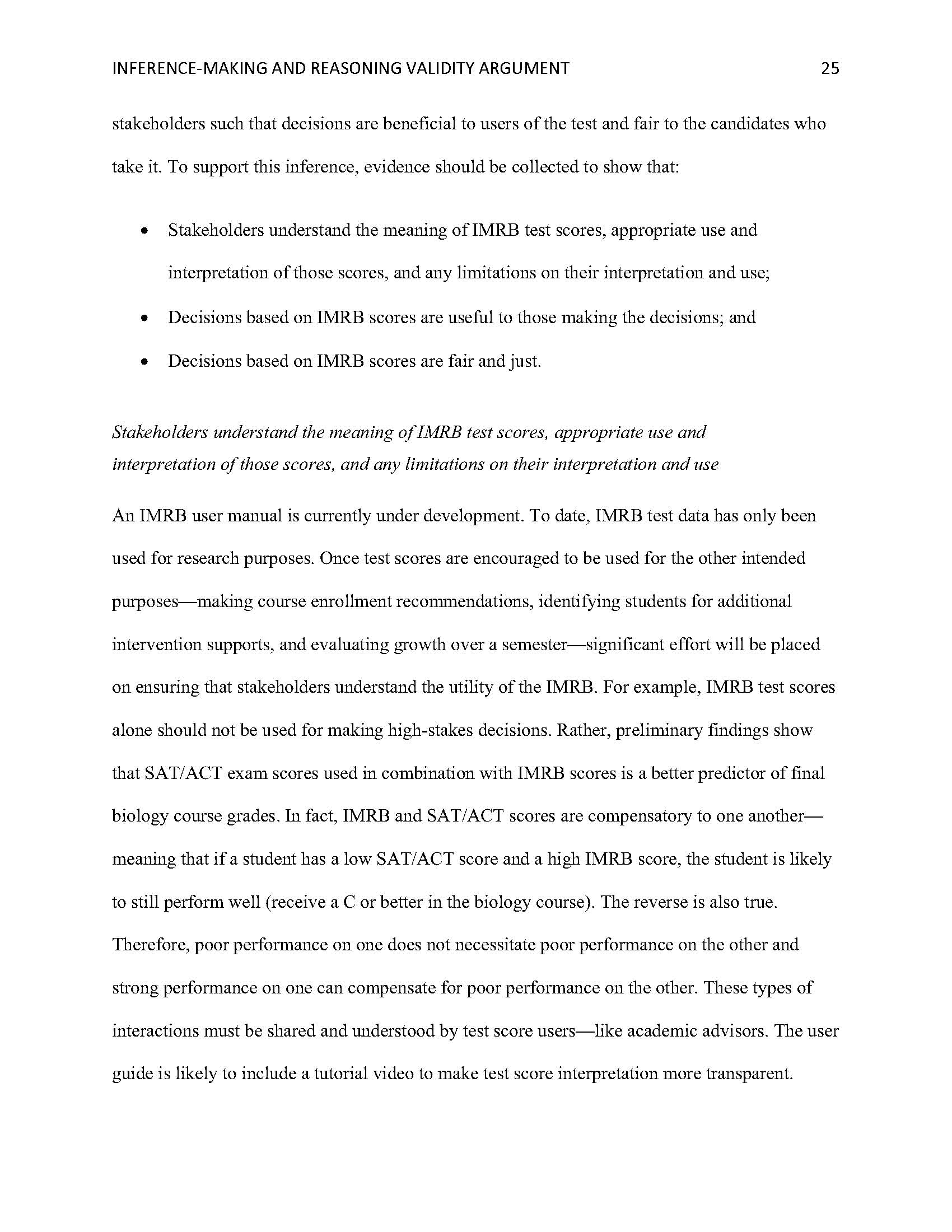

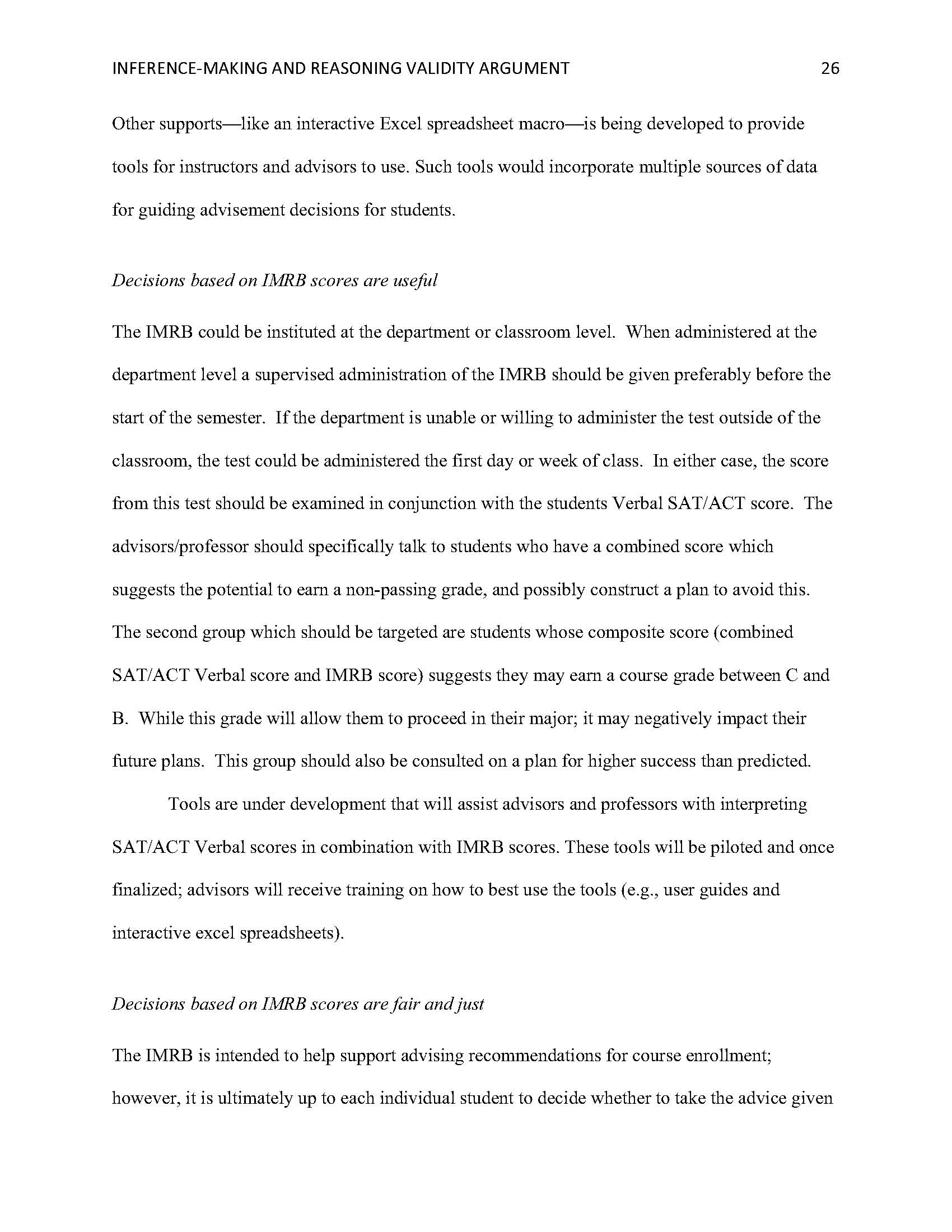

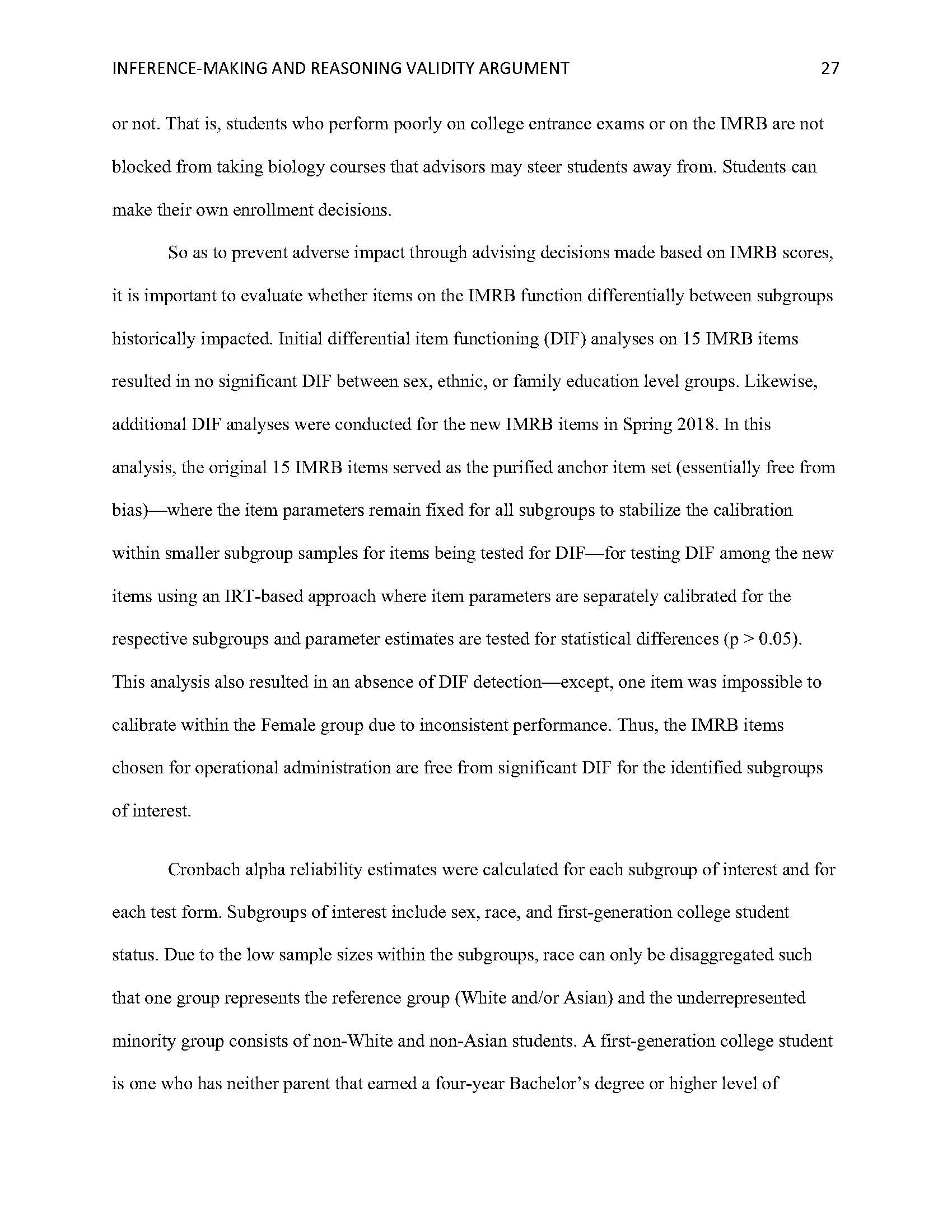

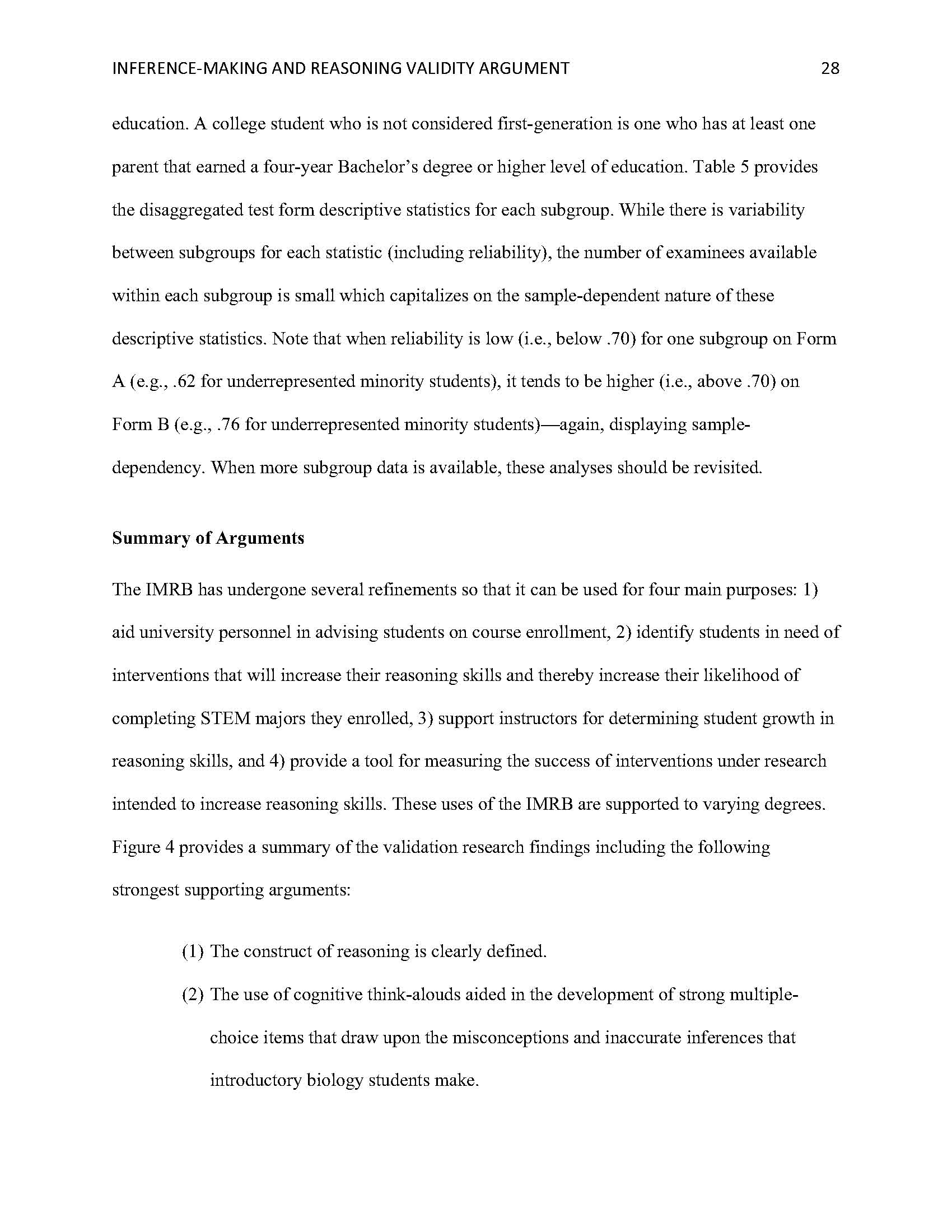

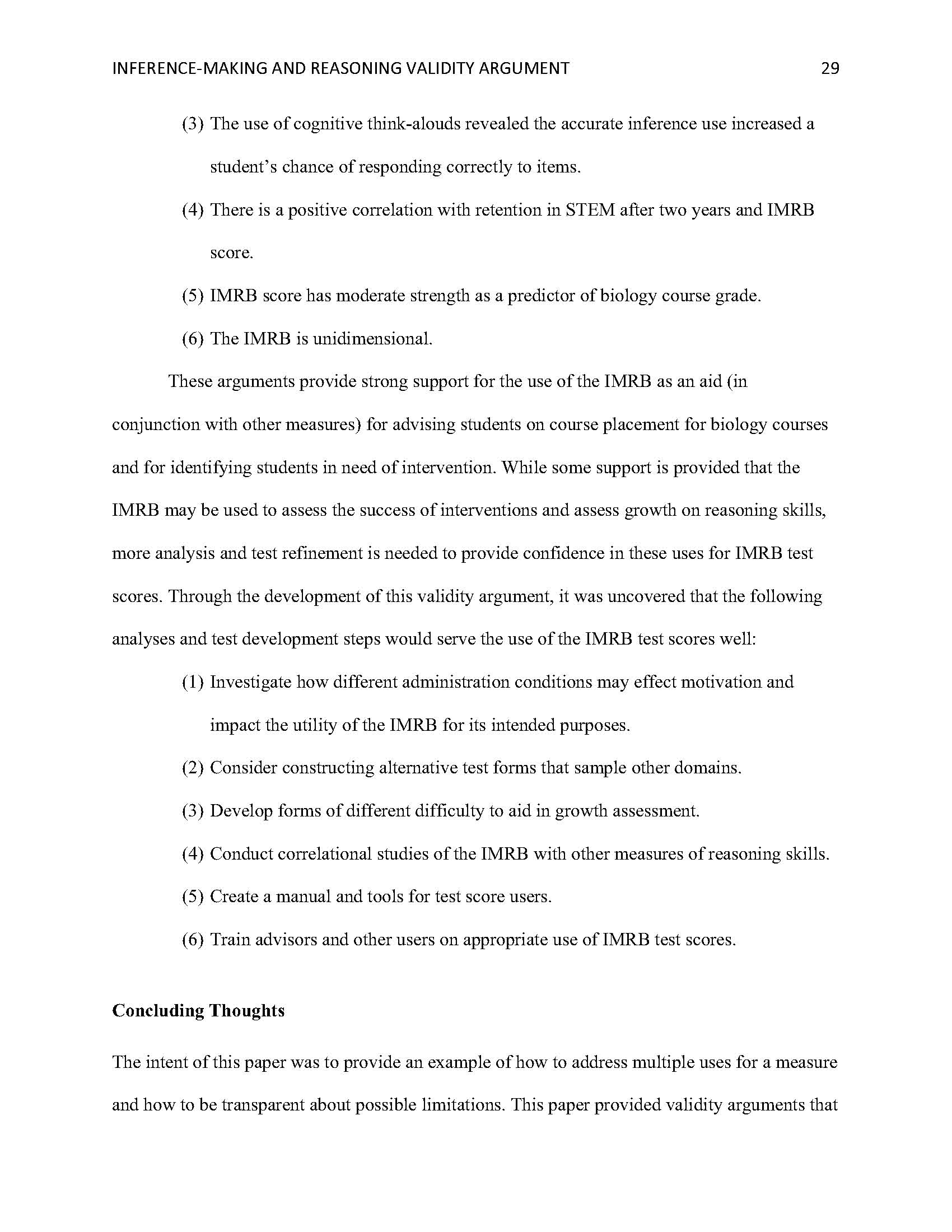

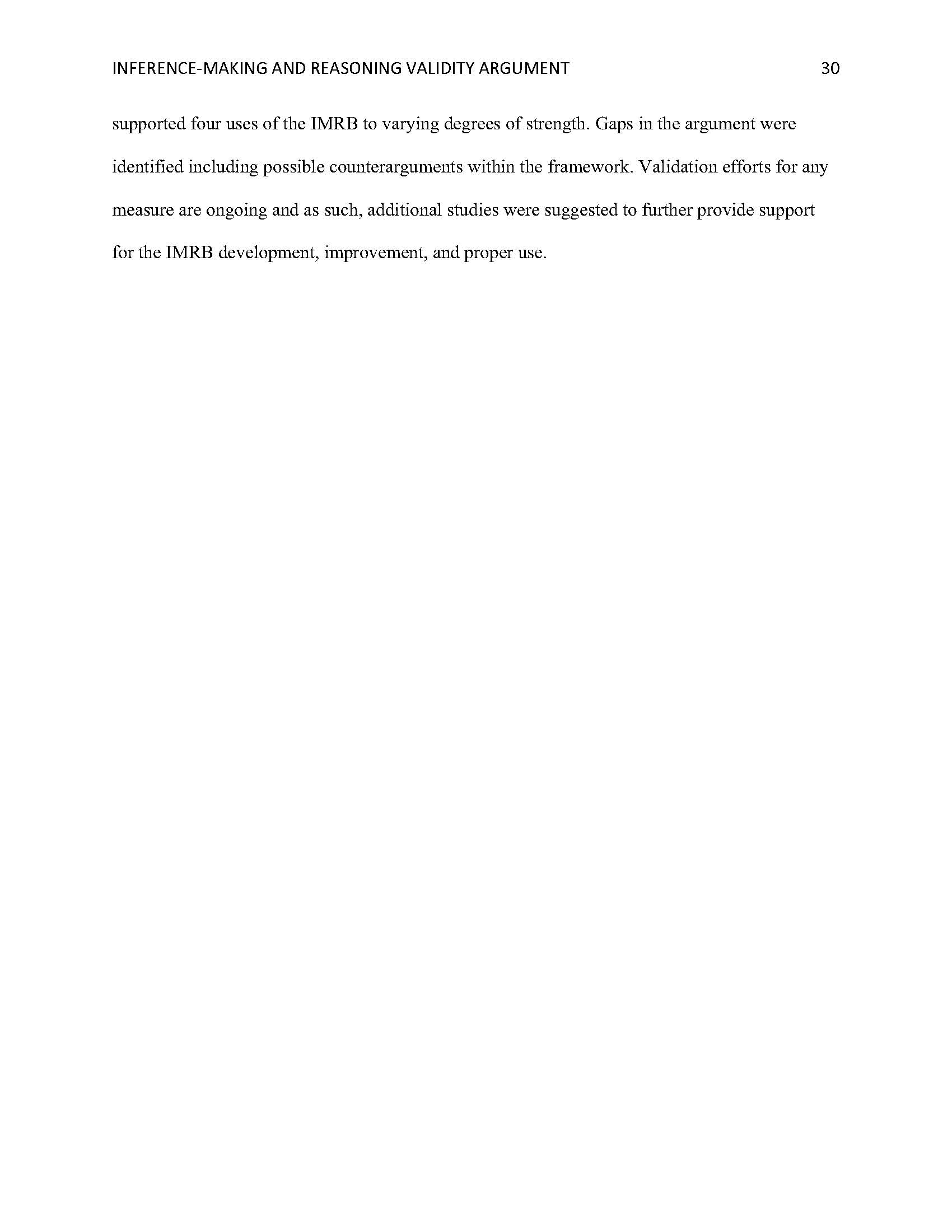


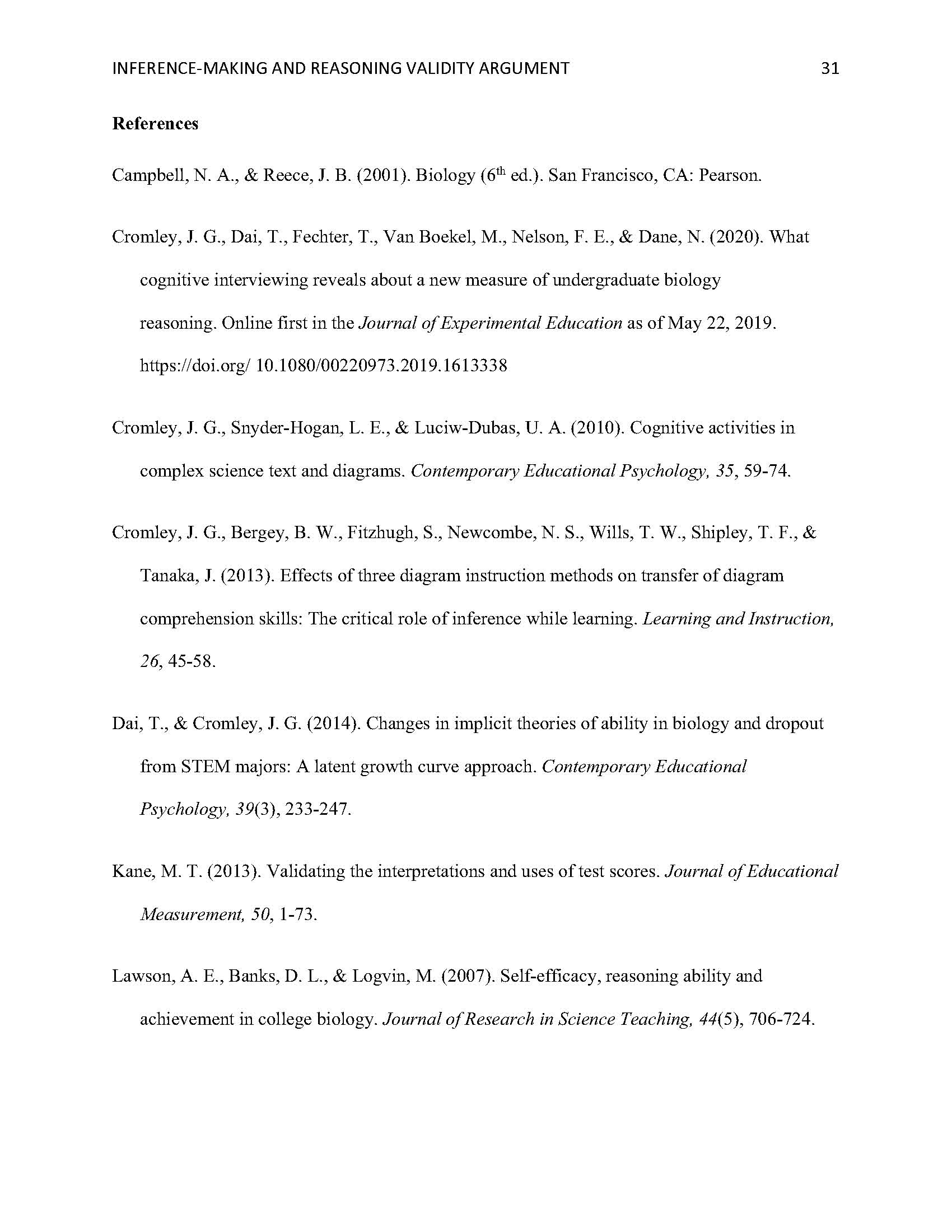

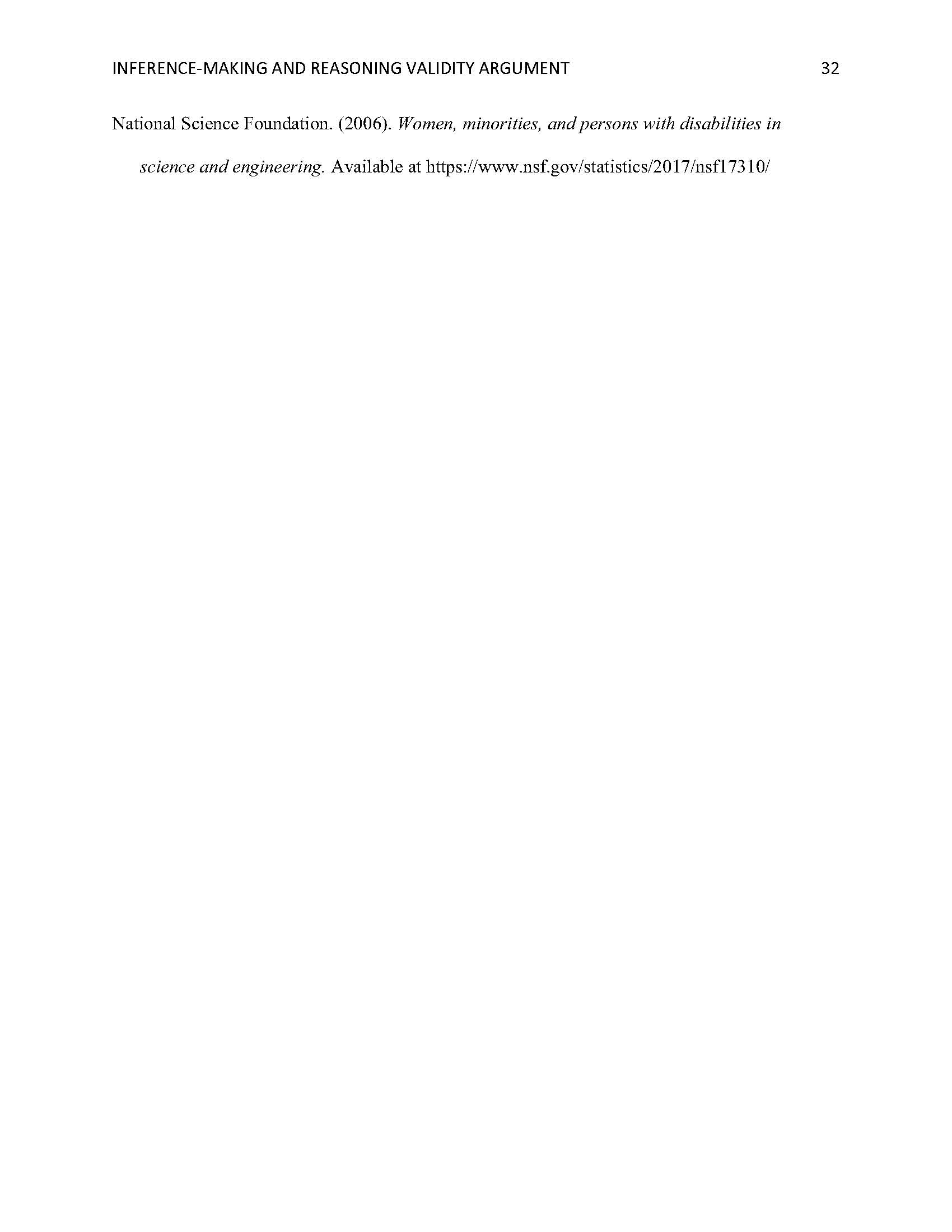

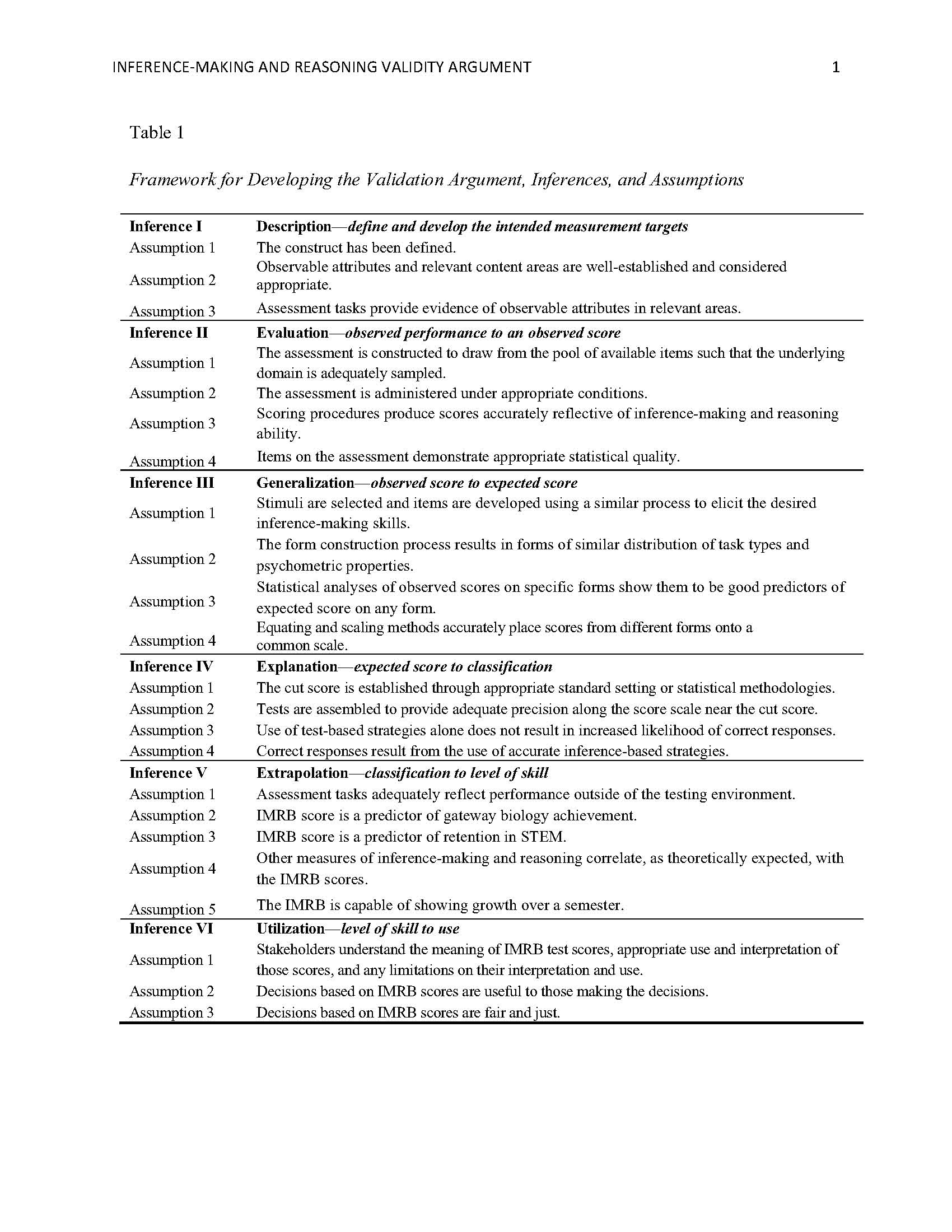

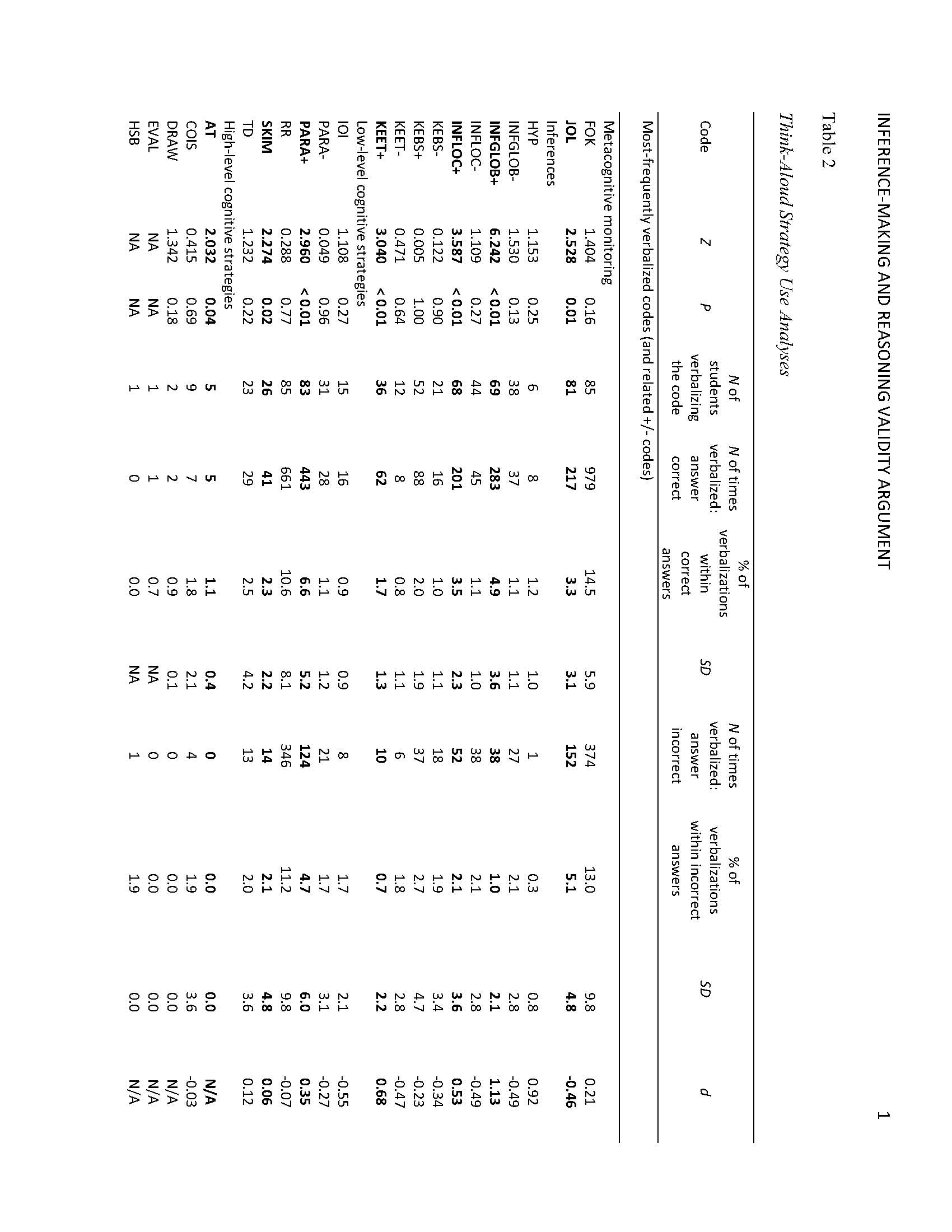

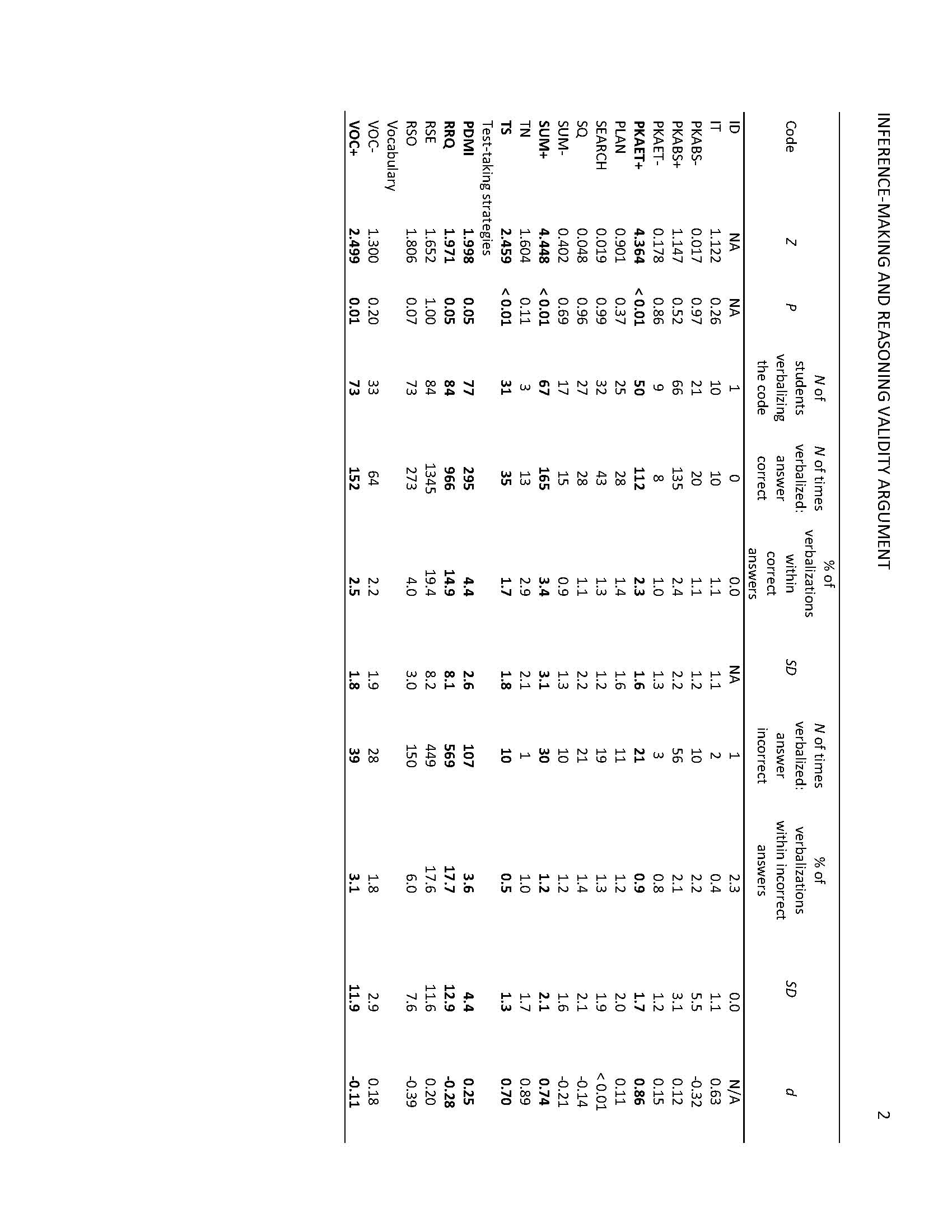

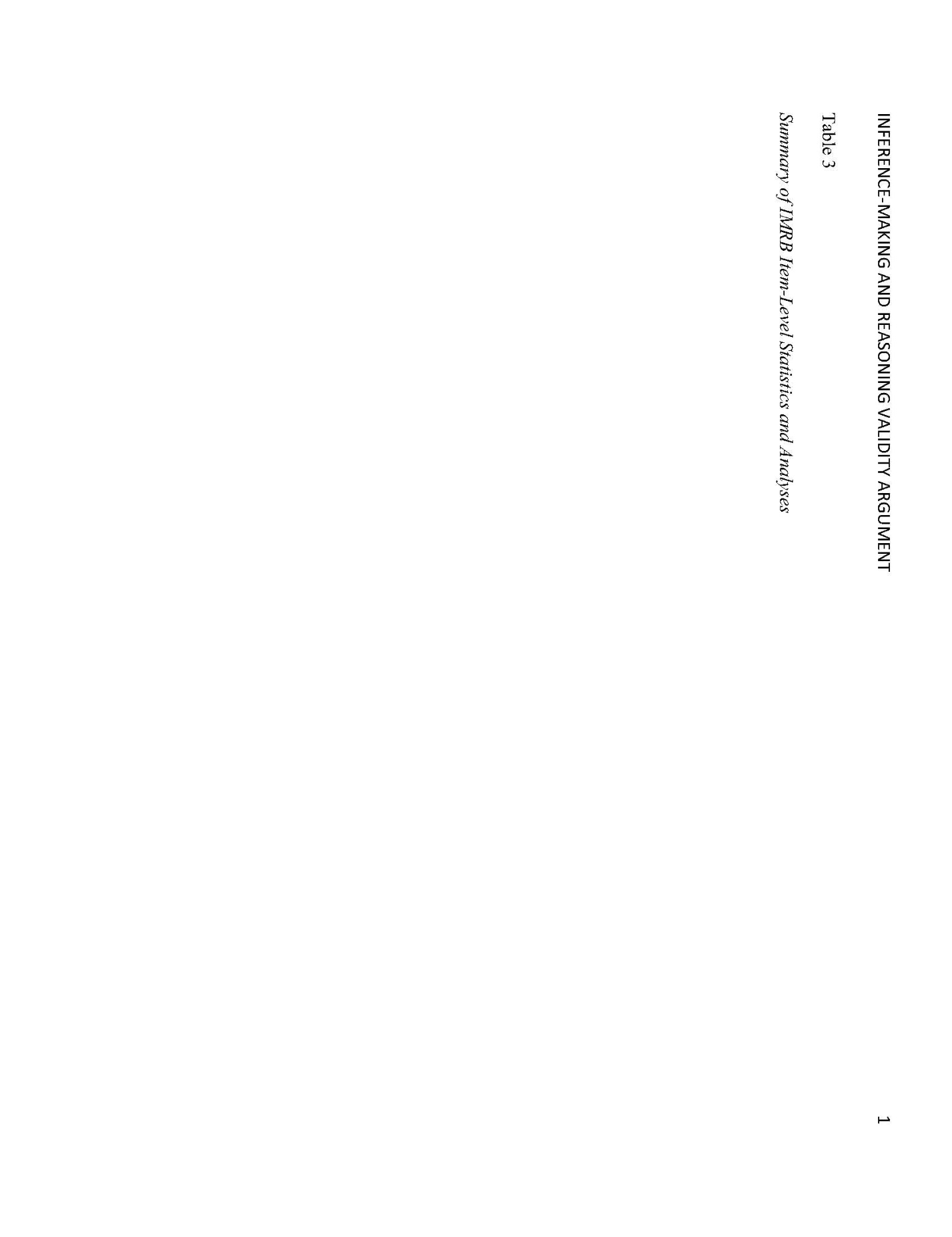

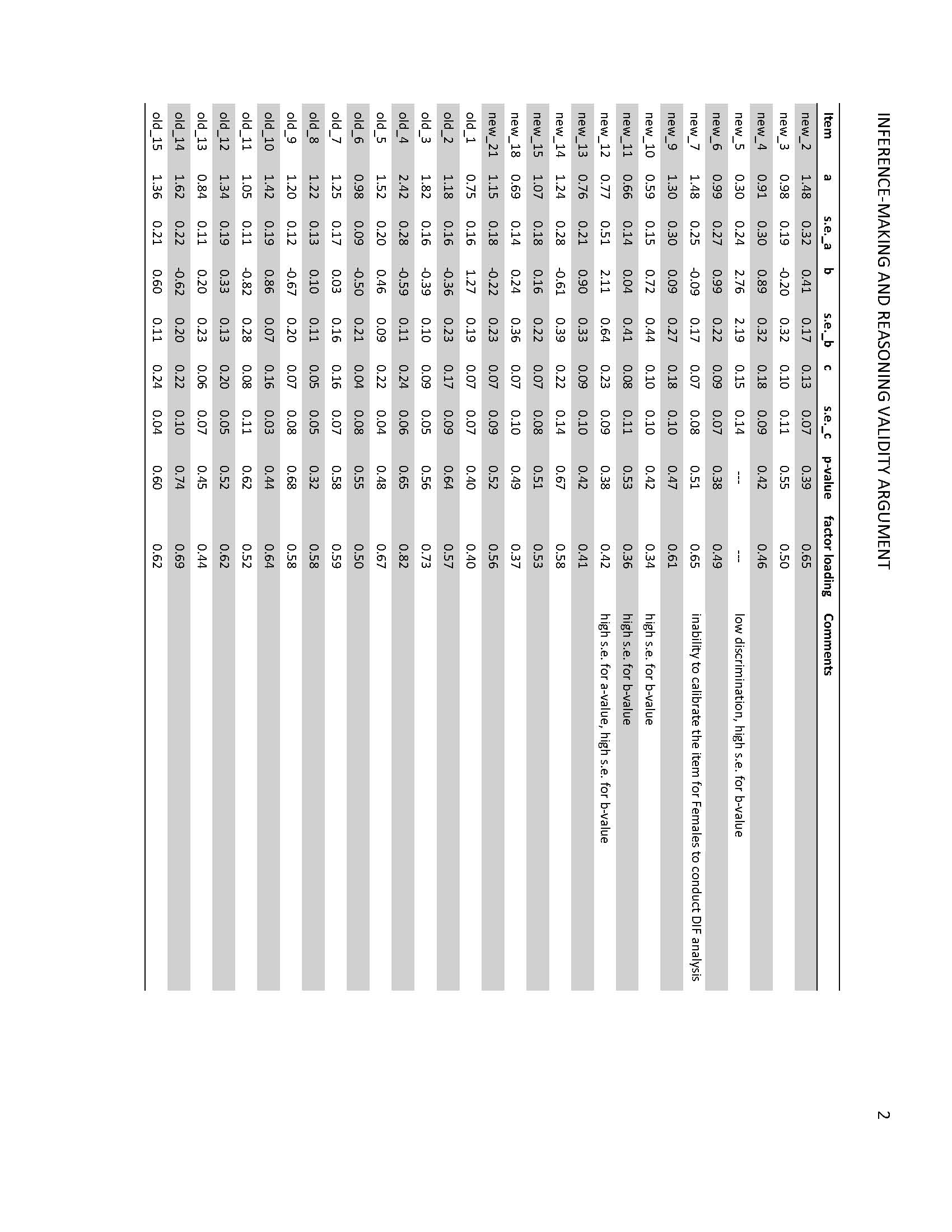

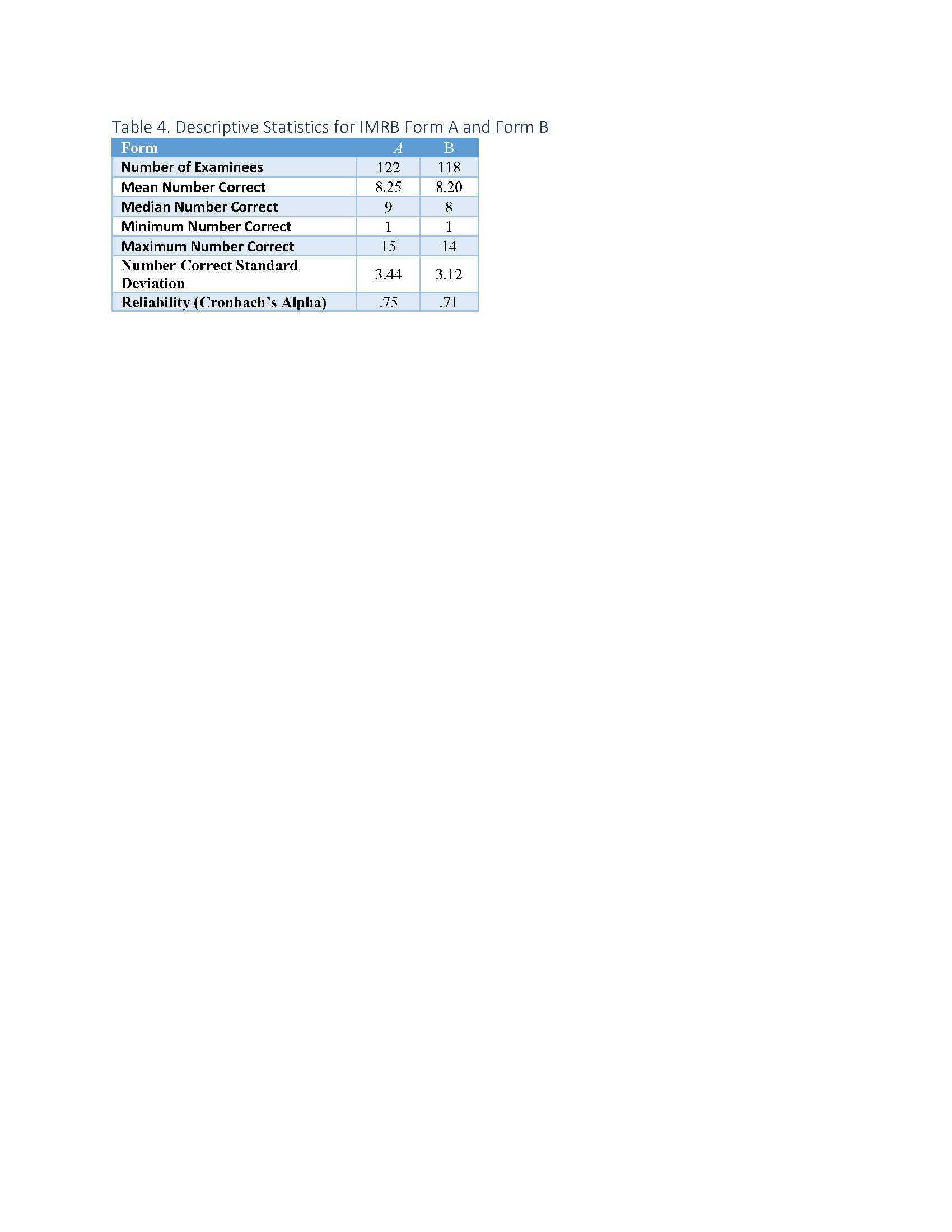

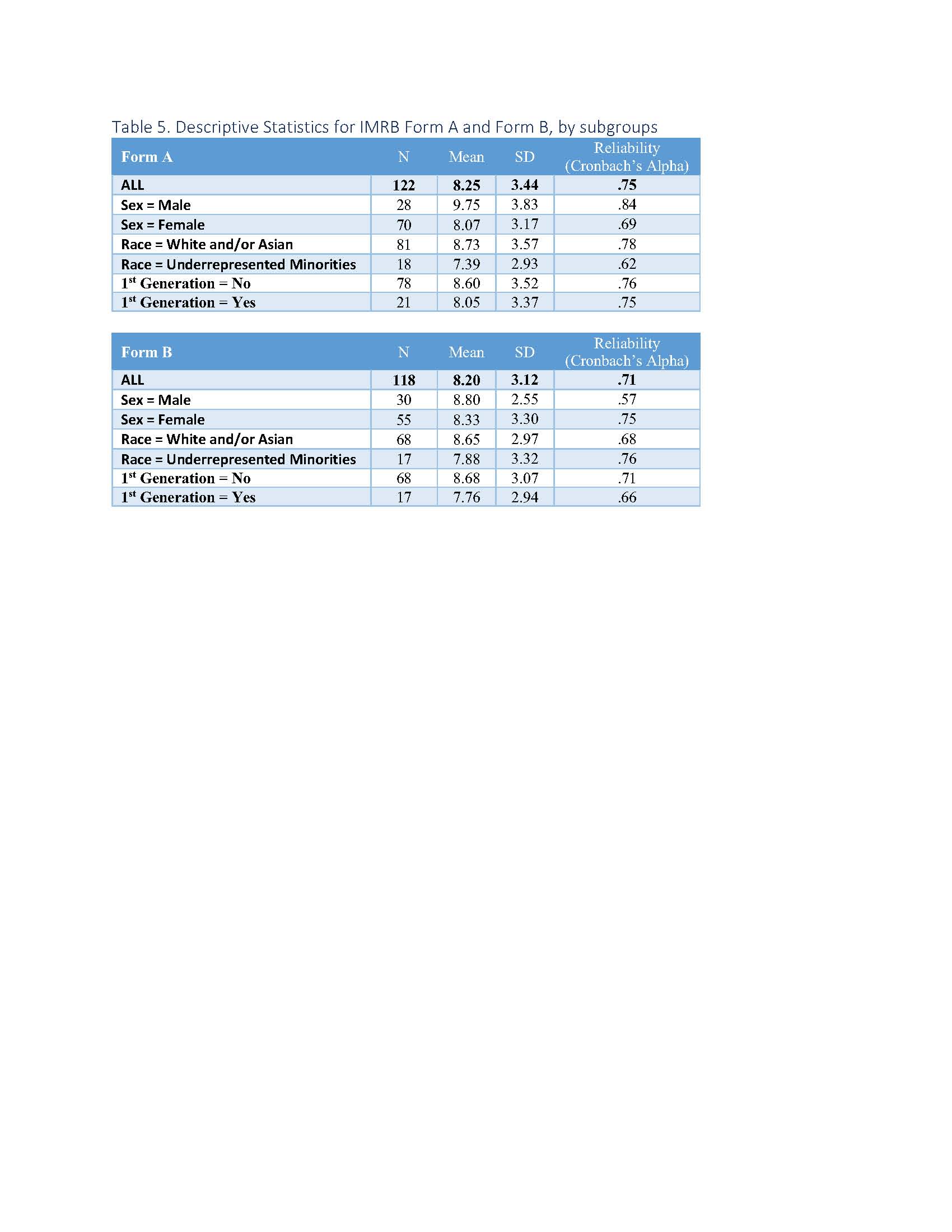

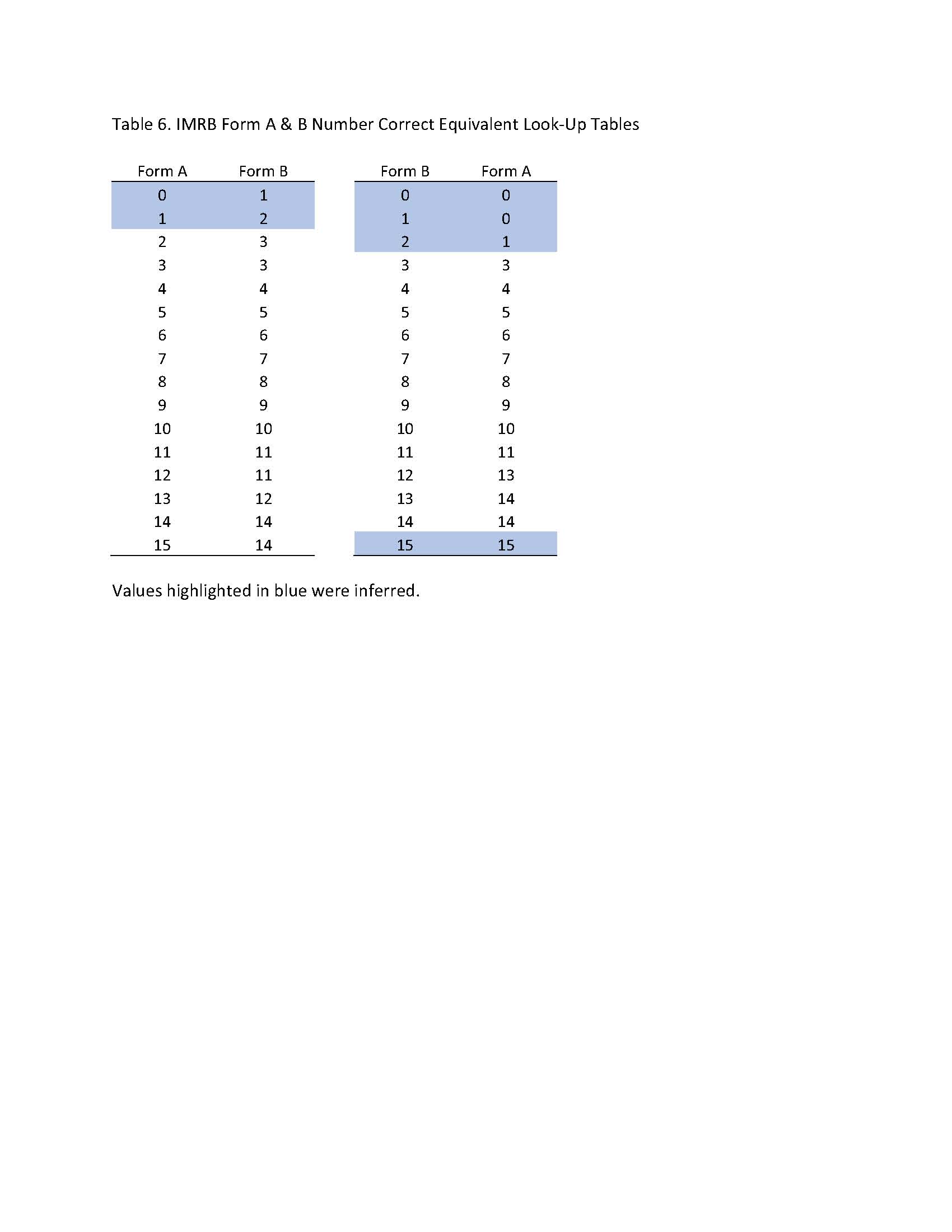

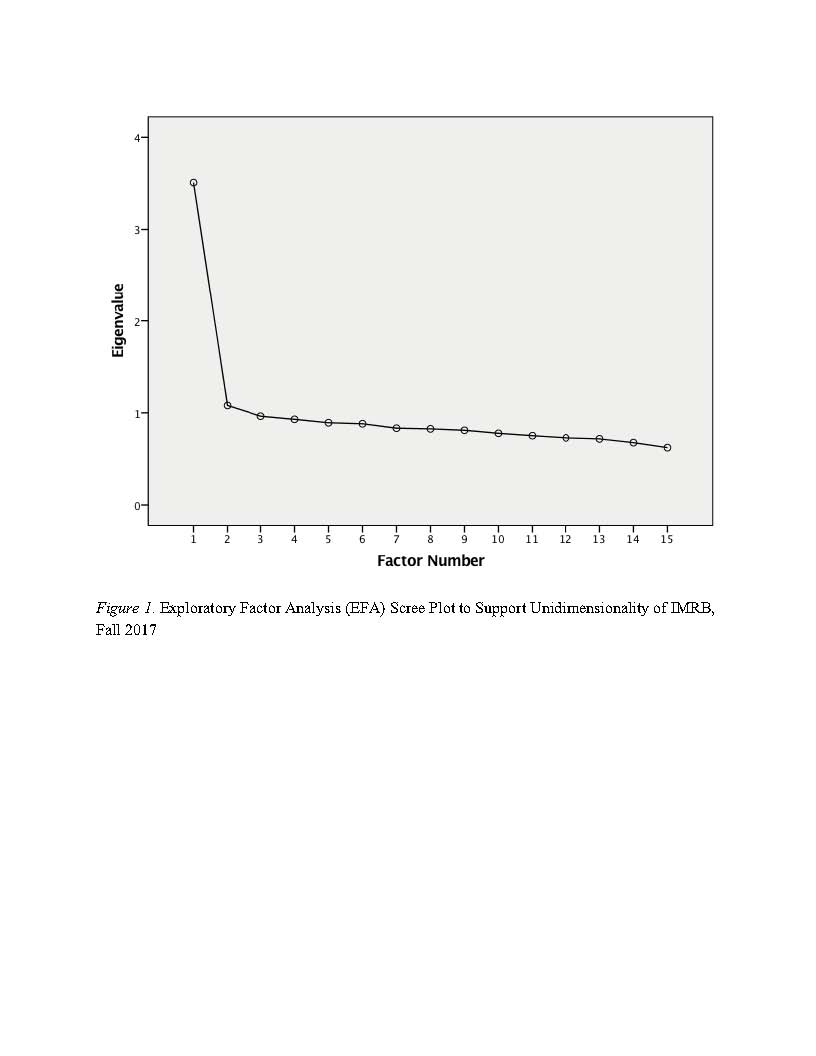

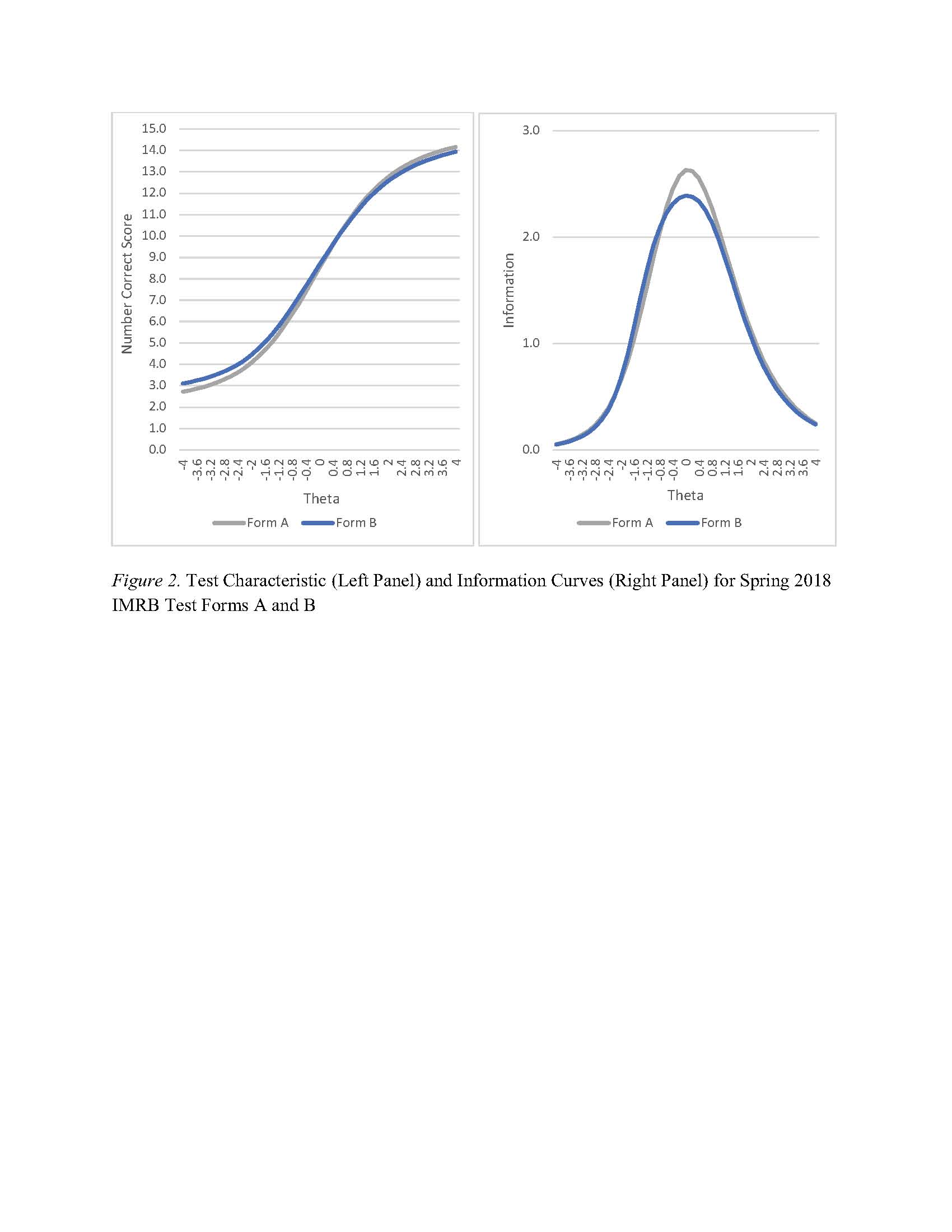

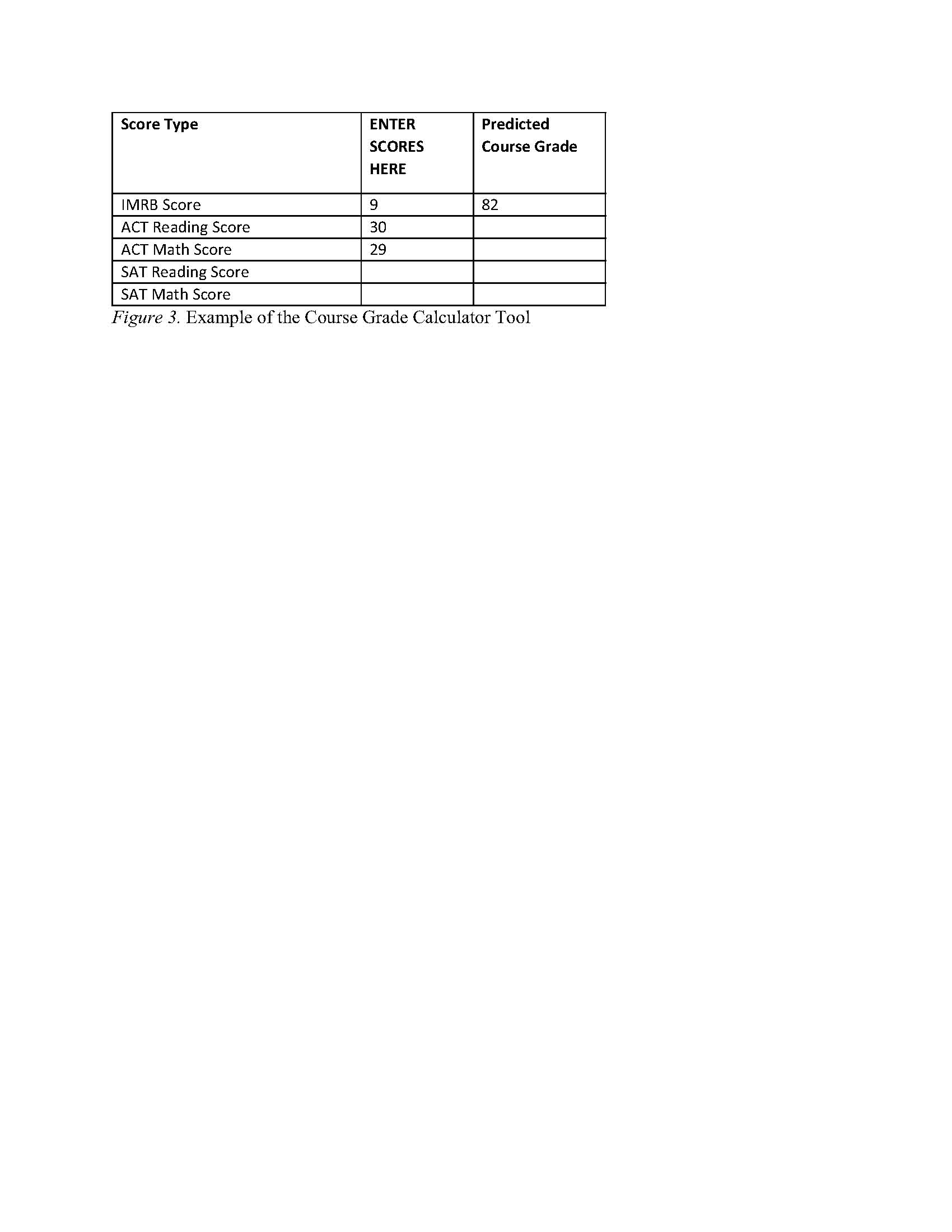

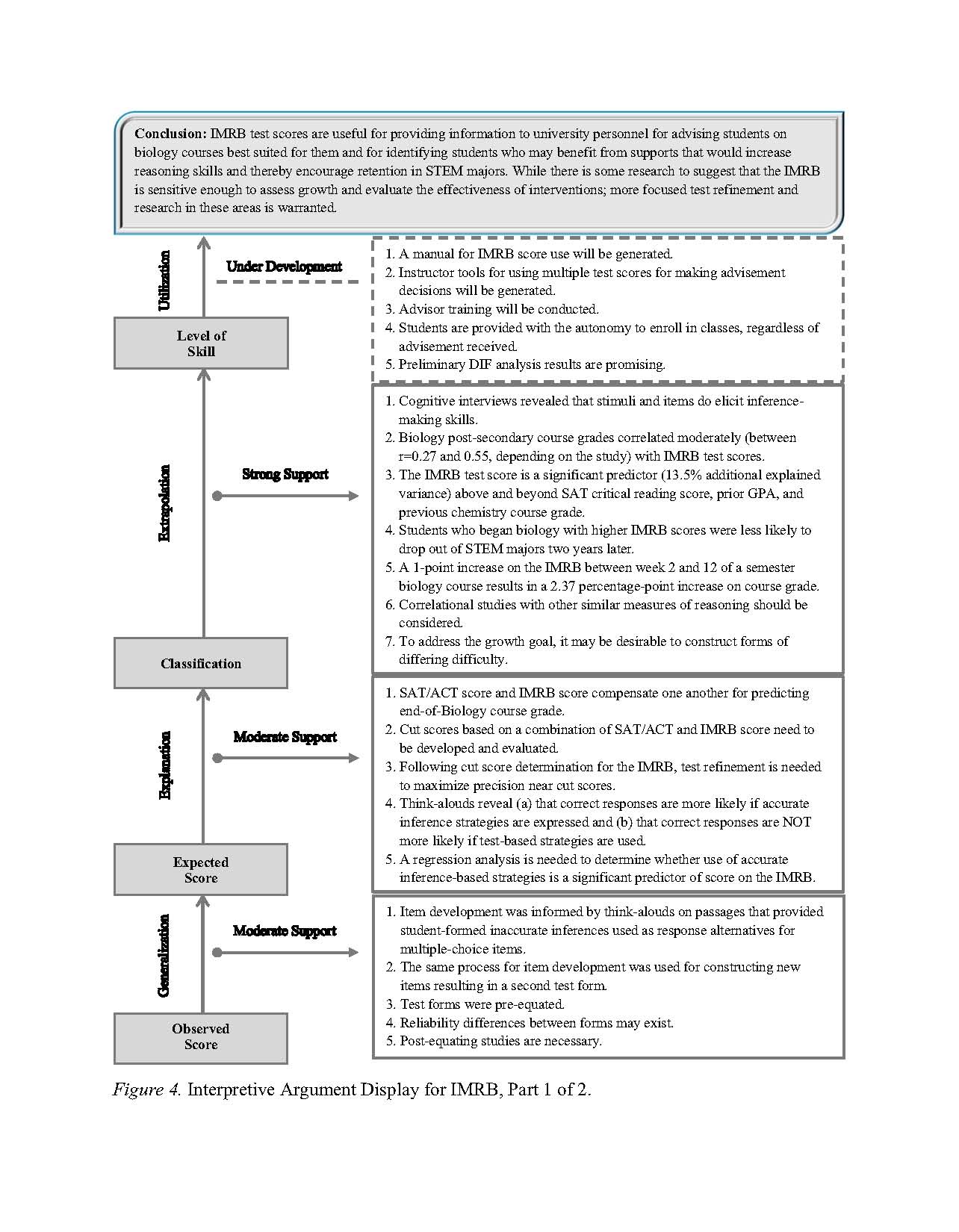

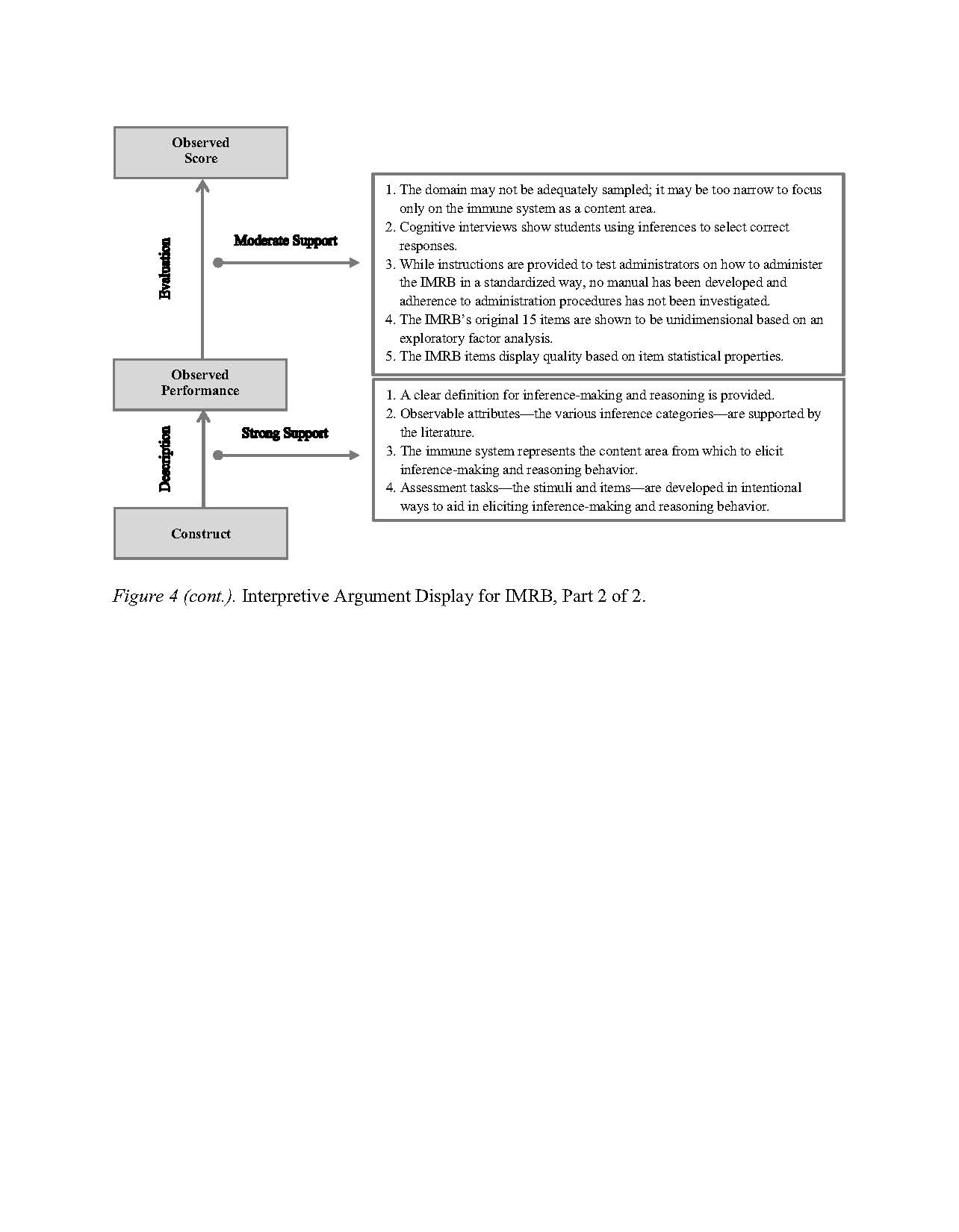


Appendix 3:
Course Grade Calculator


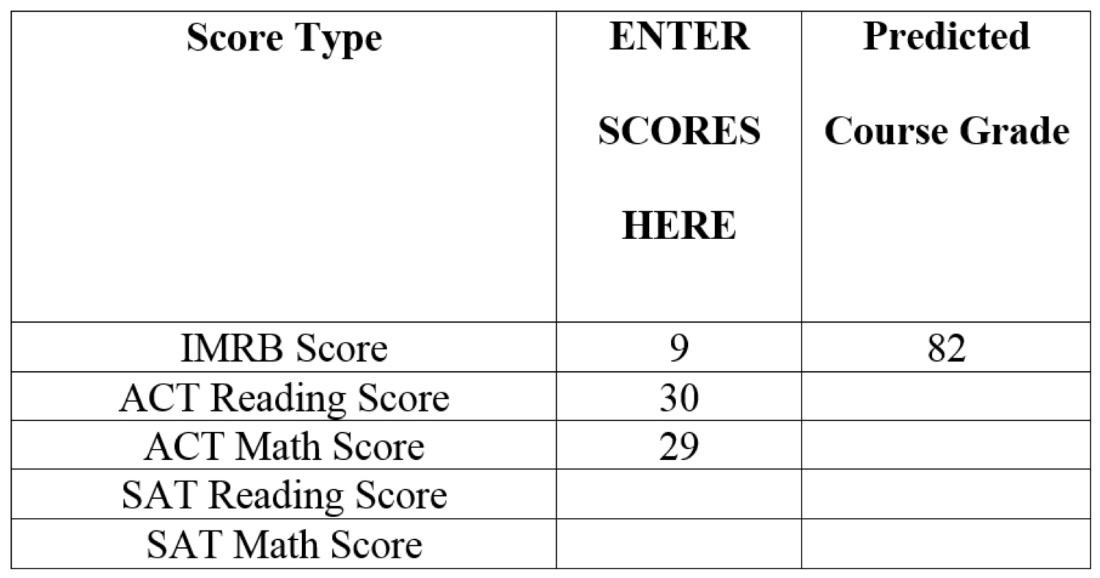


## COURSE GRADE CALCULATOR

The Course Grade Calculator is a spreadsheet-based tool designed for use by university personnel. Available scores can be entered, and the most appropriate prediction model will be selected and implemented to obtain a student’s predicted course grade. Once scores are input, the calculator applies the most appropriate linear regression weights to predict the final introductory biology course grade for a student. The tool looks similar to the image above. The example provided indicates a student earned a score of 9 on the IMRB, 30 on the ACT Reading test, and 29 on the ACT Math test, resulting in a predicted course grade of an 82. Note that the IMRB can also be used in the absence of SAT/ACT scores to predict course grades with efficacy; however, the most accurate predictions result when either SAT or ACT scores are also available. This online tool can be found online at this link: <http://hdl.handle.net/2142/108386>

1. https://www.act.org/content/dam/act/unsecured/documents/ACT-SAT-Concordance-Tables.pdf [↑](#footnote-ref-1)
